# Highly regioselective synthesis of lactams *via* cascade reaction of α,β-unsaturated ketones, ketoamides, and DBU as a catalyst†

**DOI:** 10.1039/d2ra07117g

**Published:** 2023-02-06

**Authors:** Xin Qin, Jinhai Zhang, Zhan-Yong Wang, Yimei Song, Yixiao Yang, Wenhai Zhang, Hongxin Liu

**Affiliations:** a College of Chemistry and Materials Engineering, Wenzhou University Wenzhou 325035 P. R. China hongxin-107@163.com; b Institute of New Materials & Industrial Technology, Wenzhou University Wenzhou 325035 P. R. China; c School of Pharmacy, Xinxiang University Xinxiang 453003 P. R. China

## Abstract

Herein, the aldol/Michael cascade reaction on the β,γ-positions of α,β-unsaturated ketones with ketoamides to construct bicyclic lactams *via* DBU catalysis has been developed. The substrates were well-tolerated with high regio- and diastereoselectivities in moderate to good yields (32 examples). The control experiments revealed that the hydrogen of the amide was the key factor.

## Abstract 2

The β,γ-positions of α,β-unsaturated ketones were reacted with ketoamides, with the hydrogen of the amide as the key factor to construct lactams.

## Graphical abstract

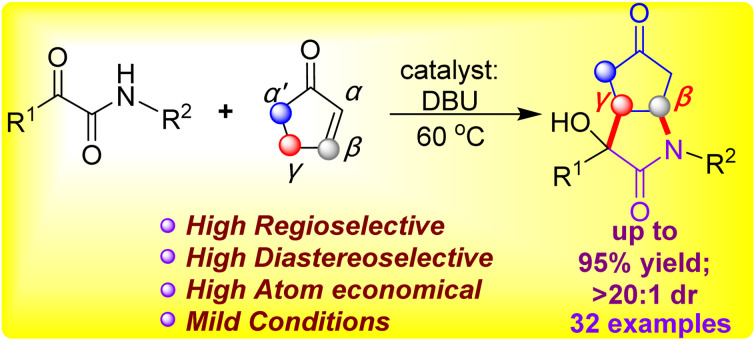

Development of efficient synthetic strategies for constructing structural motifs of natural products and bioactive molecules is of great interest in organic synthesis.^1^ Cascade reactions are among the most powerful means to generate molecular complexity from relatively simple materials.^2,3^ These transformations become even more attractive when multiple rings are formed during the process. Additionally, cascade reactions are atom-economical, environment-friendly and time efficient, rendering it an ideal strategy to build important structural motifs.^4,5^

Lactams and bicyclic lactams were privileged scaffolds presented in numerous natural products and pharmaceuticals.^6^ It is significant to develop new synthesis pathways to construct Lactams and bicyclic lactams with high regioselectivity and high atom-economical by cascade reaction. α,β-Unsaturated ketone is one of most important potential synthons in natural products synthesis^7–11^ and have multiple activation sites^12–15^ (α′, α, β, γ, γ′ Fig. 1a). In recent years, some research progress have been reported. The α′-position could activate as nucleophilic sites in some addition reactions *via* aldol reaction^16–18^ and some others.^19^ The direct α-functionalization *via* the Morita–Baylis–Hillman (MBH) reaction with some reports.^20–24^ The β-position could be attacked by nucleophiles with Michael reaction as a classic synthesis strategy.^25–30^ Except for α′, α, β-positions with good progress, the high regioselective of γ-functionalization to tune efficiently has been achieved.^31–34^ Since Melchiorre and Bencivenni described the γ-functionalization with vinylogous Michael addition of α,β-unsaturated ketone by amino-catalyzed in 2010.^35^ Ye developed the direct γ-functionalization *via* the path of [4 + 2] cycloaddition/retro-Mannich reaction, while the poor γ′/γ-regioselectivity remained to be solved by dienamine activation.^36^ Subsequently, regioselective Michael addition between β-substituted-cyclohexenones and nitroalkenes was explored.^32^ In addition, some bifunctionalizations to obtain cycloadducts were reported by Chen and Jørgensen *et al.* from the α′,γ-positions,^37^ α′,γ′-positions,^23^ β,γ-positions,^38–43^ γ,γ′-positions^44^ and others^45–47^ in cascade reaction of α,β-unsaturated ketones. In spite of many catalytic methodologies available for the functionalization of α,β-unsaturated ketones at their α, α′, β, γ, γ′ positions and multiple reaction sites with a variety of substrates. As a nucleophilic donor, the α′ and γ positions of α,β-unsaturated ketones could be well activated under base conditions. It is challenging to activate a single site of α′ or γ positions (Fig. 1b).

**Fig. 1:**
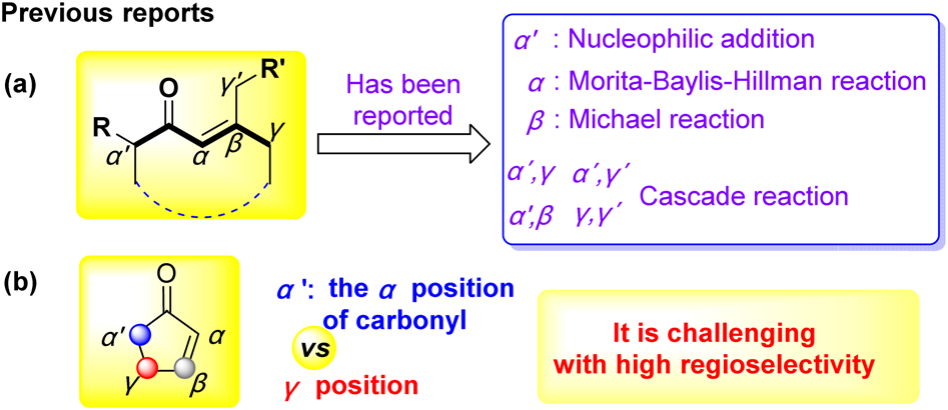
Regioselectivity functionalization of α,β-unsaturated ketones.

Therefore, highly efficient synthetic strategy to access the lactams and bicyclic lactams from the β and γ sites of α,β-unsaturated ketones unit are in great demand. To the best of our knowledge, it's no progress so far has been achieved high regioselectivity in the β and γ-positions with ketoamides to construct lactams and bicyclic lactams directly. Numerous natural products and pharmacologically active compounds contain the structure of the bicyclic lactams with cyclopentanone scaffold, showing the importance of this structural motif in synthetic organic chemistry.^6^ Based on our previous research on ketoamides,^48,49^ herein, we presented a novel high region-, diastereoselective and atom economical to synthesis of bicyclic lactams *via* the cascade reaction of aldol–Michael addition on the β and γ-positions of cyclopentenone with ketoamides by DBU catalyzed (Scheme 1).

**Scheme 1:**
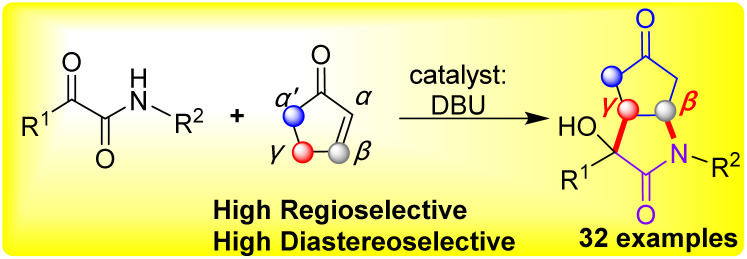
High regioselective of β,γ-positions of cyclopentenone with ketoamides to construct bicyclic lactams.

At the onset of this study, exploratory investigations towards our envisioned the aldol–Michael cascade reaction of β,γ-positions of cyclopentenone 2a with ketoamide 1a as the model reaction to optimize reaction conditions (Table 1). Firstly, a variety of common bases as catalysts was investigated in toluene at 60 °C for 12 hours. In the presence of 20 mol% of DABCO, pyrrolidine, NaOAc, DMAP and TMG were unable to promote the reaction (entries 1–5). It was interesting to note that when DBU as the catalyst, the desired product of aldol–Michael [3 + 2]-adduct on the β,γ-positions of 3a was observed with high regioselective, high diastereoselective (dr > 20 : 1) and moderate yield (entry 6, 48%). Subsequently, TBD, MTBD and DBN as catalyst, the desired product was observed with lower yield (entries 7–9). Encouraged by these promising results, with DBU as catalyst in hand, a series of solvents were screened to further improved the yield. Among these investigations, tetrahydrofuran (THF) as solvent better than other solvents obviously could increase the yield up to 64% (entry 14). But the remarkable thing was that this reaction didn't occur in 1,4-dioxane (entry 15). Subsequently, equivalent ratio, temperature, additives, catalyst loading, solvent volume and reaction time were screening to further improve the yield (entries 18–26). Finally, the yield creased up to 84% under the reaction of entry 24. Therefore, the screening studies clearly demonstrated that the reaction conditions shown in entry 24 were chosen as the optimized one.

**Table 1:** Optimization of the reaction conditions^a^

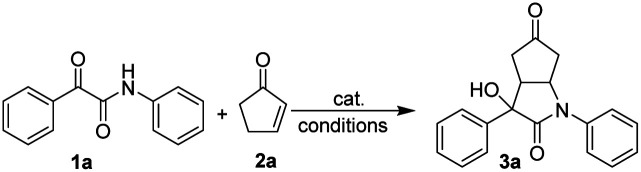				
Entry	Catalyst^k^	Solvent^l^	Time (h)	Yield^b^ (%)
1	DABCO	Toluene	12	NR
2	Pyrrolidine	Toluene	12	Trace
3	NaOAc	Toluene	12	NR
4	DMAP	Toluene	12	NR
5	TMG	Toluene	12	Trace
6	DBU	Toluene	12	48
7	TBD	Toluene	12	22
8	MTBD	Toluene	12	18
9	DBN	Toluene	12	28
10	DBU	DCM	12	28
11	DBU	EA	12	59
12	DBU	MeOH	12	25
13	DBU	MeCN	12	46
14	DBU	THF	12	64
15	DBU	1,4-Dioxane	12	NR
16	DBU	DMSO	12	20
17	DBU	H_2_O	12	NR
18	DBU	THF	24	82
19^c^	DBU	THF	24	80
20^c^^,^^d^	DBU	THF	24	79
21^e^	DBU	THF	24	77
22^f^	DBU	THF	24	59
23^f^^,^^g^	DBU	THF	12	82
24^f^^,^^g^^,^^h^	DBU	THF	12	84
25^f^^,^^g^^,^^i^	DBU	THF	12	55
26^g^^,^^j^	DBU	THF	24	24

a Unless otherwise noted, the reaction was carried out with 1a (0.1 mmol), 2a (0.3 mmol), catalyst (20 mol%), solvent (1.0 mL) at 60 °C.

b Isolated yields, dr > 20 : 1, dr values were measured by crude HNMR.

c 2a (0.5 mmol).

d 40 °C.

e Additive: 4 Å molecular sieve.

f Catalyst (10 mol%).

g Solvent (0.5 mL).

h 2a (0.15 mmol).

i 2a (0.1 mmol).

j Catalyst (5 mol%).

k DABCO = 1,4-diazabicyclo[2.2.2]octane, DMAP = 4-dimethylamino-pyridine, TMG = 1,1,3,3-tetramethylguanidine, TBD = 1,5,7-triaza-bicyclo[4.4.0]dec-5-ene, MTBD = 7-methyl-1,5,7-triazabi-cyclo[4.4.]dec-5-ene, DBN = 1,5-diazabicyclo[4.3.0]non-5-ene, DBU = 1,8-diazabicyclo-[5.4.0]undec-7-ene.

l DCM = dichloromethane, EA = ethyl acetate, THF = tetrahydrofuran, DMSO = dimethyl sulfoxide.

With the optimal reaction conditions had been determined, we next evaluated the scope of the aldol/Michael cascade reaction of β,γ-positions of cyclopentenone 2a to ketoamide derivatives 1. With respect to the ketoamides in this cascade reaction, the effect of different substituent groups and positions on the phenyl of 2-oxo-N,2-diphenylacetamide 1a were firstly investigated (Scheme 2). Regardless of their electronic properties and positions, including halides (F, Cl, Br), alkyl (Me) and alkoxy groups (–OMe) were well tolerated, and the desired products were obtained in moderate to good yields (3a–3l, 62–84%) with great regioselectivities and diastereoselectivities. Due to the steric effect, however, the *ortho*-substituted had noticeable effects with lower yield (3b, 62%) was observed even with longer reaction time, compared with *meta* and *para* substituted (3b*vs.*3c and 3h). Next, the substrates' scope of *N*-substituted was investigated. Bearing electron-withdrawing groups (halogen, CF_3_) and electron-donating groups (–OMe) at the *para* or *meta* position of the phenyl ring were examined. The corresponding aldol/Michael cascade reaction of β,γ-positions adducts were observed with good yields (3n–3aa, 76–85%). In addition, the results indicate the same trend that *ortho*-substituted with lower yield obvious than *meta* or *para* substituted (3m 53% *vs.*3t 77% and 3aa 79%). Moreover, except for substituents on the benzene ring, substrates with other substituents (for example, 2-naphthyl and 3-pyridyl) on the N of amide were also tested, and the substrates gave the corresponding products smoothly with moderate to good yields (3ab 75%, 3ac 57%). Furthermore, various alkyl substituents of R^1^ and R^2^ for 1 are well tolerated with 72–75% yields (3ad, 3ae).

**Scheme 2:**
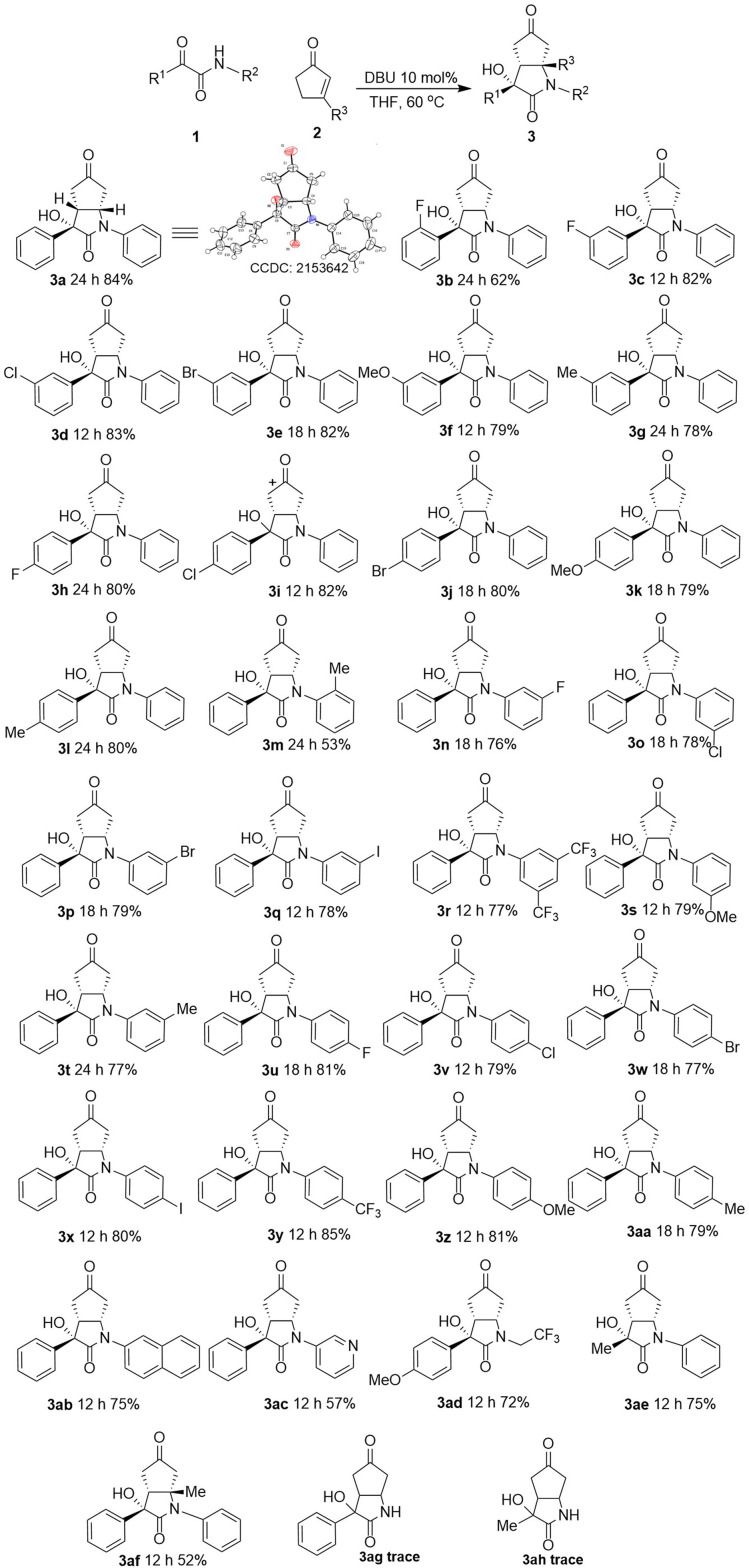
Aldol/Michael cascade reaction of β,γ-positions of cyclopentenones 2 to ketoamide derivatives 1. Reaction conditions: 1 (0.1 mmol), 2 (0.15 mmol), DBU (0.01 mmol, 10 mol%) in THF (0.5 mL) at 60 °C. Yield of the isolated product. Unless otherwise noted, dr > 20 : 1. The dr values were measured by crude HNMR.

To investigate the substrate scope further, β-methyl substituted in 3-methylcyclopent-2-en-1-one was used in the reaction, and the aldol/Michael cascade reaction of β,γ-positions proceeded smoothly with moderate yield (3af 52%). Moreover, in order to synthesize N–H free bicyclic lactam, 2-oxo-2-phenylacetamide and 2-oxopro-panamide were tested. Unfortunately, the substrates didn't afford the target products. Finally, the configuration of 3a was assigned by X-ray crystallographic analysis (CCDC: 2153642†).^50^

To further demonstrate the synthetic potential of the aldol/Michael cascade reaction of β,γ-positions of cyclopentenone with ketoamide to construct the bicyclic lactam, a gram-scale experiment was performed (Scheme 3a). With regarding to afford the corresponding product 3a with good result (82%). Control experiments were performed to provide mechanistic insight (Scheme 3b). Our initial attempts to treat *N*-methyl-2-oxo-*N*,2-diphenylacetamide 4 with cyclopentenone 2a did not work under standard conditions. The results potentially supporting the hydrogen on the amide of 1 were the key factor for this cascade reaction.

**Scheme 3:**
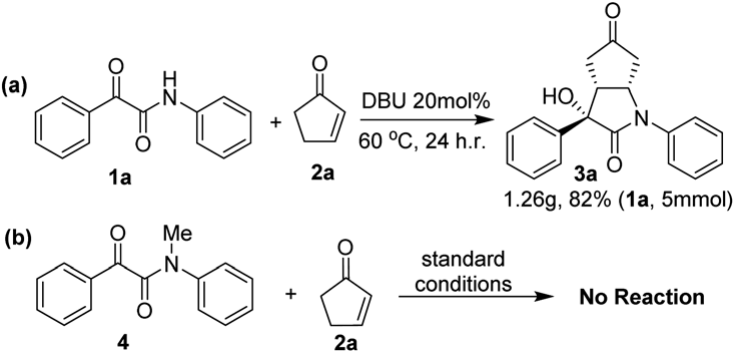
Gram-scale experiment and control experiment.

On the basis of our experimental results, a possible reaction mechanism involving a stepwise aldol/Michael cascade pathway was proposed as showed in Scheme 4. First, the reaction was initiated by deprotonation on the γ-site of cyclopentenone 2a to provide 2a′, then attacked the carbonyl of ketoamide 1 to form aldol adduct intermediate I. Subsequently, two possible processes occurred: The path I, intramolecular proton transfer through the five-membered ring transition state II in the amide bearing α-oxygen anion intermediate form the nitrogen anion intermediate IV. The path II, intermediate I was protonation to formed intermediate III firstly, and then deprotonation to formed IV. We couldn't track the intermediate II and III to verify the processes, however, both processes are possible could not be excluded. Subsequently, intramolecular aza-Michael addition IV on the β-site of cyclopentenone and then protonation to obtain cycloaddition product 3. Perhaps due to steric hindrance and thermodynamic influences, the ring α,β-unsaturated ketones provide the corresponding cascade products with good diastereoselectivities.

**Scheme 4:**
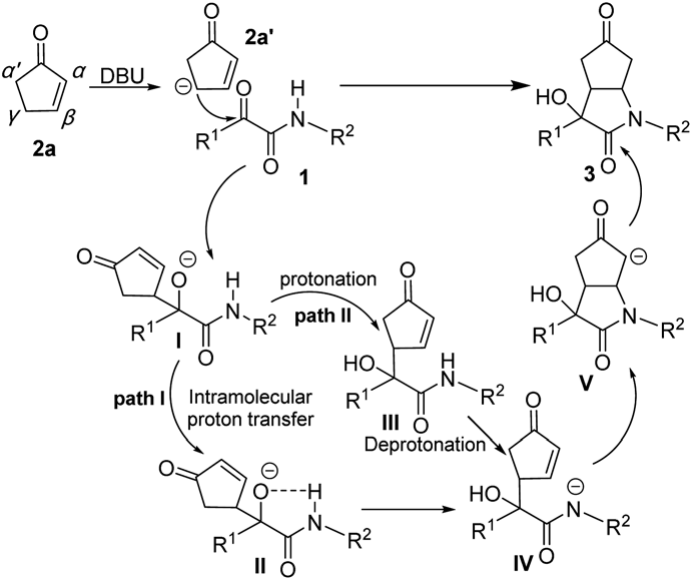
Proposed reaction mechanism of the cascade reaction.

## Conclusions

In summary, we have established a novel high region- and diastereoselective aldol/Michael cascade reaction on the β,γ-positions of α,β-unsaturated ketones with ketoamides to construct the bicyclic lactams with moderate to good yields under mild reaction conditions. Notably, this study presents the hydrogen of amide in ketoamide as a key role in this cascade reaction to the synthesis of lactams and bicyclic lactams containing quaternary carbon. Studies on extending the reaction type and enantioselectivity of this reaction are currently ongoing in our laboratory.

## Conflicts of interest

There are no conflicts to declare.

## Supplementary Material

RA-013-D2RA07117G-s001

RA-013-D2RA07117G-s002

## References

[cit1] Grondal C., Jeanty M., Enders D. (2010). Nat. Chem..

[cit2] Nicolaou K. C., Edmonds D. J., Bulger P. G. (2006). Angew. Chem., Int. Ed..

[cit3] Enders D., Hüttl M. R. M., Grondal C., Raabe G. (2006). Nature.

[cit4] Nicolaou K. C., Chen J. S. (2009). Chem. Soc. Rev..

[cit5] Volla C. M. R., Atodiresei I., Rueping M. (2014). Chem. Rev..

[cit6] Baranovskii A. V., Golubeva M. B. (2016). Chem. Nat. Compd..

[cit7] Liu Y., Han S.-J., Liu W.-B., Stoltz B. M. (2015). Acc. Chem. Res..

[cit8] Buter J., Moezelaar R., Minnaard A. J. (2014). Org. Biomol. Chem..

[cit9] Sacia E. R., Deaner M. H., Louie Y. L., Bell A. T. (2015). Green Chem..

[cit10] Wang Z. (2020). Org. Chem. Front..

[cit11] Zhou W., Voituriez A. (2021). J. Am. Chem. Soc..

[cit12] Notz W., Tanaka F., Barbas C. F. (2004). Acc. Chem. Res..

[cit13] Bertelsen S., Jørgensen K. A. (2009). Chem. Soc. Rev..

[cit14] Li J.-L., Liu T.-Y., Chen Y.-C. (2012). Acc. Chem. Res..

[cit15] Zhang L., Fu N., Luo S. (2015). Acc. Chem. Res..

[cit16] Mizuno M., Inoue H., Naito T., Zhou L., Nishiyama H. (2009). Chem.–Eur. J..

[cit17] Trost B. M., Shin S., Sclafani J. A. (2005). J. Am. Chem. Soc..

[cit18] Das J., Le Cavelier F., Rouden J., Blanchet J. (2011). Eur. J. Org. Chem..

[cit19] Chen Z.-C., Du W., Chen Y.-C. (2021). Chin. J. Chem..

[cit20] Gomes J. C., Sirvent J., Moyano A., Rodrigues M. T., Coelho F. (2013). Org. Lett..

[cit21] Raich L., Santos H., Gomes J. C., Rodrigues M. T., Galaverna R., Eberlin M. N., Coelho F., Rovira C., Moyano A. (2018). ACS Catal..

[cit22] Zhou Z., He Q., Jiang Y., Ouyang Q., Du W., Chen Y.-C. (2019). Org. Lett..

[cit23] Yang Q.-Q., Yin X., He X.-L., Du W., Chen Y.-C. (2019). ACS Catal..

[cit24] He Q., Yang Z.-H., Yang J., Du W., Chen Y.-C. (2020). Adv. Synth. Catal..

[cit25] Nising C. F., Bräse S. (2012). Chem. Soc. Rev..

[cit26] Hagiwara H. (2021). Nat. Prod. Commun..

[cit27] Song Y.-X., Du D.-M. (2021). Adv. Synth. Catal..

[cit28] Brown S. P., Goodwin N. C., MacMillan D. W. C. (2003). J. Am. Chem. Soc..

[cit29] Wang Y., Du D.-M. (2020). Org. Chem. Front..

[cit30] Yin Y., Jiang Z. (2017). ChemCatChem.

[cit31] Xie J.-K., Wang Y., Lin J.-B., Ren X.-R., Xu P.-F. (2017). Chem.–Eur. J..

[cit32] Zou C., Lv Y., Lu M., Li X., Zhang L., Yang L., Liu Z., Ke Y., Song G., Ye J. (2021). Org. Chem. Front..

[cit33] Wang Y., Lin J.-B., Xie J.-K., Lu H., Hu X.-Q., Xu P.-F. (2018). Org. Lett..

[cit34] Topolska A., Frankowski S., Albrecht Ł. (2022). Org. Lett..

[cit35] Bencivenni G., Galzerano P., Mazzanti A., Bartoli G., Melchiorre P. (2010). Proc. Natl. Acad. Sci. U. S. A..

[cit36] Zou C., Zeng C., Liu Z., Lu M., Sun X., Ye J. (2016). Angew. Chem., Int. Ed..

[cit37] Yin X., Zheng Y., Feng X., Jiang K., Wei X.-Z., Gao N., Chen Y.-C. (2014). Angew. Chem., Int. Ed..

[cit38] Zhou Z., Wang Z.-X., Zhou Y.-C., Xiao W., Ouyang Q., Du W., Chen Y.-C. (2017). Nat. Chem..

[cit39] Mose R., Preegel G., Larsen J., Jakobsen S., Iversen E. H., Jørgensen K. A. (2017). Nat. Chem..

[cit40] Zhou Z., Wang Z.-X., Ouyang Q., Xiao W., Du W., Chen Y.-C. (2017). Chem.–Eur. J..

[cit41] Xiao W., Yang Q.-Q., Chen Z., Ouyang Q., Du W., Chen Y.-C. (2018). Org. Lett..

[cit42] Gu X., Guo T., Dai Y., Franchino A., Fei J., Zou C., Dixon D. J., Ye J. (2015). Angew. Chem., Int. Ed..

[cit43] Xiao B.-X., Shi C.-H., Liang S.-Y., Jiang B., Du W., Chen Y.-C. (2019). Org. Lett..

[cit44] Sofiadis M., Kalaitzakis D., Sarris J., Montagnon T., Vassilikogiannakis G. (2019). Angew. Chem., Int. Ed..

[cit45] Yang Y., Zhu B., Zhu L., Jiang Y., Guo C.-L., Gu J., Ouyang Q., Du W., Chen Y.-C. (2021). Chem. Sci..

[cit46] Yang X.-C., Liu J.-Y., Liu Z., Hu X.-Q., Xu P.-F. (2019). J. Org. Chem..

[cit47] Yamazaki K., Gabriel P., Di Carmine G., Pedroni J., Farizyan M., Hamlin T. A., Dixon D. J. (2021). ACS Catal..

[cit48] Qin X., Wu C., Lu F., Wang Z.-Y., Jiang J., Liu H. (2022). ChemistrySelect.

[cit49] Wu C., Hu B., Liu H., Jiang J., Kim J. (2022). ChemistrySelect.

[cit50] CCDC 2153642 contains the ESI crystallographic data.†

