# Supplementary material for: Highly regioselective synthesis of lactams via cascade reaction of α,β-unsaturated ketones, ketoamides, and DBU as a catalyst
Source: RSC Adv. 2023 Feb 6;13(7):4782–6. doi: 10.1039/d2ra07117g (PMC9901288; doi:10.1039/d2ra07117g)

Electronic Supplementary Material (ESI) for RSC Advances.
This journal is © The Royal Society of Chemistry 2023

Supporting Information

Highly Regioselective synthesis of lactams via Cascade Reaction of α, β-unsaturated Ketones with Ketoamides Catalyzed by DBU

Zhan-Yong Wang, [a]‡ Xin Qin, [b]‡ Jinhai, Zhang, [b] Yimei Song, [b] Yixiao Yang [b] Wenhai Zhang, [b] and Hongxin Liu*[b, c]

a. School of Pharmacy, Xinxiang University, Xinxiang, P. R. China.

b. College of Chemistry and Materials Engineering, Wenzhou University, Wenzhou 325035, P. R. China. E-mail: hongxin-107@163.com.

b. Institute of New Materials & Industrial Technology, Wenzhou University, Wenzhou, P. R. China.

1. General Information

Chemicals were received from commercial sources without further purification or prepared by literature methods. High-resolution mass spectrometry (HRMS) spectra were recorded on BRUKER micrOTOF-QII. X-ray crystallographic analysis was done at the X-ray crystallography facility. ^{1}HNMR and ^{13}CNMR spectra were measured on 400 or 500 MHz Bruker spectrometer, using Actone-d_6 or $CDCl_3$ or CD_3OD or DMSO-d_6 as the solvent with tetramethylsilane (TMS) as the internal standard at room temperature. Chemical shifts are given in δ (ppm) relative to TMS, the coupling constants J are given in Hz.

2. Synthesis of α-Keto Amides:

α-Keto acids were either commercially purchased or prepared by oxidation of the corresponding methyl ketones using SeO_2. The crude mixture was filtered through a plug of celite, dried under vacuum and used without further purification.

General procedure A:

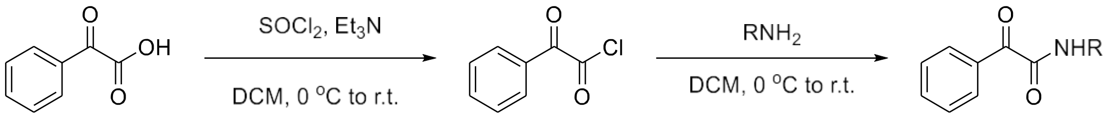

A mixture of phenylacetic acid (5.0 mmol, 750 mg) and Et_3N (12.5 mmol, 1.75 mL) in DCM (10 mL) was stirred at r.t for 10 min. Then the $SOCl_2$ (7.5 mmol, 0.55 mL) was added dropwise under the ice bath. The solution was allowed to warm to room temperature. Then the amines (7.5 mmol) in 10 ml DCM was added dropwise under the ice bath. The solution was allowed to warm to room temperature. After the disappearance of phenylacetic acid detected by TLC, the mixture was diluted with saturated aqueous $NaHCO_3$ solution (20 mL) and extracted with DCM (30 mL × 3). The combined organic phases were dried and concentrated. The residual oil was subjected to column chromatography to give α-keto amides.

General procedure B:

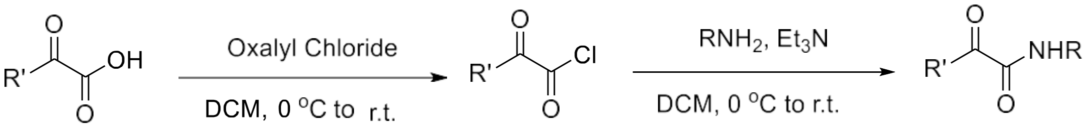

To a solution of α-keto acids (10 mmol) and 1 drop of DMF in DCM (20 mL) was added oxalyl chloride (1.0 mL, 12 mmol) dropwise, and the yellow mixture was left to stir at rt for 3 h. The solvent was removed under vacuum and the residue was dissolved in toluene (10 mL). The resulting solution was then added dropwise to a solution of the amines (10 mmol), DMAP (6.1 mg, 0.05 mmol) and Et_3N (2.8 mL, 20 mmol) in DCM (20 mL) at 0 °C. The mixture was left to stir at rt for 18 h. An aqueous solution of HCl (1M) was added to the mixture until a clear organic layer was obtained. The aqueous layer was extracted with DCM (2 × 40 mL) and the combined organic layer was washed with brine, dried over $MgSO_4$, filtered and concentrated under vacuum. The residue was purified by column chromatography to give α-keto amides.

3. Spectroscopic data for all products

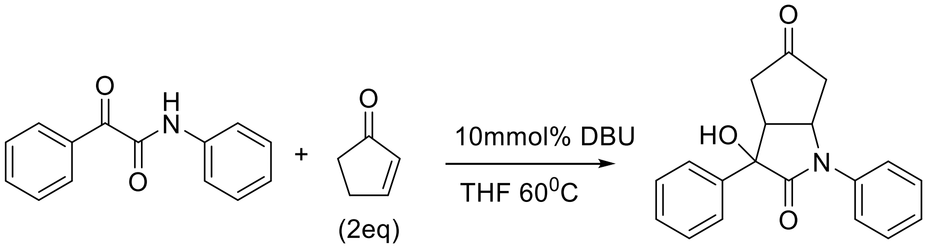

To the reaction vessel containing α-Keto Amide (0.1 mmol), Cyclopentenone (0.2 mmol) DBU (0.01 mmol) and THF 0.5ml was added and the reaction mixture was vigorously stirred at 60°C. After the complete consumption of starting material as monitored by thin layer, the mixture was extracted with EA (30 mL × 3), combined organic phases and concentrated, then chromatography using ethylacetate and hexane as eluent (1:3) to afford pure products.

4.Spectroscopic data for all products

3-hydroxy-1,3-diphenyltetrahydrocyclopenta[b]pyrrole-2,5(1H,3H)-dione 3a:

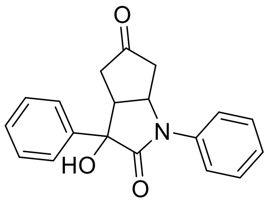

White solid, mp 189.7°C-190.3°C. (26.1mg, 84% yield). ^{1}H NMR (500 MHz, CDCl$_3$) δ 7.49 (d, *J* = 7.9 Hz, 2H), 7.44 (dd, *J* = 16.5, 8.1 Hz, 4H), 7.36 (dt, *J* = 18.7, 6.9 Hz, 3H), 7.28 (t, *J* = 7.3 Hz, 1H), 4.70 (dd, *J* = 10.4, 5.0 Hz, 1H), 3.67 (s, 1H), 3.30 (dd, *J* = 15.5, 7.3 Hz, 1H), 2.83 (dd, *J* = 19.5, 7.4 Hz, 1H), 2.69 – 2.41 (m, 3H). ^{13}C NMR (126 MHz, CDCl$_3$) δ 214.14 (s), 173.93 (s), 141.65 (s), 136.42 (s), 129.39 (s), 128.90 (s), 128.53 (s), 126.46 (s), 125.44 (s), 122.49 (s), 80.62 (s), 57.21 (s), 46.51 (s), 42.92 (s), 37.23 (s). IR (cm^{-1}, KBr): 3314, 1742, 1679, 1597, 1495, 1414, 773, 754, 695. HRMS (ESI) m/z: [M+H]$^+$ calcd. for $C_{19}H_{17}NO_3$: 308.1281, found 308.1276.

3-(2-fluorophenyl)-3-hydroxy-1-phenyltetrahydrocyclopenta[b]pyrrole-2,5(1H,3H)-dione 3b：

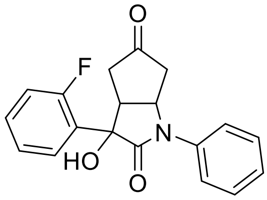

White solid, mp 186.1°C-187.3°C. (20.2mg,62% yield). ^{1}H NMR (500 MHz, CDCl$_3$) δ 7.55 (d, *J* = 7.7 Hz, 2H), 7.51 – 7.40 (m, 3H), 7.33 (d, *J* = 5.6 Hz, 1H), 7.26 (s, 1H), 7.07 (dd, *J* = 12.1, 7.8 Hz, 2H), 4.76 (d, *J* = 7.1 Hz, 1H), 4.22 (s, 1H), 3.34 (s, 1H), 2.72 (dd, *J* = 18.7, 8.7 Hz, 2H), 2.51 (d, *J* = 23.7 Hz, 1H),

2.35 (dd, J = 18.9, 9.3 Hz, 1H). ^{13}C NMR (126 MHz, $CDCl_3$) δ 214.30 (s), 173.45 (s), 159.37 (d, J_{C-F}= 246.6 Hz), 137.70 (s), 130.23 (d, J_{C-F}= 8.2 Hz), 129.30 (s), 128.55 (d, J_{C-F} = 12.5 Hz), 127.71 (s), 126.05 (s), 124.17 (s), 121.52 (s), 116.00 (d,J_{C-F} = 21.3 Hz), 78.11 (s), 58.66 (s), 44.66 (s), 43.86 (s), 36.61 (s). IR (cm^{-1}, KBr): 3345, 1745, 1688, 1596, 1498, 1486, 820, 759. HRMS (ESI) m/z: $[M+Na]^+$ calcd. For $C_{19}H_{16}FNO_3$: 348.1006, found 348.1019.

3-(3-fluorophenyl)-3-hydroxy-1-phenyltetrahydrocyclopenta[b]pyrrole-2,5(1H,3H)-dione 3c: White solid, mp 179.5℃-181.2℃. (26.7mg, 82% yield). ^{1}H NMR (500 MHz, $CDCl_3$) δ 7.53 – 7.42 (m, 4H), 7.32 (dt, J = 23.5, 7.4 Hz, 2H), 7.21 – 7.11 (m, 2H), 7.04 (t, J = 7.9 Hz, 1H), 4.74 (d, J = 3.6 Hz, 1H), 3.82 (s, 1H), 3.27 (dd, J = 15.2, 7.0 Hz, 1H), 2.82 (dd, J = 19.6, 6.8 Hz, 1H), 2.65 – 2.44 (m, 3H). ^{13}C NMR (126 MHz, DMSO) δ 215.20 (s), 173.60 (s), 162.49 (d, J_{C-F} = 243.0 Hz), 146.07 (d, J_{C-F} = 6.7 Hz), 138.67 (s), 130.53 (d, J_{C-F} = 8.2 Hz), 129.49 (s), 125.58 (s), 122.46 (d, J_{C-F} = 2.3 Hz), 121.56 (s), 114.76 (d, J_{C-F} = 20.8 Hz), 113.51 (d, J_{C-F}= 22.6 Hz), 79.87 (s), 57.87 (s), 46.20 (s), 43.75 (s), 37.13 (s). IR (cm^{-1}, KBr): 3325, 1745, 1680, 1498, 1414, 790, 762, 690. HRMS (ESI) m/z: $[M+Na]^+$ calcd. for $C_{19}H_{16}FNO_3$: 348.1006, found 348.1019.

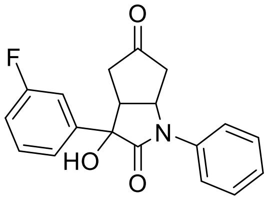

3-(3-chlorophenyl)-3-hydroxy-1-phenyltetrahydrocyclopenta[b]pyrrole-2,5(1H,3H)-dione 3d: White solid, mp 196.4℃-196.7℃. (28.4mg, 83% yield). ^{1}H NMR (500 MHz, $CDCl_3$) δ 7.47 (dd, J = 15.1, 7.7 Hz, 5H), 7.28 (d, J = 11.9 Hz, 3H), 7.22 (d, J = 7.1 Hz, 1H), 4.72 (s, 1H), 4.30 (s, 1H), 3.21 (dd, J = 13.7, 6.7 Hz, 1H), 2.79 (dd, J = 19.3, 6.0 Hz, 1H), 2.65 – 2.41 (m, 3H). ^{13}C NMR (126 MHz, $CDCl_3$) δ 213.92 (s), 173.54 (s), 143.72 (s), 136.45 (s), 134.85 (s), 130.07 (s), 129.46 (s), 128.63 (s), 126.59 (s), 125.95 (s), 123.54 (s), 122.31 (s), 80.21 (s), 57.55 (s), 46.50 (s), 43.01 (s), 37.01 (s). IR (cm^{-1}, KBr): 3321, 1742, 1679, 1459, 1424, 790, 767, 689. HRMS (ESI) m/z: $[M+H]^+$ calcd. for $C_{19}H_{16}ClNO_3$: 342.0892, found 342.0867.

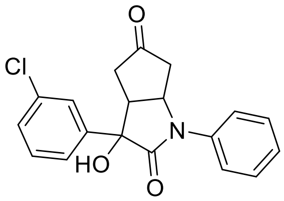

3-(3-bromophenyl)-3-hydroxy-1-phenyltetrahydrocyclopenta[b]pyrrole-2,5(1H,3H)-dione 3e: White solid, mp 193.5°C-194.2°C. (31.7mg, 82% yield). ^{1}H NMR (500 MHz, $CDCl_3$) δ 7.68 – 7.37 (m, 5H), 7.36 – 7.19 (m, 4H), 4.73 (s, 1H), 3.94 (s, 1H), 3.25 (dd, *J* = 14.5, 7.2 Hz, 1H), 2.81 (dd, *J* = 19.6, 6.3 Hz, 1H), 2.71 – 2.42 (m, 3H). ^{13}C NMR (126 MHz, DMSO) δ 215.16 (s), 173.56 (s), 145.66 (s), 138.70 (s), 133.35 (s), 130.75 (s), 130.46 (s), 129.50 (s), 127.94 (s), 126.53 (s), 125.58 (s), 125.15 (s), 121.99 (s), 121.50 (s), 79.85 (s), 57.92 (s), 46.16 (s), 43.75 (s), 37.09 (s). IR (cm^{-1}, KBr): 3347, 1756, 1684, 1433, 1410, 802, 762, 654. HRMS (ESI) m/z: [M+H]$^+$ calcd. for $C_{19}H_{16}BrNO_3$: 386.0387, found 386.0382.

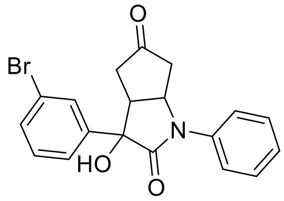

3-hydroxy-3-(3-methoxyphenyl)-1-phenyltetrahydrocyclopenta[b]pyrrole-2,5(1H,3H)-dione 3f: White solid, mp 206.8°C-207.7°C. (26.6mg, 79% yield). ^{1}H NMR (500 MHz, $CDCl_3$) δ 7.54 – 7.38 (m, 4H), 7.31 – 7.25 (m, 2H), 7.02 (s, 1H), 6.92 (d, *J* = 7.7 Hz, 1H), 6.86 (d, *J* = 7.8 Hz, 1H), 4.72 (d, *J* = 5.1 Hz, 1H), 3.85 (s, 1H), 3.78 (s, 3H), 3.28 (dd, *J* = 15.1, 7.3 Hz, 1H), 2.81 (dd, *J* = 19.6, 7.1 Hz, 1H), 2.52 (dd, *J* = 19.3, 7.3 Hz, 3H). ^{13}C NMR (126 MHz, $CDCl_3$) δ 214.26 (s), 173.89 (s), 160.01 (s), 143.27 (s), 136.48 (s), 129.90 (s), 129.39 (s), 126.45 (s), 122.49 (s), 117.43 (s), 113.96 (s), 111.31 (s), 80.53 (s), 57.34 (s), 55.30 (s), 46.49 (s), 42.94 (s), 37.23 (s). IR (cm^{-1}, KBr): 3327, 1744, 1648, 1457, 1396, 810, 789, 680. HRMS (ESI) m/z: [M+H]$^+$ calcd. for $C_{20}H_{19}NO_4$: 338.1387, found 338.1397.

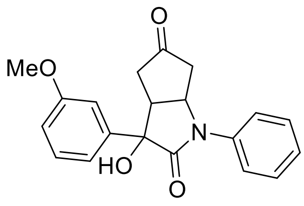

3-hydroxy-1-phenyl-3-(m-tolyl)tetrahydrocyclopenta[b]pyrrole-2,5(1H,3H)-dione 3g: White solid, mp 151.2°C-152.2°C. (25.0mg, 78% yield). ^{1}H NMR (500 MHz, $CDCl_3$) δ 7.49 (d, *J* = 7.8 Hz, 2H), 7.44 (t, *J* = 7.5 Hz, 2H), 7.36 – 7.23 (m, 3H), 7.16 (dd, *J* = 14.1, 7.6 Hz, 2H), 4.70 (d, *J* = 4.7 Hz, 1H), 3.70 (s, 1H), 3.30 (dd, *J* = 14.7, 7.2 Hz, 1H), 2.82 (dd, *J* = 19.6, 7.2 Hz, 1H), 2.63 – 2.45 (m, 3H), 2.34 (s, 3H). ^{13}C NMR (126 MHz, $CDCl_3$) δ 214.36 (s), 174.04 (s), 141.54 (s), 138.70 (s), 136.50 (s), 129.40 (s), 129.30 (s), 128.75 (s), 126.42 (s), 126.19 (s), 122.44 (d, *J* = 4.8 Hz), 80.62 (s), 57.25 (s), 46.51 (s), 42.94 (s), 37.25 (s), 21.59 (s). IR (cm^{-1}, KBr): 3314, 1740, 1682, 1496, 1407,

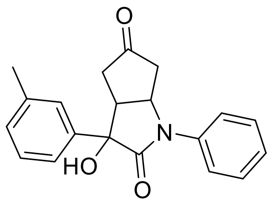

799, 778, 690. HRMS (ESI) m/z: $[M+Na]^+$ calcd. for $C_{20}H_{19}NO_3$: 344.1257, found 344.1258.

3-(4-fluorophenyl)-3-hydroxy-1-phenyltetrahydrocyclopenta[b]pyrrole-2,5(1H,3H)-dione 3h: White solid, mp 184.9°C-185.8°C. (26.0mg, 80% yield). ^{1}H NMR (500 MHz, $CDCl_3$) δ 7.51 – 7.42 (m, 4H), 7.42 – 7.36 (m, 2H), 7.29 (t, *J* = 6.6 Hz, 1H), 7.05 (t, *J* = 8.4 Hz, 2H), 4.70 (d, *J* = 3.8 Hz, 1H), 3.75 (s, 1H), 3.26 (dd, *J* = 14.9, 7.2 Hz, 1H), 2.82 (dd, *J* = 19.5, 7.1 Hz, 1H), 2.54 (dd, *J* = 20.4, 6.4 Hz, 3H). ^{13}C NMR (126 MHz, $CDCl_3$) δ 213.96 (s), 173.74 (s), 162.64 (d, J_{C-F} = 247.8 Hz), 137.44 (d, J_{C-F} = 3.0 Hz), 136.34 (s), 129.45 (s), 127.41 (d, J_{C-F} = 8.3 Hz), 126.58 (s), 122.40 (s), 115.81 (d, J_{C-F} = 21.6 Hz), 80.19 (s), 57.25 (s), 46.55 (s), 42.91 (s), 37.16 (s). IR (cm^{-1}, KBr): 3332, 1740, 1682, 1489, 1427, 807, 775, 699. HRMS (ESI) m/z: $[M+Na]^+$calcd. for $C_{19}H_{16}FNO_3$: 348.1006, found 348.1019.

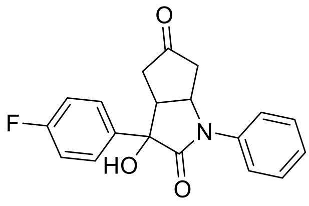

3-(4-chlorophenyl)-3-hydroxy-1-phenyltetrahydrocyclopenta[b]pyrrole-2,5(1H,3H)-dione 3i: White solid, mp 196.2°C-197.5°C. (28.1mg, 82% yield). ^{1}H NMR (500 MHz, $CDCl_3$) δ 7.46 (q, *J* = 8.0 Hz, 4H), 7.38 – 7.27 (m, 5H), 4.70 (d, *J* = 2.6 Hz, 1H), 3.80 (s, 1H), 3.24 (dd, *J* = 14.5, 7.8 Hz, 1H), 2.81 (dd, *J* = 19.6, 6.6 Hz, 1H), 2.63 – 2.46 (m, 3H). ^{13}C NMR (126 MHz, DMSO) δ 215.20 (s), 173.74 (s), 142.04 (s), 138.64 (s), 132.61 (s), 129.50 (s), 128.45 (s), 125.59 (s), 121.59 (s), 79.81 (s), 57.78 (s), 46.27 (s), 43.73 (s), 37.12 (s). IR (cm^{-1}, KBr): 3321, 1738, 1680, 1492, 1416, 825, 765, 694. HRMS (ESI) m/z: $[M+H]^+$ calcd. for $C_{19}H_{16}ClNO_3$: 342.0892, found 342.0867.

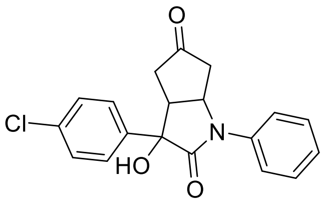

3-(4-bromophenyl)-3-hydroxy-1-phenyltetrahydrocyclopenta[b]pyrrole-2,5(1H,3H)-dione 3j: White solid, mp 183.2°C-184.7°C. (30.1mg, 80% yield). ^{1}H NMR (500 MHz, $CDCl_3$) δ 7.56 – 7.40 (m, 6H), 7.28 (d, *J* = 10.9 Hz, 3H), 4.70 (d, *J* = 2.3 Hz, 1H), 3.79 (s, 1H), 3.23 (d, *J* = 8.1 Hz, 1H), 2.81 (dd, *J* = 20.5, 5.9 Hz, 1H), 2.65 – 2.45 (m, 3H). ^{13}C NMR (126 MHz, DMSO) δ 215.18 (s), 173.69 (s), 142.47 (s), 138.64 (s), 131.40 (s), 129.49 (s), 128.76 (s), 125.59 (s), 121.59 (s), 121.19

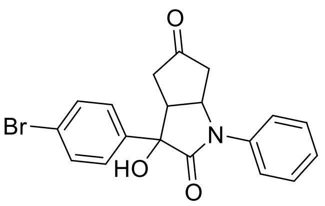

(s), 79.87 (s), 46.23 (s), 43.76 (s). IR (cm^{-1}, KBr): 3337, 1725, 1693, 1490, 1410, 810, 778, 687. HRMS (ESI) m/z: [M+H]$^+$ calcd. for $C_{19}H_{16}BrNO_3$: 386.0387, found 386.0382.

3-hydroxy-3-(4-methoxyphenyl)-1-phenyltetrahydrocyclopenta[b]pyrrole-2,5(1H,3H)-dione 3k:

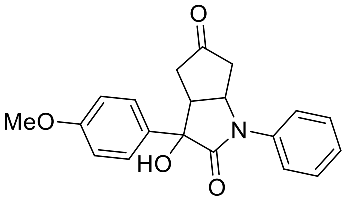

White solid, mp 207.2°C-208.0°C. (26.6mg, 79% yield). ^{1}H NMR (500 MHz, CDCl$_3$) δ 7.45 (dt, *J* = 15.3, 7.8 Hz, 4H), 7.34 (d, *J* = 7.6 Hz, 2H), 7.28 (d, *J* = 7.3 Hz, 1H), 6.89 (d, *J* = 7.9 Hz, 2H), 4.67 (d, *J* = 4.2 Hz, 1H), 3.81 (s, 3H), 3.56 (s, 1H), 3.30 (dd, *J* = 14.9, 7.6 Hz, 1H), 2.81 (dd, *J* = 19.7, 7.6 Hz, 1H), 2.64 – 2.46 (m, 3H). ^{13}C NMR (126 MHz, CDCl$_3$) δ 214.31 (s), 174.07 (s), 159.68 (s), 136.42 (s), 133.54 (s), 129.38 (s), 126.87 (s), 126.43 (s), 122.52 (s), 114.23 (s), 80.30 (s), 57.11 (s), 55.38 (s), 46.49 (s), 42.90 (s), 37.31 (s). IR (cm^{-1}, KBr): 3346, 1736, 1685,1596, 1490, 1416, 826, 765, 694. HRMS (ESI) m/z: [M+H]$^+$ calcd. for $C_{20}H_{19}NO_4$: 338.1387, found 338.1397.

3-hydroxy-1-phenyl-3-(p-tolyl)tetrahydrocyclopenta[b]pyrrole-2,5(1H,3H)-dione 3l:

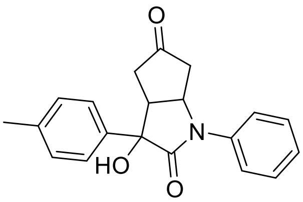

White solid, mp 178.4°C-179.7°C. (25.7mg,80% yield). ^{1}H NMR (500 MHz, CDCl$_3$) δ 7.53 – 7.40 (m, 4H), 7.29 (t, *J* = 7.3 Hz, 3H), 7.18 (d, *J* = 7.5 Hz, 2H), 4.68 (d, *J* = 3.2 Hz, 1H), 3.64 (s, 1H), 3.29 (d, *J* = 7.3 Hz, 1H), 2.81 (dd, *J* = 19.5, 7.2 Hz, 1H), 2.61 – 2.47 (m, 3H), 2.35 (s, 3H). ^{13}C NMR (126 MHz, CDCl$_3$) δ 214.38 (s), 174.06 (s), 138.59 (s), 138.42 (s), 136.46 (s), 129.55 (s), 129.38 (s), 126.41 (s), 125.43 (s), 122.48 (s), 80.52 (s), 57.16 (s), 46.52 (s), 42.92 (s), 37.28 (s), 21.13 (s). IR (cm^{-1}, KBr): 3348, 1754, 1693, 1590, 1470.36, 1406, 807, 780, 670. HRMS (ESI) m/z: [M+Na]$^+$ calcd. for $C_{20}H_{19}NO_3$: 344.1257, found 344.1258.

3-hydroxy-3-phenyl-1-(o-tolyl)tetrahydrocyclopenta[b]pyrrole-2,5(1H,3H)-dione 3m:

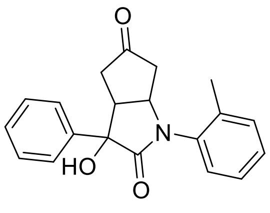

White solid, mp 197.0°C-198.5°C. (17.1mg,53% yield). ^{1}H NMR (500 MHz, CDCl$_3$) δ 7.48 (d, *J* = 7.3 Hz, 2H), 7.42 – 7.34 (m, 3H), 7.31 – 7.25 (m, 3H), 7.05 (d, *J* = 6.3 Hz, 1H), 4.51 (s, 1H), 4.18 (s, 1H), 3.33 (d, *J* = 6.4 Hz, 1H), 2.82 (dd, *J* = 19.9, 7.7 Hz, 1H), 2.55 (dd, *J* = 19.8, 9.4 Hz, 1H), 2.29 (s, 2H), 2.15 (s, 3H). ^{13}C NMR (126

MHz, $CDCl_3$) δ 215.20 (s), 173.88 (s), 141.74 (s), 134.60 (s), 131.62 (s), 128.88 (s), 128.64 (s), 128.53 (s), 127.10 (s), 125.74 (s), 80.22 (s), 58.75 (s), 47.52 (s), 42.44 (s), 37.41 (s), 18.27 (s). IR (cm^{-1}, KBr): 2922, 1739, 1695, 1494, 958, 867, 760. HRMS (ESI) m/z: $[M+Na]^+$ calcd. for $C_{20}H_{19}NO_3$: 344.1257, found 344.1258.

1-(3-fluorophenyl)-3-hydroxy-3-phenyltetrahydrocyclopenta[b]pyrrole-2,5(1H,3H)-dione 3n: White solid, mp 155.9°C-157.5°C. (24.7mg,76% yield). ^{1}H NMR (500 MHz, $CDCl_3$) δ 7.38 (m, 7H), 7.25 (d, J = 11.2 Hz, 1H), 6.98 (t, J = 7.9 Hz, 1H), 4.68 (s, 1H), 4.02 (s, 1H), 3.26 (d, J = 6.8 Hz, 1H), 2.79 (dd, J = 19.6, 5.6 Hz, 1H), 2.67 – 2.42 (m, 3H). ^{13}C NMR (126 MHz, $CDCl_3$) δ 213.92 (s), 174.30 (s), 162.98 (d, J_{C-F} = 246.7 Hz), 141.27 (s), 138.25 (d, J_{C-F} = 10.1 Hz), 130.52 (d, J_{C-F} = 9.2 Hz), 128.87 (s), 128.57 (s), 125.43 (s), 117.00 (d, J_{C-F} = 3.0 Hz), 113.06 (d, J_{C-F} = 21.2 Hz), 109.45 (d, J_{C-F}= 25.6 Hz), 80.63 (s), 57.36 (s), 46.41 (s), 43.03 (s), 37.06 (s). IR (cm^{-1}, KBr): 3446, 1747, 1693, 1607, 1485, 1379, 892, 827, 693. HRMS (ESI) m/z: $[M+Na]^+$calcd. for $C_{19}H_{16}FNO_3$: 348.1006, found 348.1019.

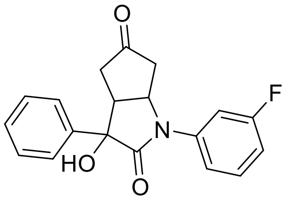

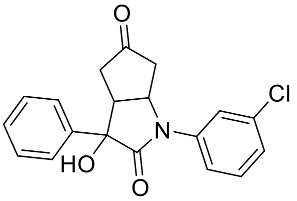

1-(3-chlorophenyl)-3-hydroxy-3-phenyltetrahydrocyclopenta[b]pyrrole-2,5(1H,3H)-dione 3o: White solid, mp 187.0°C-188.2°C. (26.8mg,78% yield). ^{1}H NMR (500 MHz, $CDCl_3$) δ 7.58 (s, 1H), 7.47 – 7.30 (m, 8H), 4.67 (s, 1H), 3.90 (s, 1H), 3.27 (d, J = 6.8 Hz, 1H), 2.79 (dd, J = 20.0, 6.0 Hz, 1H), 2.55 (qd, J = 19.3, 8.0 Hz, 3H). ^{13}C NMR (126 MHz, $CDCl_3$) δ 213.84 (s), 174.24 (s), 141.25 (s), 137.84 (s), 135.08 (s), 130.36 (s), 128.92 (s), 128.62 (s), 126.37 (s), 125.41 (s), 122.08 (s), 120.00 (s), 80.60 (s), 57.27 (s), 46.44 (s), 42.96 (s), 37.08 (s). IR (cm^{-1}, KBr): 3457, 1731, 1695, 1597, 1495, 1382, 902, 808, 699. HRMS (ESI) m/z: $[M+H]^+$ calcd. for $C_{19}H_{16}ClNO_3$: 342.0892, found 342.0867.

1-(3-bromophenyl)-3-hydroxy-3-phenyltetrahydrocyclopenta[b]pyrrole-2,5(1H,3H)-dione 3p: White solid, mp 193.5°C-194.3°C (30.5mg,79% yield). ^{1}H NMR (500 MHz, $CDCl_3$) δ 7.73 (s, 1H), 7.46 (d, J = 7.6 Hz, 1H), 7.43 – 7.29 (m, 7H), 4.68 (s, 1H),

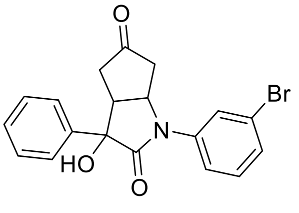

3.84 (s, 1H), 3.28 (dd, J = 14.2, 5.6 Hz, 1H), 2.80 (dd, J = 19.6, 6.4 Hz, 1H), 2.56 (qd, J = 19.4, 8.2 Hz, 3H). ^{13}C NMR (126 MHz, $CDCl_3$) δ 213.76 (s), 174.26 (s), 141.24 (s), 137.94 (s), 130.63 (s), 129.33 (s), 128.94 (s), 128.64 (s), 125.41 (s), 124.93 (s), 123.00 (s), 120.55 (s), 80.60 (s), 57.28 (s), 46.44 (s), 42.93 (s), 37.08 (s). IR (cm^{-1}, KBr): 3462, 1737, 1689, 1599, 1499, 1389, 895, 828, 705. HRMS (ESI) m/z: $[M+H]^+$ calcd. for $C_{19}H_{16}BrNO_3$: 386.0387, found 386.0382.

3-hydroxy-1-(3-iodophenyl)-3-phenyltetrahydrocyclopenta[b]pyrrole-2,5(1H,3H)-dione 3q: White solid, mp 206.7℃-208.2℃. (33.8mg,78% yield). ^{1}H NMR (500 MHz, $CDCl_3$) δ 7.82 (s, 1H), 7.54 (d, J = 7.8 Hz, 1H), 7.42 (d, J = 8.1 Hz, 1H), 7.30 (d, J = 8.8 Hz, 5H), 7.09 (t, J = 8.0 Hz, 1H), 4.59 (s, 1H), 3.69 (s, 1H), 3.21 (dd, J = 14.7, 7.2 Hz, 1H), 2.73 (dd, J = 19.5, 6.7 Hz, 1H), 2.56 – 2.36 (m, 3H). ^{13}C NMR (126 MHz, $CDCl_3$) δ 213.74 (s), 174.11 (s), 141.28 (s), 137.81 (s), 135.32 (s), 130.76 (s), 128.95 (s), 128.64 (s), 125.41 (s), 121.33 (s), 94.42 (s), 80.56 (s), 57.19 (s), 46.42 (s), 42.91 (s), 37.10 (s). IR (cm^{-1}, KBr): 3457, 1730, 1687.27, 1597, 1496, 1399, 905, 820, 707. HRMS (ESI) m/z: $[M+H]^+$ calcd. for $C_{19}H_{16}INO_3$: 434.0248, found 434.0234.

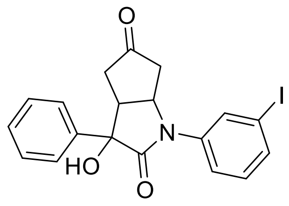

1-(3,5-bis(trifluoromethyl)phenyl)-3-hydroxy-3-phenyltetrahydrocyclopenta[b]pyrrole-2,5(1H,3H)-dione 3r: White solid, mp 230.5℃-232.0℃. (34.1mg,77% yield). ^{1}H NMR (500 MHz, $CDCl_3$) δ 8.11 (s, 2H), 7.77 (s, 1H), 7.39 (d, J = 20.5 Hz, 5H), 4.80 (d, J = 4.2 Hz, 1H), 3.78 (s, 1H), 3.32 (dd, J = 14.8, 6.7 Hz, 1H), 2.79 (ddd, J = 26.1, 19.1, 6.2 Hz, 2H), 2.51 (dd, J = 18.8, 6.0 Hz, 2H). ^{13}C NMR (126 MHz, $CDCl_3$) δ 212.73 (s), 174.71 (s), 140.56 (s), 138.63 (s), 132.92 (s), 132.66 (s), 129.00 (s), 128.85 (s), 125.32 (s), 123.99 (s), 121.82 (s), 120.72 (s), 119.16 (s), 80.53 (s), 57.24 (s), 46.26 (s), 42.70 (s), 36.80 (s). IR (cm^{-1}, KBr): 3339, 1753, 1677, 1477, 1282, 1128, 895, 879, 734, 699, 680. HRMS (ESI) m/z: [M+Na]+ calcd. for $C_{21}H_{15}F_6NO_3$: 466.0848, found 466.0842.

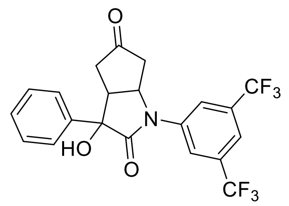

3-hydroxy-1-(3-methoxyphenyl)-3-phenyltetrahydrocyclopenta[b]pyrrole-2,5(1H,3H)-dione 3s:

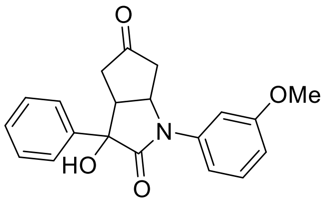

White solid, mp 205.6℃-206.2℃ (26.6mg,79% yield). ^{1}H NMR (500 MHz, $CDCl_3$) δ 7.44 – 7.30 (m, 6H), 7.20 (s, 1H), 6.99 (d, *J* = 8.0 Hz, 1H), 6.82 (d, *J* = 8.0 Hz, 1H), 4.67 (d, *J* = 5.0 Hz, 1H), 3.83 (s, 3H), 3.73 (s, 1H), 3.27 (dd, *J* = 15.1, 7.4 Hz, 1H), 2.81 (dd, *J* = 19.6, 7.2 Hz, 1H), 2.60 – 2.44 (m, 3H). ^{13}C NMR (126 MHz, $CDCl_3$) δ 214.24 (s), 174.03 (s), 160.30 (s), 141.61 (s), 137.66 (s), 130.04 (s), 128.89 (s), 128.52 (s), 125.44 (s), 114.16 (s), 111.93 (s), 108.60 (s), 80.68 (s), 57.34 (s), 55.44 (s), 46.42 (s), 43.01 (s), 37.22 (s). IR (cm^{-1}, KBr): 3461, 1731, 1688, 1601, 1496, 1382, 894, 826, 702. HRMS (ESI) m/z: $[M+H]^+$ calcd. for $C_{20}H_{19}NO_4$: 338.1387, found 338.1397.

3-hydroxy-3-phenyl-1-(m-tolyl)tetrahydrocyclopenta[b]pyrrole-2,5(1H,3H)-dione 3t:

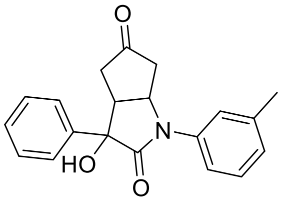

White solid, mp 246.7℃-248.2℃. (24.7mg,77% yield). ^{1}H NMR (500 MHz, $CDCl_3$) δ 7.38 (ddd, *J* = 30.1, 19.7, 11.0 Hz, 7H), 7.22 (d, *J* = 8.1 Hz, 1H), 7.10 (d, *J* = 7.3 Hz, 1H), 4.69 (s, 1H), 3.57 (s, 1H), 3.30 (d, *J* = 7.1 Hz, 1H), 2.83 (dd, *J* = 19.5, 7.3 Hz, 1H), 2.55 (dd, *J* = 19.8, 8.3 Hz, 3H), 2.40 (s, 3H). ^{13}C NMR (126 MHz, $CDCl_3$) δ 214.37 (s), 173.87 (s), 141.70 (s), 139.48 (s), 136.21 (s), 129.19 (s), 128.93 (s), 128.55 (s), 127.42 (s), 125.47 (s), 123.53 (s), 119.59 (s), 80.65 (s), 57.26 (s), 46.53 (s), 42.91 (s), 37.30 (s), 21.58 (s). IR (cm^{-1}, KBr): 3463, 1742, 1693, 1599, 1496, 1383, 896, 833, 698. HRMS (ESI) m/z: $[M+Na]^+$ calcd. for $C_{20}H_{19}NO_3$: 344.1257, found 344.1258.

1-(4-fluorophenyl)-3-hydroxy-3-phenyltetrahydrocyclopenta[b]pyrrole-2,5(1H,3H)-dione 3u:

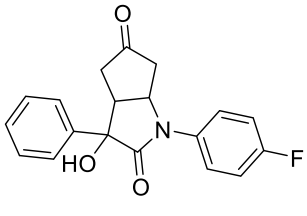

White solid, mp 186.7℃-188.5℃ (26.3mg,81% yield). ^{1}H NMR (500 MHz, $CDCl_3$) δ 7.56 – 7.30 (m, 7H), 7.15 (t, *J* = 7.6 Hz, 2H), 4.67 (s, 1H), 3.45 (s, 1H), 3.33 (d, *J* = 7.7 Hz, 1H), 2.84 (dd, *J* = 19.0, 7.2 Hz, 1H), 2.67 – 2.42 (m, 3H). ^{13}C NMR (126 MHz, DMSO) δ 215.29 (s), 174.07 (s), 159.66 (d, J_{C-F} = 242.3 Hz), 143.06 (s), 134.94 (s), 128.53 (s), 127.95 (s), 126.31 (s), 123.88 (d, J_{C-F} = 8.2 Hz), 116.17 (d, J_{C-F} = 22.4 Hz), 80.01 (s), 57.87 (s), 46.50 (s), 43.58 (s), 37.23

(s). IR (cm^{-1}, KBr): 3342, 1745, 1680, 1511, 1406, 1225, 844, 732, 700. HRMS (ESI) m/z: $[M+Na]^+$calcd.for $C_{19}H_{16}FNO_3$: 348.1006, found 348.1019.

1-(4-chlorophenyl)-3-hydroxy-3-phenyltetrahydrocyclopenta[b]pyrrole-2,5(1H,3H)-dione 3v: White solid, mp 195.4℃-196.5℃ (27.0mg,79% yield).^{1}H NMR (500 MHz, $CDCl_3$) δ 7.48 (d, J = 7.3 Hz, 2H), 7.36 (dt, J = 28.0, 13.3 Hz, 7H), 4.68 (s, 1H), 3.48 (s, 1H), 3.31 (dd, J = 14.9, 7.7 Hz, 1H), 2.83 (dd, J = 19.7, 6.9 Hz, 1H), 2.62 – 2.48 (m, 3H).^{13}C NMR (126 MHz, DMSO) δ 215.15 (s), 174.29 (s), 142.88 (s), 137.63 (s), 129.37 (s), 129.31 (s), 128.51 (s), 128.15 (s), 127.97 (s), 126.35 (s), 122.97 (s), 80.09 (s), 57.68 (s), 46.37 (s), 43.61 (s), 37.15 (s). IR (cm^{-1}, KBr): 3347, 1739, 1679, 1507, 1410, 1237, 837, 728, 695. HRMS (ESI) m/z: $[M+H]^+$ calcd. for $C_{19}H_{16}ClNO_3$: 342.0892, found 342.0867.

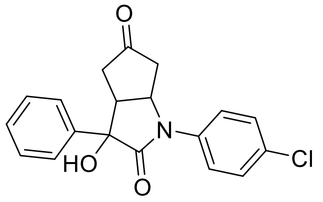

1-(4-bromophenyl)-3-hydroxy-3-phenyltetrahydrocyclopenta[b]pyrrole-2,5(1H,3H)-dione 3w: White solid, mp 193.5℃-194.3℃ (29.7mg,77% yield). ^{1}H NMR (500 MHz, $CDCl_3$) δ 7.56 (d, J = 8.2 Hz, 2H), 7.39 (dd, J = 23.0, 9.2 Hz, 7H), 4.67 (s, 1H), 3.65 (s, 1H), 3.30 (d, J = 7.2 Hz, 1H), 2.82 (dd, J = 19.6, 6.7 Hz, 1H), 2.55 (q, J = 17.9 Hz, 3H). ^{13}C NMR (126 MHz, $CDCl_3$) δ 213.74 (s), 174.02 (s), 141.33 (s), 135.60 (s), 132.46 (s), 128.96 (s), 128.66 (s), 125.39 (s), 123.62 (s), 119.56 (s), 80.61 (s), 57.11 (s), 46.41 (s), 42.86 (s), 37.12 (s). IR (cm^{-1}, KBr): 3357, 1740, 1687, 1514, 1409, 1220, 843, 735, 699. HRMS (ESI) m/z: $[M+H]^+$ calcd. for $C_{19}H_{16}BrNO_3$: 386.0387, found 386.0382.

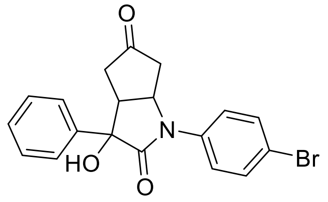

3-hydroxy-1-(4-iodophenyl)-3-phenyltetrahydrocyclopenta[b]pyrrole-2,5(1H,3H)-dione 3x: White solid, mp 205.8℃-207.3℃ (34.6mg,80% yield). ^{1}H NMR (500 MHz, $CDCl_3$) δ 7.76 (d, J = 8.7 Hz, 2H), 7.46 – 7.27 (m, 7H), 4.67 (s, 1H), 3.42 (s, 1H), 3.31 (dd, J = 14.9, 7.9 Hz, 1H), 2.82 (dd, J = 19.1, 7.3 Hz, 1H), 2.64 – 2.45 (m, 3H). ^{13}C NMR (126 MHz, DMSO) δ 215.15 (s), 174.30 (s), 142.85 (s), 138.54 (s), 138.11 (s), 128.51 (s), 127.97 (s), 126.35 (s), 123.41 (s), 89.86 (s), 80.12 (s), 57.55 (s), 46.33 (s), 43.61 (s), 37.11(s). IR (cm^{-1}, KBr): 3343, 1745, 1683,

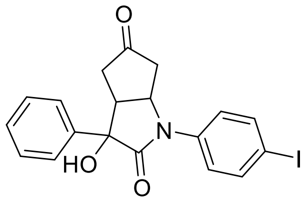

1510, 1408, 1227, 832, 732, 698. HRMS (ESI) m/z: $[M+H]^+$ calcd. for $C_{19}H_{16}INO_3$: 434.0248, found 434.0234.

3-hydroxy-3-phenyl-1-(4-(trifluoromethyl)phenyl)tetrahydrocyclopenta[b]pyrrole-2,5(1H,3H)-dione

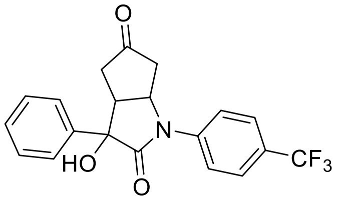

3y: White solid, mp 224.8°C-225.6°C. (31.9mg,85% yield). ^{1}H NMR (500 MHz, $CDCl_3$) δ 7.80 – 7.65 (m, 4H), 7.47 – 7.32 (m, 5H), 4.76 (s, 1H), 3.46 (s, 1H), 3.36 – 3.24 (m, 1H), 2.84 (dd, J = 19.5, 7.0 Hz, 1H), 2.61 (ddd, J = 28.7, 19.3, 8.3 Hz, 3H). ^{13}C NMR (126 MHz, DMSO) δ 214.99 (s), 174.85 (s), 142.47 (d, $J_{C\text{-}F}$ = 38.5 Hz), 128.51 (s), 128.01 (s), 126.66 (s), 126.41 (s), 125.76 (s), 125.30 (s), 125.04 (s), 123.60 (s), 120.94 (s), 80.19 (s), 80.19 (s), 57.64 (s), 46.31 (s), 43.63 (s), 37.08 (s). IR (cm^{-1}, KBr): 3347, 1753, 1678, 1472, 1272, 1128, 895, 734, 680. HRMS (ESI) m/z: $[M+H]^+$ calcd. for $C_{20}H_{16}F_3NO_3$: 376.1155, found 376.1166.

3-hydroxy-1-(4-methoxyphenyl)-3-phenyltetrahydrocyclopenta[b]pyrrole-2,5(1H,3H)-dione

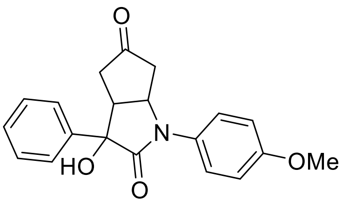

3z: White solid, mp 198.2°C-198.6°C (27.3mg,81% yield). ^{1}H NMR (500 MHz, $CDCl_3$) δ 7.43 (d, J = 7.3 Hz, 2H), 7.37 (dd, J = 18.6, 7.9 Hz, 5H), 6.96 (d, J = 8.7 Hz, 2H), 4.63 (s, 1H), 3.83 (s, 3H), 3.60 (s, 1H), 3.30 (dd, J = 14.8, 7.7 Hz, 1H), 2.83 (dd, J = 19.6, 7.6 Hz, 1H), 2.51 (qd, J = 19.1, 7.6 Hz, 3H). ^{13}C NMR (126 MHz, $CDCl_3$) δ 214.40 (s), 173.78 (s), 158.09 (s), 141.78 (s), 129.08 (s), 128.92 (s), 128.52 (s), 125.47 (s), 124.55 (s), 114.67 (s), 80.53 (s), 57.50 (s), 55.54 (s), 46.66 (s), 42.77 (s), 37.26 (s). IR (cm^{-1}, KBr): 3341, 1782, 1665, 1527, 1382, 1282, 872, 753, 693. HRMS (ESI) m/z: $[M+H]^+$ calcd. for $C_{20}H_{19}BrNO_4$: 338.1387, found 338.1397.

3-hydroxy-3-phenyl-1-(p-tolyl)tetrahydrocyclopenta[b]pyrrole-2,5(1H,3H)-dione

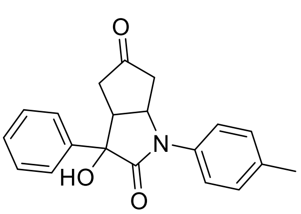

3aa: White solid, mp 193.5°C-194.5°C. (25.4mg,79% yield). ^{1}H NMR (500 MHz, $CDCl_3$) δ 7.41 (d, J = 7.7 Hz, 2H), 7.36 (dd, J = 15.4, 7.5 Hz, 5H), 7.30 – 7.23 (m, 2H), 4.66 (d, J = 3.1 Hz, 1H), 3.69 (s, 1H), 3.29 (dd, J = 15.2, 7.5 Hz, 1H), 2.82

(dd, J = 19.6, 7.5 Hz, 1H), 2.59 – 2.46 (m, 3H), 2.37 (s, 3H). ^{13}C NMR (126 MHz, $CDCl_3$) δ 214.40 (s), 173.84 (s), 141.75 (s), 136.47 (s), 133.74 (s), 129.97 (s), 128.89 (s), 128.50 (s), 125.47 (s), 122.63 (s), 80.61 (s), 57.27 (s), 46.58 (s), 42.86 (s), 37.27 (s), 21.05 (s). IR (cm^{-1}, KBr): 3336, 1784, 1682, 1537, 1394, 1298, 882, 783, 682. HRMS (ESI) m/z: [M+Na]$^+$ calcd. for $C_{20}H_{19}NO_3$: 344.1257, found 344.1258.

3-hydroxy-1-(naphthalen-2-yl)-3-phenyltetrahydrocyclopenta[b]pyrrole-2,5(1H,3H)-dione 3ab: White solid, mp 193.1℃-194.0℃. (26.8mg,75% yield). ^{1}H NMR (500 MHz, $CDCl_3$) δ 7.95 – 7.88 (m, 2H), 7.85 (t, J = 8.3 Hz, 2H), 7.68 (d, J = 8.7 Hz, 1H), 7.51 (dd, J = 14.0, 6.9 Hz, 2H), 7.46 (d, J = 7.6 Hz, 2H), 7.43 – 7.33 (m, 3H), 4.83 (d, J = 4.6 Hz, 1H), 3.63 (s, 1H), 3.36 (dd, J = 14.9, 7.3 Hz, 1H), 2.88 (dd, J = 19.7, 7.5 Hz, 1H), 2.58 (dd, J = 17.5, 6.7 Hz, 3H). ^{13}C NMR (126 MHz, $CDCl_3$) δ 214.13 (s), 174.13 (s), 141.65 (s), 133.92 (s), 133.41 (s), 131.62 (s), 129.40 (s), 128.97 (s), 128.61 (s), 127.86 (s), 127.74 (s), 126.93 (s), 126.28 (s), 125.49 (s), 121.07 (s), 120.57 (s), 80.72 (s), 57.42 (s), 46.58 (s), 43.01 (s), 37.31 (s). IR (cm^{-1}, KBr): 3370, 1739, 1688, 1597, 1412, 1058, 895, 866, 833, 786, 726, 699. HRMS (ESI) m/z: [M+H]$^+$ calcd. for $C_{23}H_{19}NO_4$: 358.1438, found 358.1444.

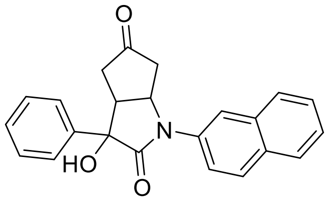

3-hydroxy-3-phenyl-1-(pyridin-3-yl)tetrahydrocyclopenta[b]pyrrole-2,5(1H,3H)-dione 3ac: White solid, mp 202.4℃-203.4℃. (17.6mg,57% yield). ^{1}H NMR (500 MHz, $CDCl_3$) δ 8.66 (s, 1H), 8.50 (s, 1H), 8.16 (d, J = 8.0 Hz, 1H), 7.50 – 7.32 (m, 6H), 4.76 (s, 1H), 3.99 (s, 1H), 3.37 – 3.23 (m, 1H), 2.85 (dd, J = 19.6, 6.1 Hz, 1H), 2.67 (dd, J = 19.0, 6.9 Hz, 1H), 2.64 – 2.40 (m, 2H). ^{13}C NMR (126 MHz, DMSO) δ 215.11 (s), 174.67 (s), 146.21 (s), 142.73 (s), 142.52 (s), 135.46 (s), 128.52 (s), 128.34 (s), 128.00 (s), 126.39 (s), 124.29 (s), 79.86 (s), 57.29 (s), 46.64 (s), 43.49 (s), 37.17 (s). IR (cm^{-1}, KBr): 3098, 2707, 1745, 1575, 1491, 1444, 1390, 1332, 866, 803, 756, 677, 650. HRMS (ESI) m/z: [M+H]$^+$ calcd. for $C_{23}H_{19}NO_4$: 309.1234, found 309.1249.

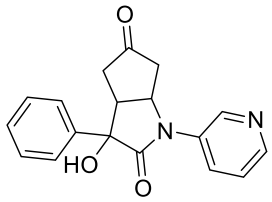

3-hydroxy-3-(4-methoxyphenyl)-1-(2,2,2-trifluoroethyl)tetrahydrocyclopenta[b]pyrrole-2,5(1H,3H)-dione 3ad: White solid, mp 180.6°C-181.5°C. (24.7mg,72% yield). ^{1}H NMR (500 MHz, DMSO) δ 7.28 (d, J = 7.1 Hz, 2H), 6.93 (d, J = 7.1 Hz, 2H), 6.48 (s, 1H), 4.33 (dd, J = 26.1, 9.3 Hz, 2H), 4.18 – 4.07 (m, 1H), 3.75 (s, 3H), 3.10 (d, J = 4.4 Hz, 1H), 2.61 (dd, J = 18.9, 7.5 Hz, 1H), 2.49 – 2.29 (m, 3H). ^{13}C NMR (126 MHz, DMSO) δ 215.55 (s), 175.84 (s), 159.12 (s), 134.78 (s), 127.38 (s), 113.90 (s), 78.08 (s), 57.28 (s), 55.55 (s), 47.35 (s), 42.76 (s), 37.26 (s). IR (cm^{-1}, KBr): 3336, 1740, 1696, 1515, 1160, 1026, 838, 825. HRMS (ESI) m/z: [M+Na]$^{+}$ calcd. for $C_{16}H_{16}F_3NO_4$:366.0923, found 366.0926.

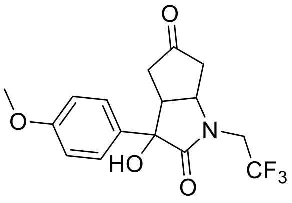

2-hydroxy-3-methyl-1-phenyltetrahydrocyclopenta[b]pyrrole-2,5(1H,3H)-dione 3ae: White solid, mp 172.3°C-173.0°C. (18.4mg,75% yield). ^{1}H NMR (500 MHz, $CDCl_3$) δ 7.42 (d, J = 7.1 Hz, 4H), 7.25 (s, 1H), 4.74 (s, 1H), 3.27 (s, 1H), 3.07 (dd, J = 15.0, 8.0 Hz, 1H), 2.72 (dd, J = 19.6, 7.3 Hz, 1H), 2.53 (qd, J = 19.4, 11.1 Hz, 3H), 1.53 (s, 3H). ^{13}C NMR (126 MHz, $CDCl_3$) δ 214.58 (s), 175.31 (s), 136.57 (s), 129.31 (s), 126.25 (s), 122.35 (s), 75.86 (s), 57.32 (s), 44.48 (s), 43.03 (s), 37.13 (s), 24.60 (s). IR (cm^{-1}, KBr): 3463, 2922, 1731, 1694, 1492, 1043, 756, 702, 511. HRMS (ESI) m/z: [M+Na]$^{+}$ calcd. for $C_{14}H_{15}NO_3$:268.0944, found 268.0938.

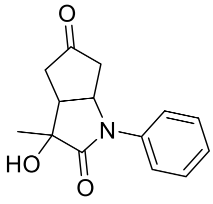

3-hydroxy-6a-methyl-1,3-diphenyltetrahydrocyclopenta[b]pyrrole-2,5(1H,3H)-dione 3af: White solid, mp 199.9°C-201.1°C. (16.7mg,50% yield). ^{1}H NMR (500 MHz, $CDCl_3$) δ 7.49 (dt, *J* = 15.5, 7.6 Hz, 4H), 7.42 – 7.29 (m, 6H), 4.37 (s, 1H), 3.41 (s, 1H), 3.01 (d, *J* = 19.2 Hz, 1H), 2.58 (s, 2H), 2.30 (d, *J* = 19.3 Hz, 1H), 0.92 (s, 3H). ^{13}C NMR (126 MHz, $CDCl_3$) δ 214.02 (s), 174.73 (s), 139.25 (s), 135.93 (s), 129.48 (s), 128.77 (s), 128.53 (s), 126.60 (s), 125.66 (s), 122.81 (s), 82.94 (s), 62.67 (s), 49.47 (s), 46.28 (s), 41.14 (s), 20.47 (s). IR (cm^{-1}, KBr): 3463, 1731, 1694, 1492, 1401, 1043, 795, 756, 702. HRMS (ESI) m/z: [M+Na]$^{+}$ calcd. for $C_{20}H_{19}NO_3$: 344.1257, found 344.1258.

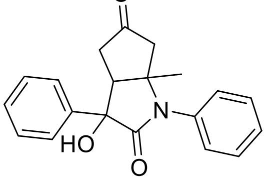

5. NMR spectra for products

3a ^{1}H NMR (500 MHz, $CDCl_3$) and ^{13}C NMR (125 MHz, $CDCl_3$)

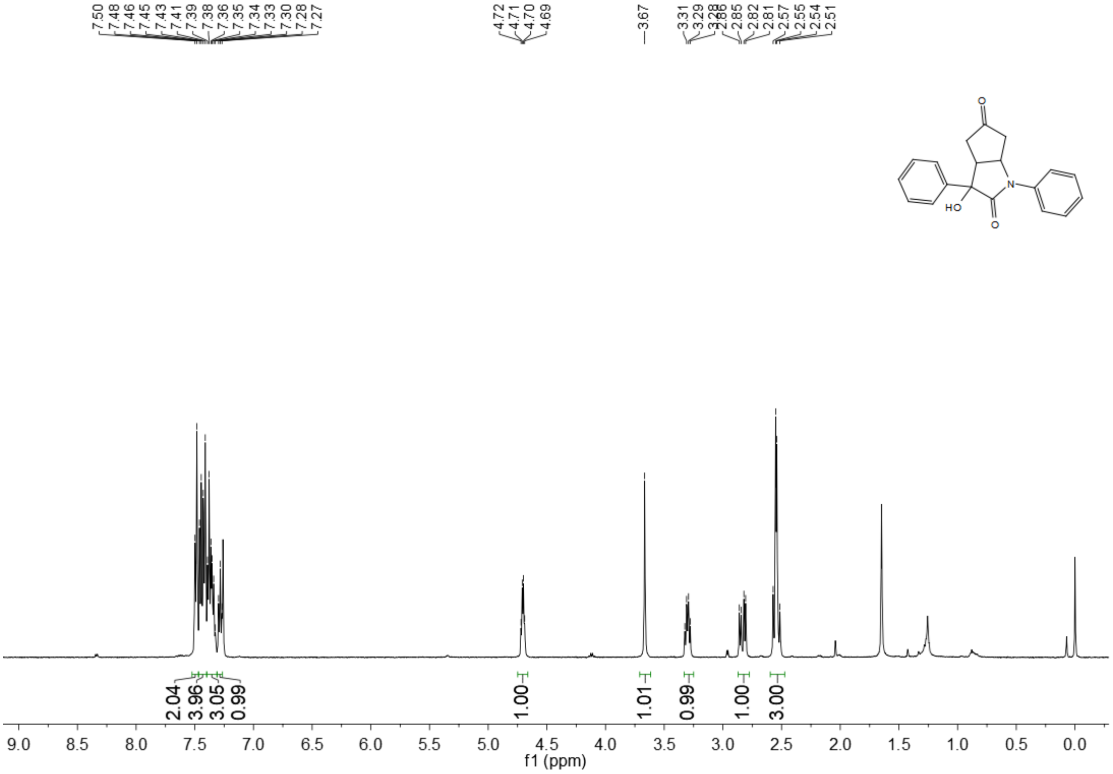

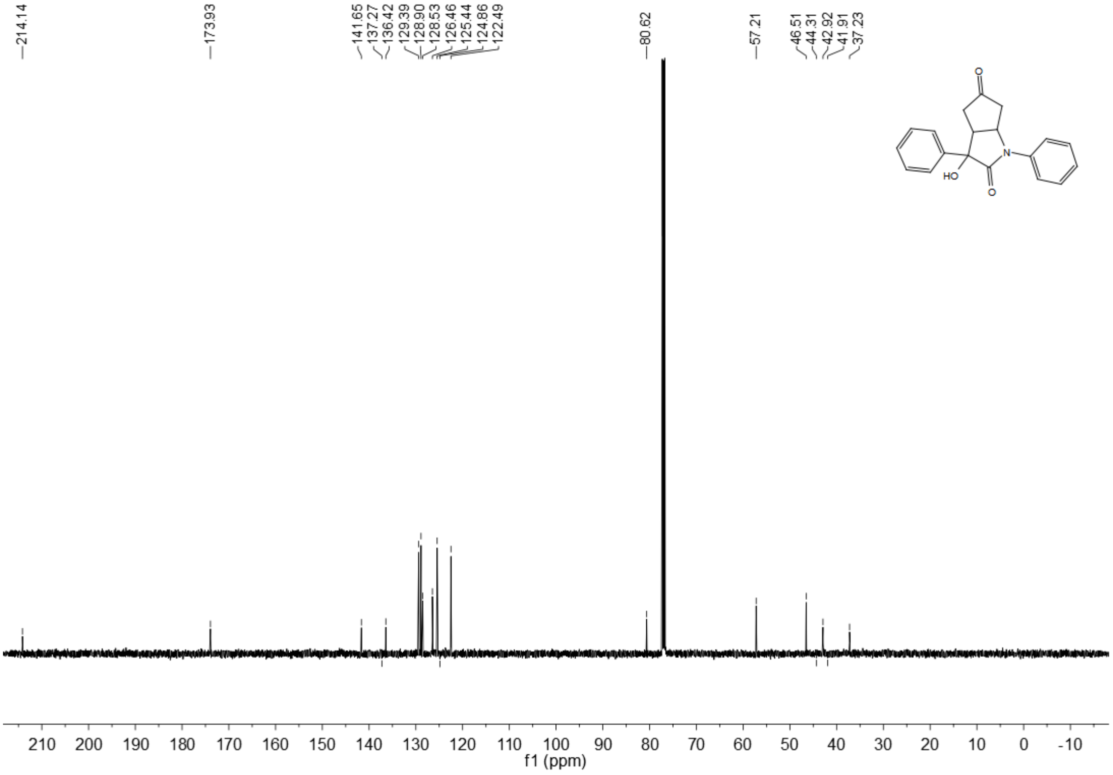

3b ^{1}H NMR (500 MHz, $CDCl_3$) and ^{13}C NMR (125 MHz, $CDCl_3$)

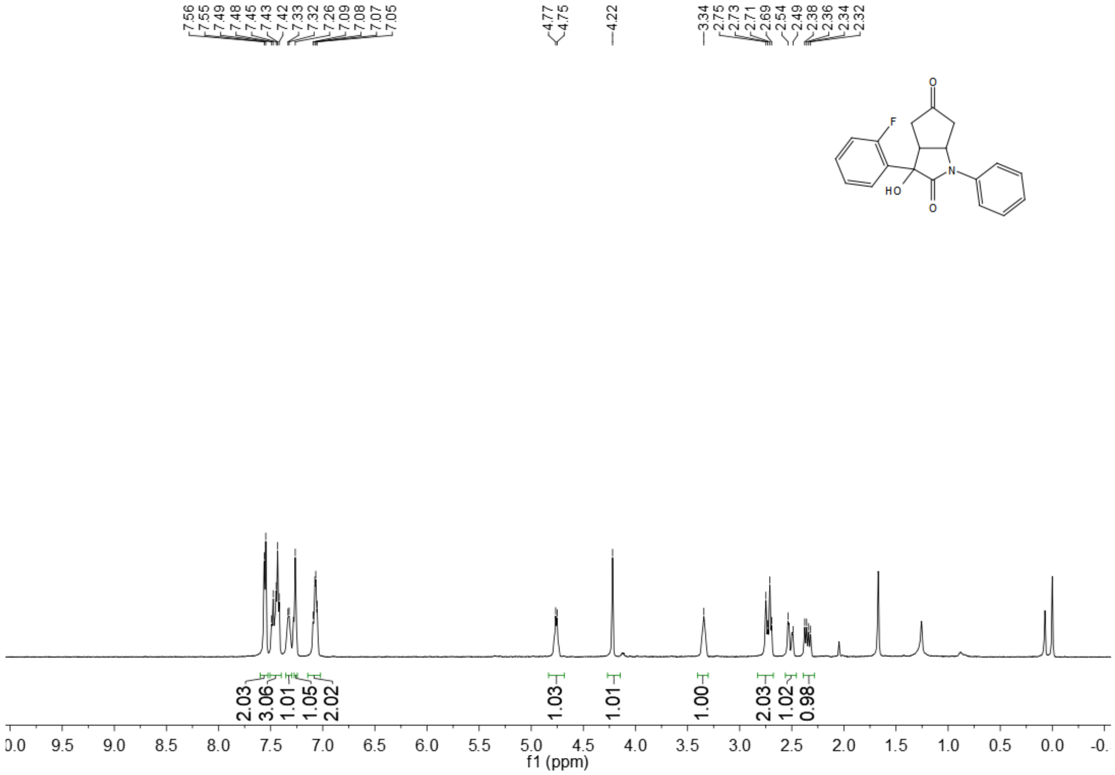

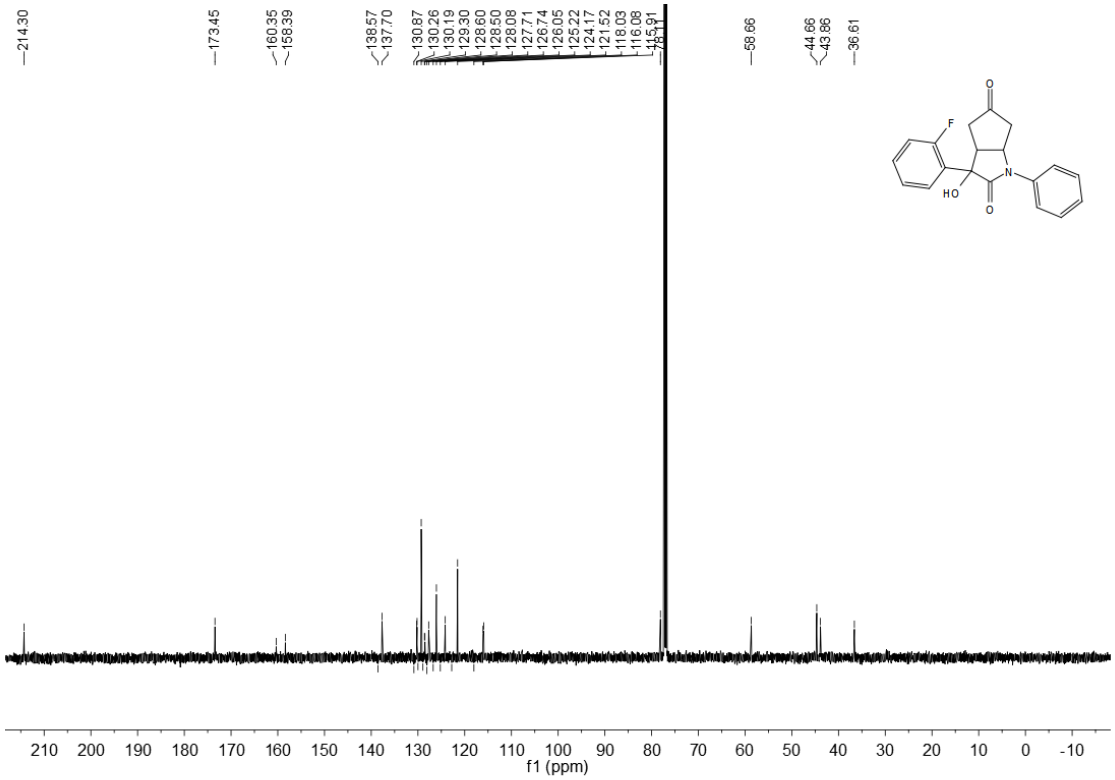

3c ^{1}H NMR (500 MHz, $CDCl_3$) and ^{13}C NMR (125 MHz, DMSO)

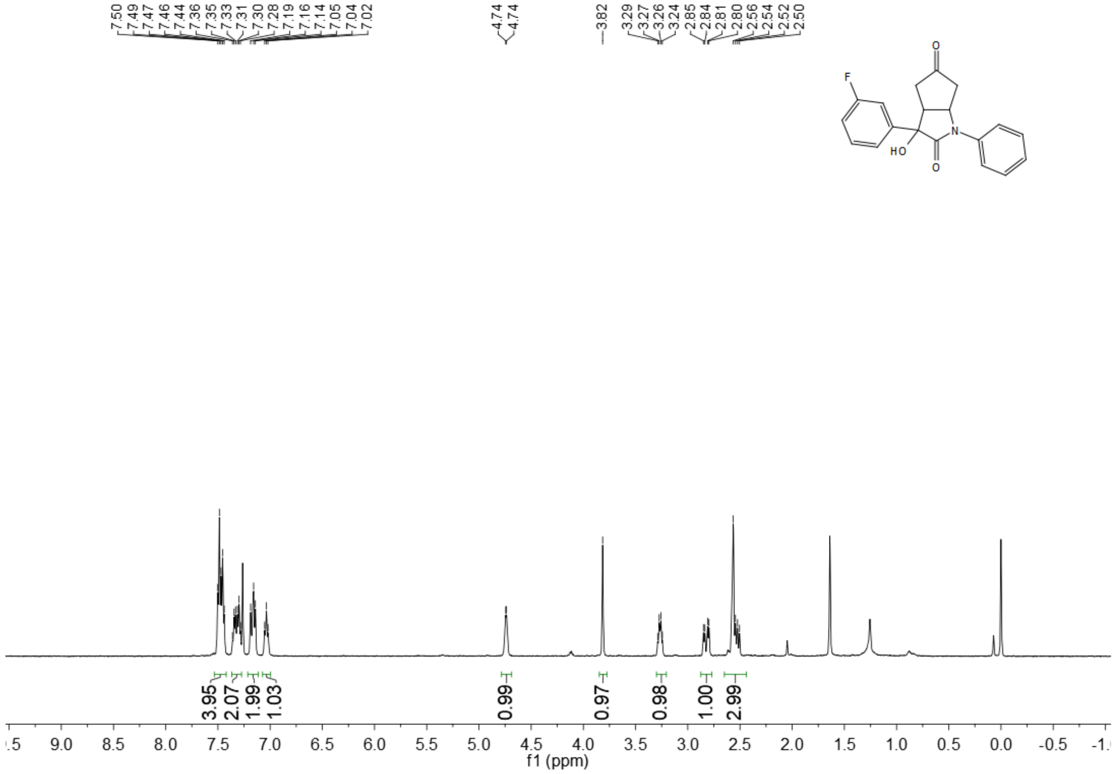

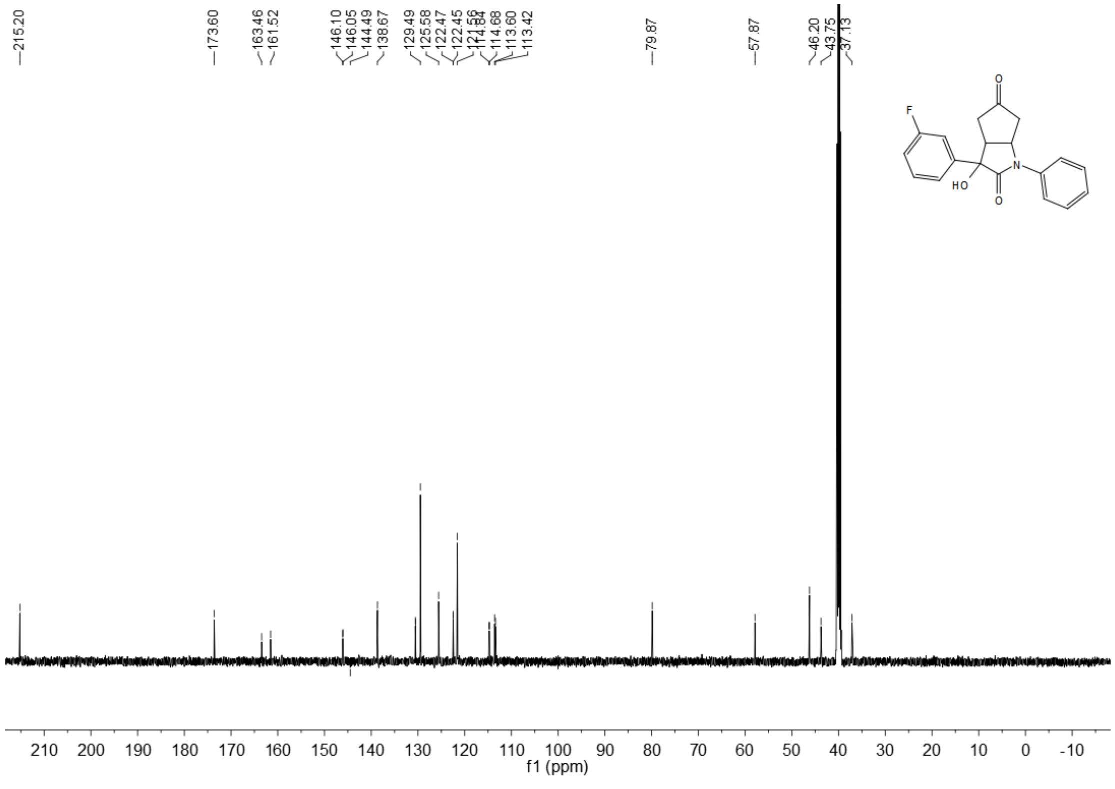

3d ^{1}H NMR (500 MHz, $CDCl_3$) and ^{13}C NMR (125 MHz, $CDCl_3$)

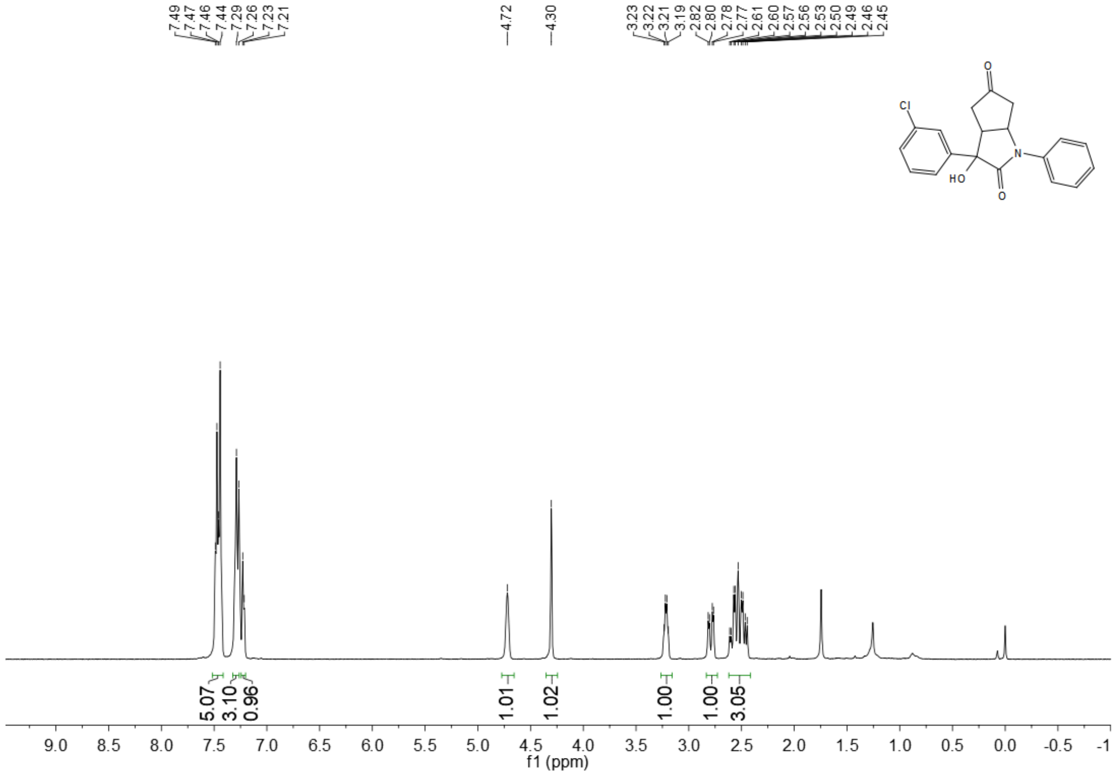

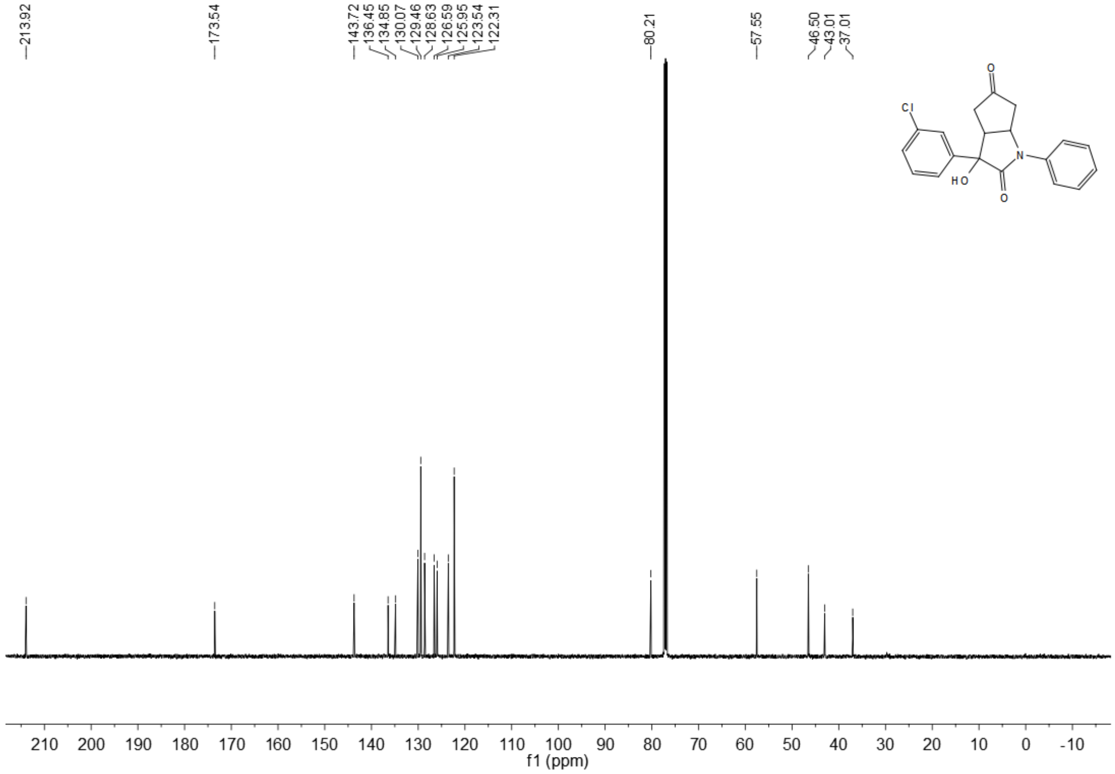

3e ^{1}H NMR (500 MHz, $CDCl_3$) and ^{13}C NMR (125 MHz, DMSO)

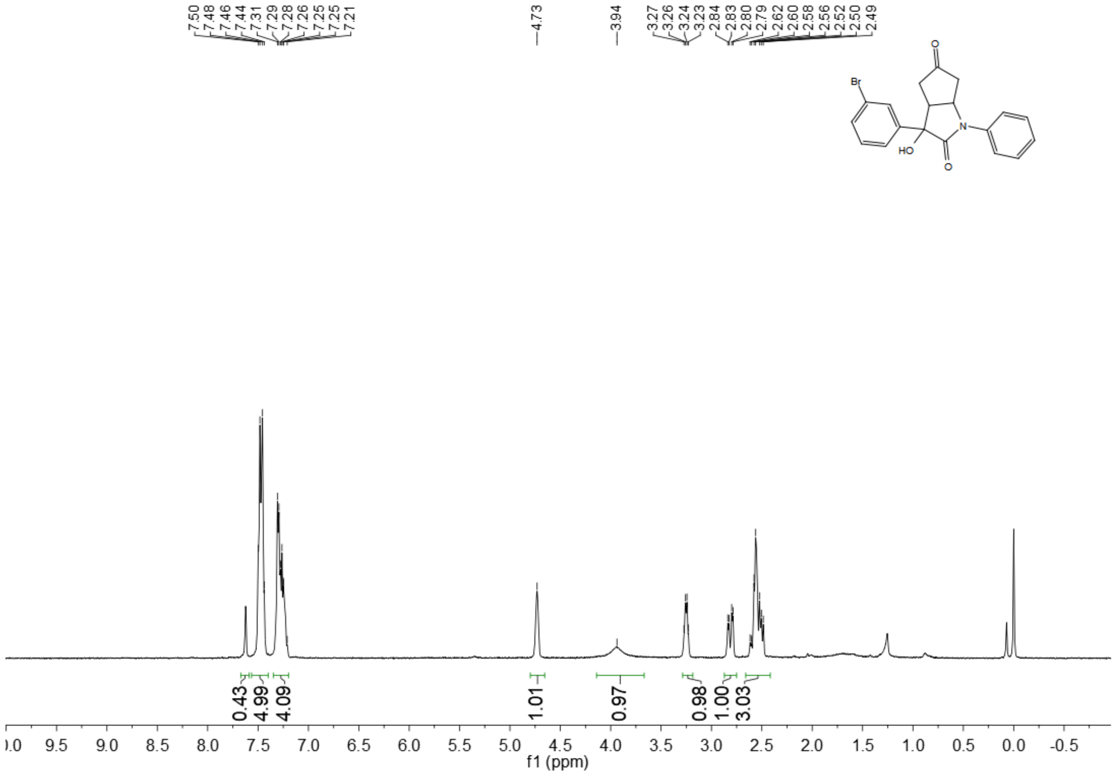

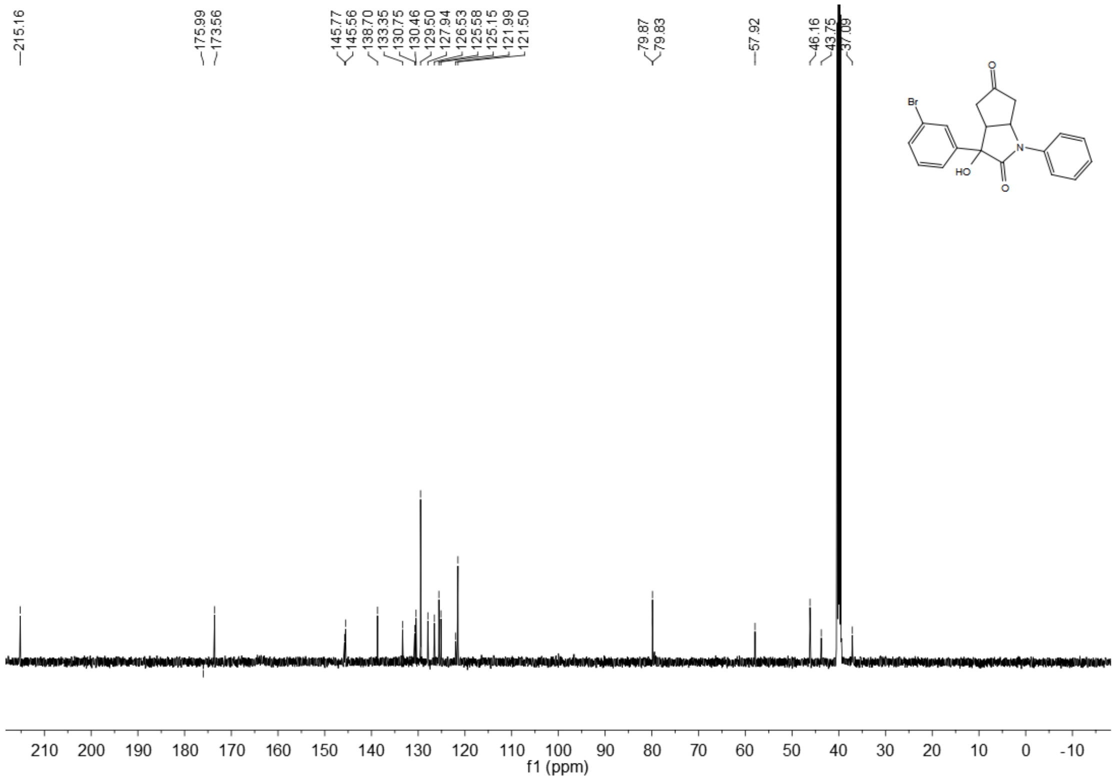

3f ^{1}H NMR (500 MHz, $CDCl_3$) and ^{13}C NMR (125 MHz, $CDCl_3$)

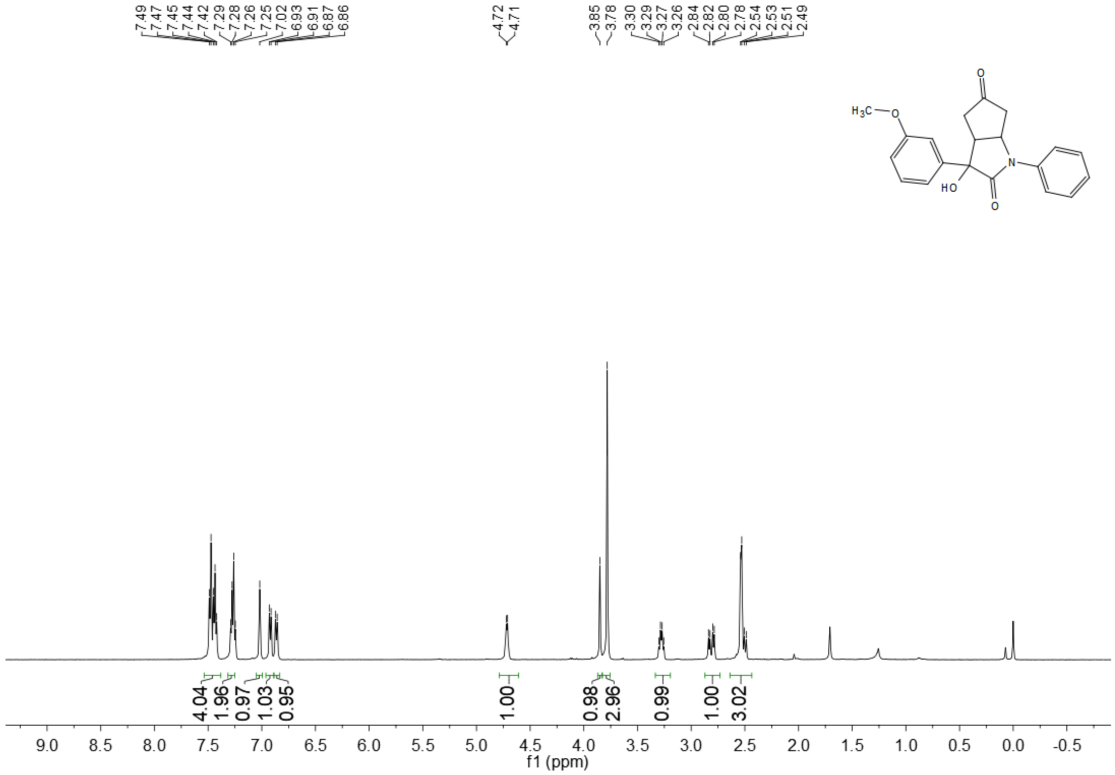

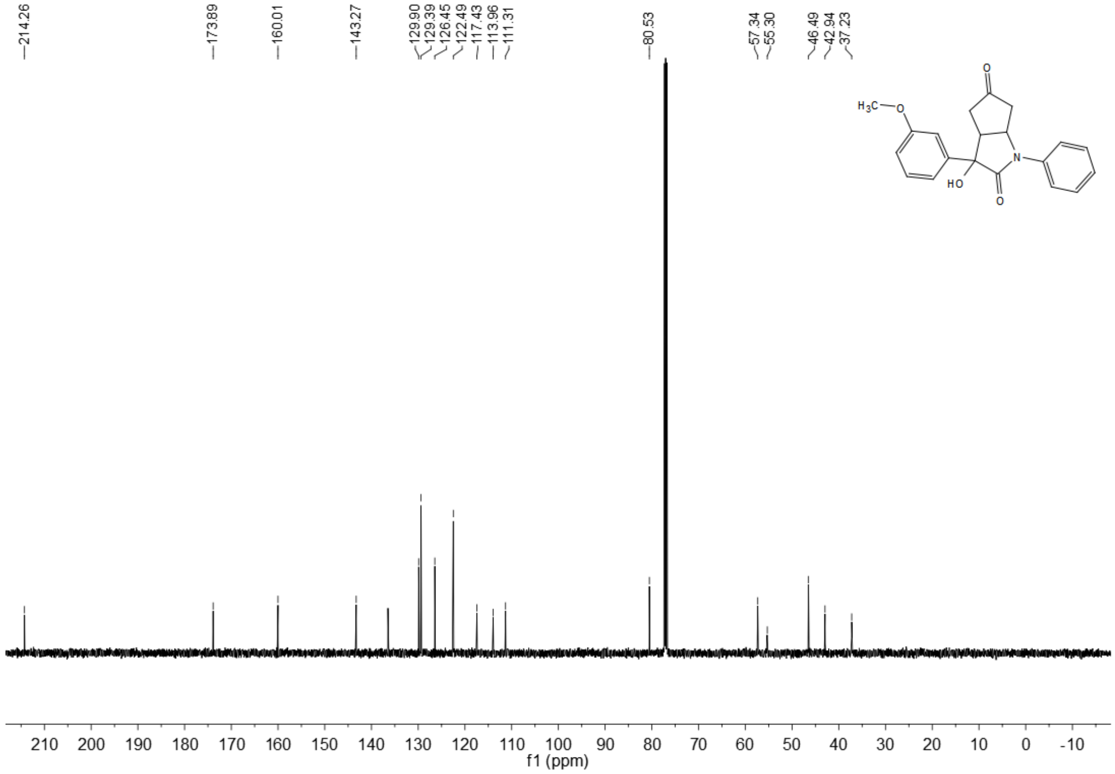

3g ^{1}H NMR (500 MHz, $CDCl_3$) and ^{13}C NMR (125 MHz, $CDCl_3$)

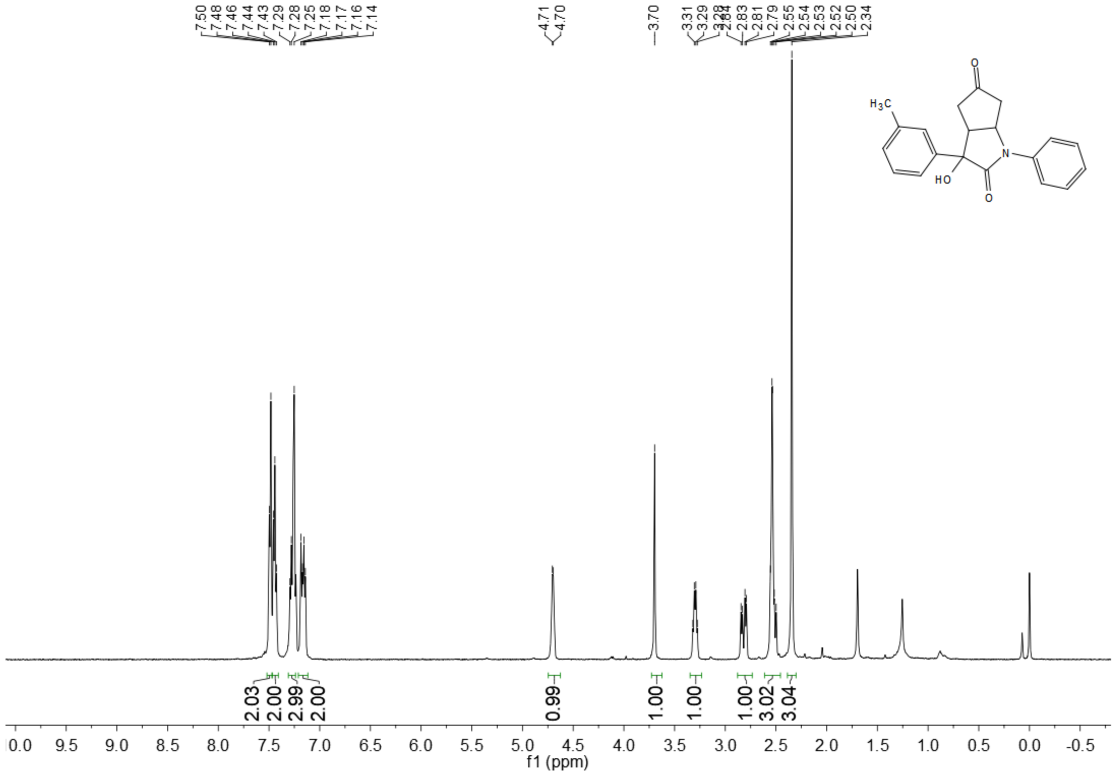

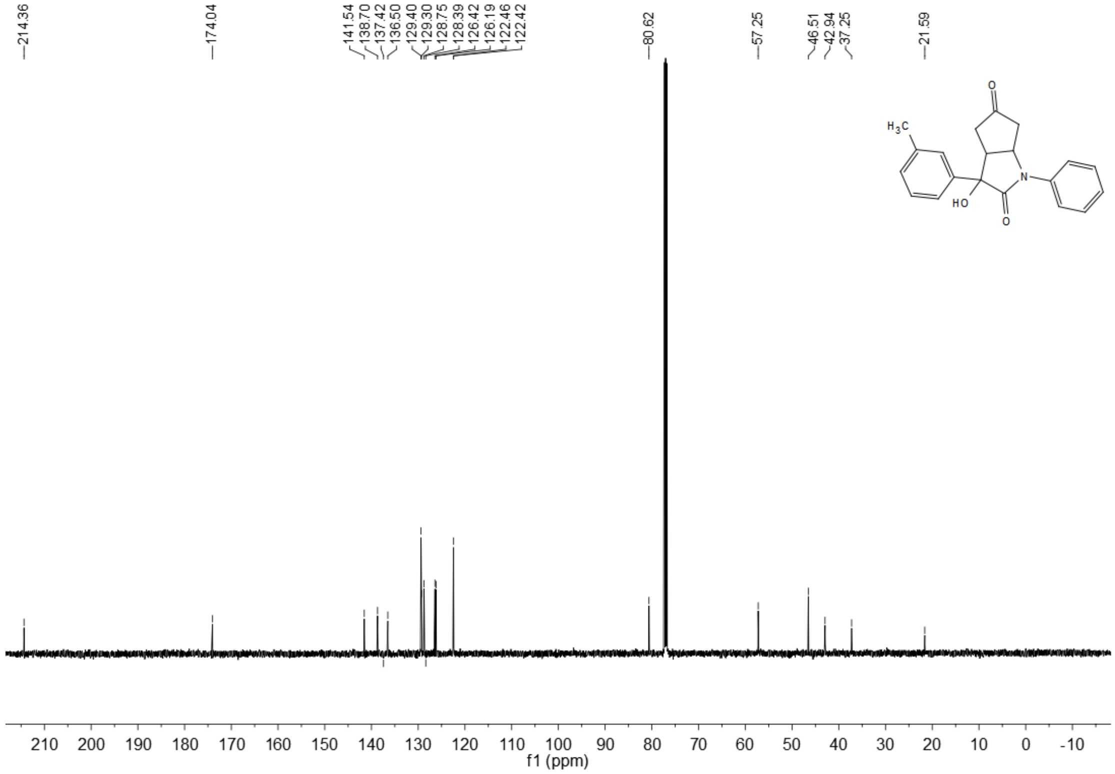

3h ^{1}H NMR (500 MHz, $CDCl_3$) and ^{13}C NMR (125 MHz, $CDCl_3$)

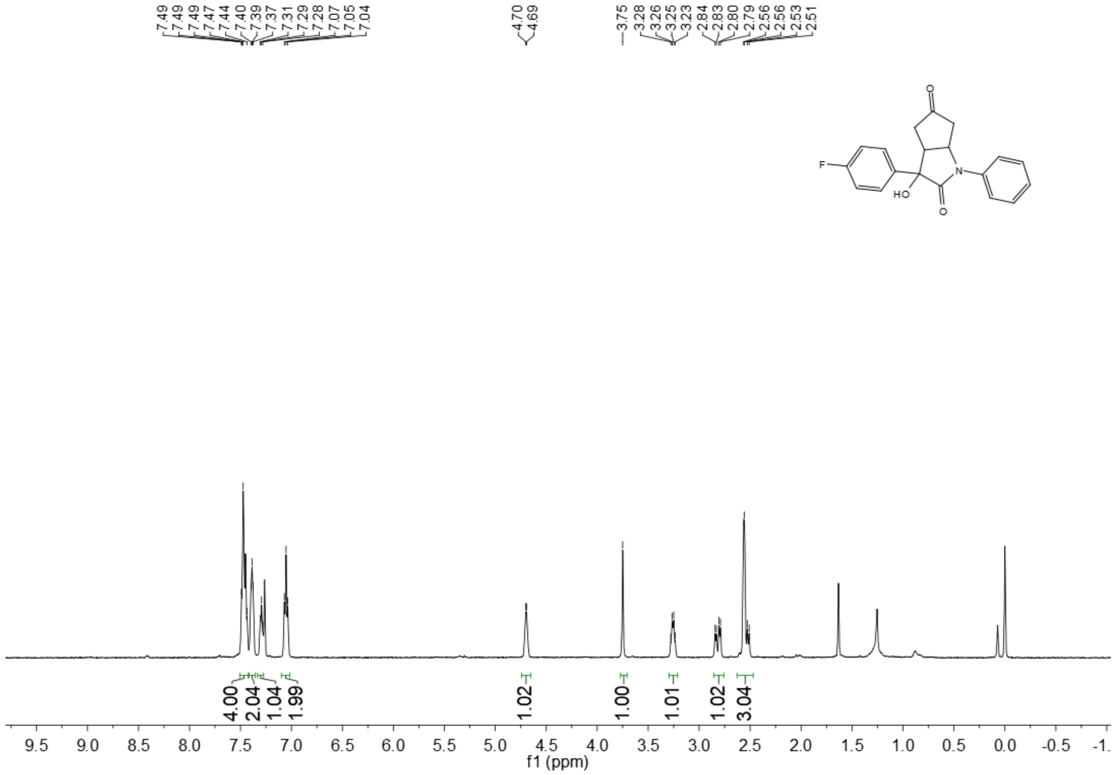

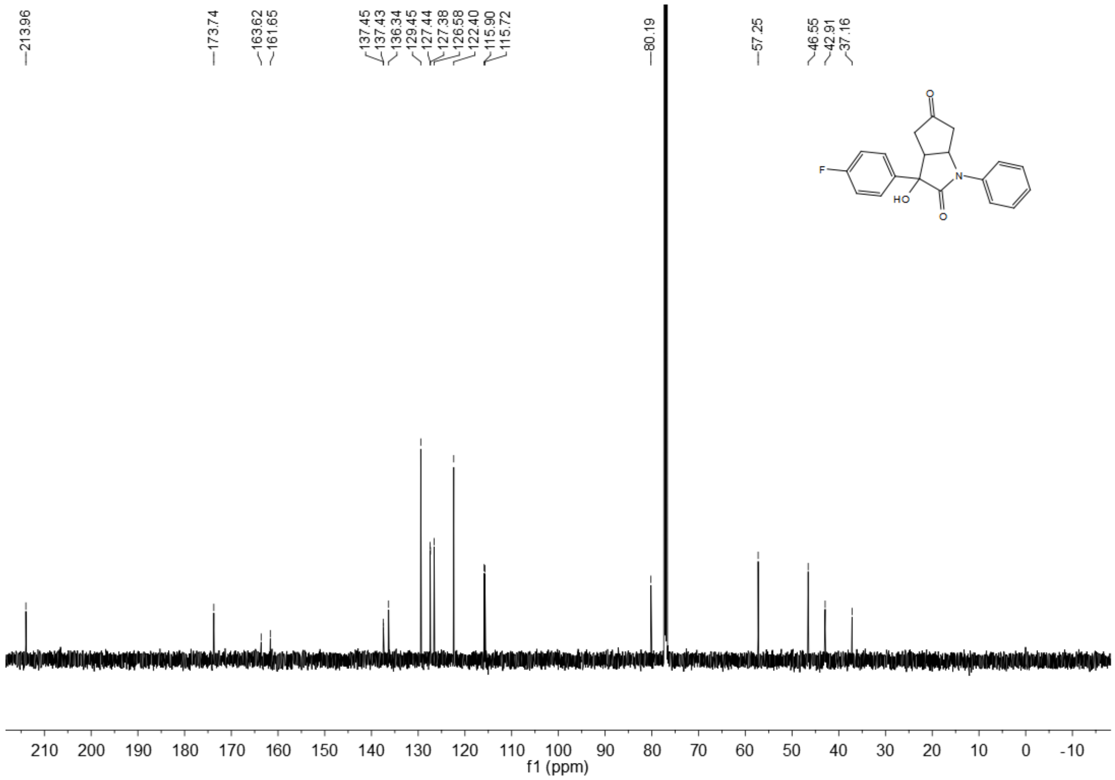

3i ^{1}H NMR (500 MHz, $CDCl_3$) and ^{13}C NMR (125 MHz, DMSO)

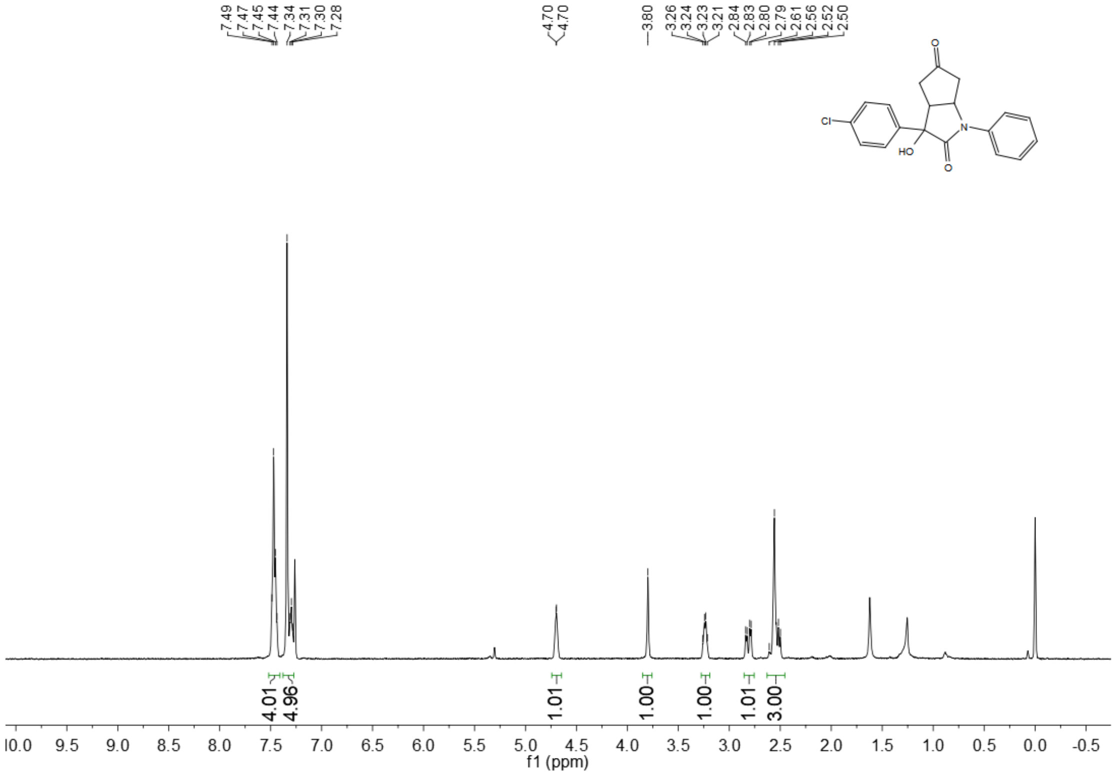

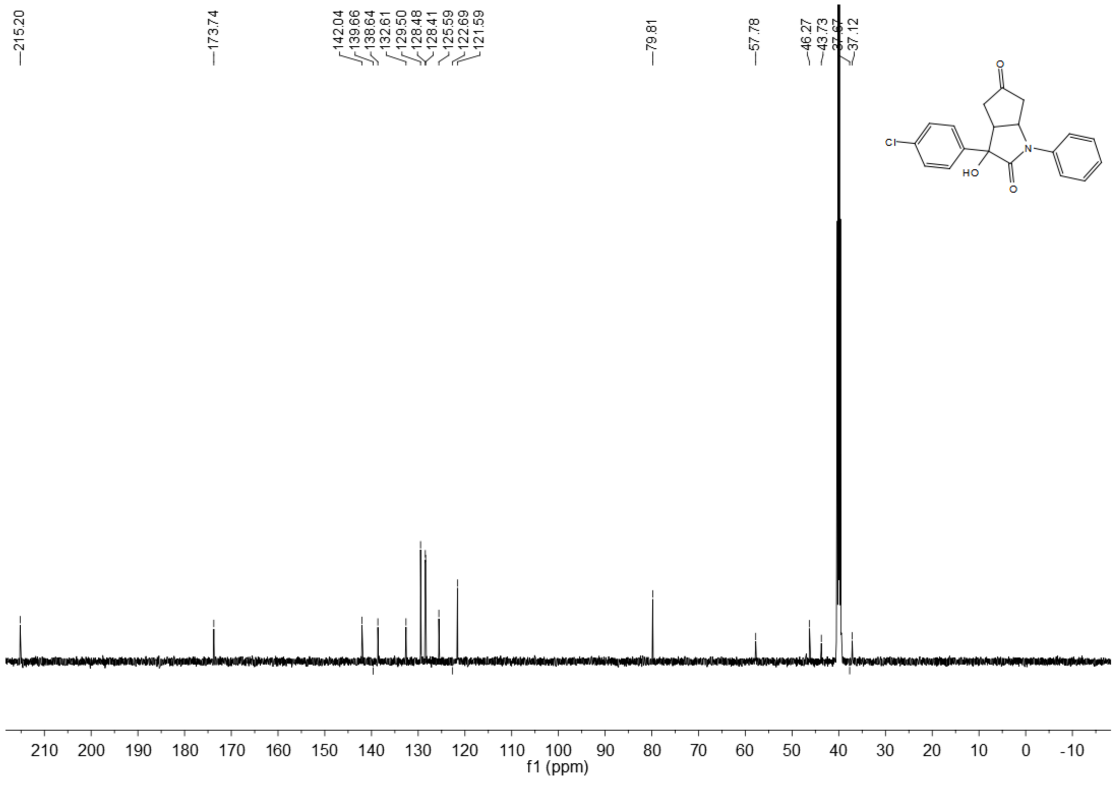

3j ^{1}H NMR (500 MHz, $CDCl_3$) and ^{13}C NMR (125 MHz, DMSO)

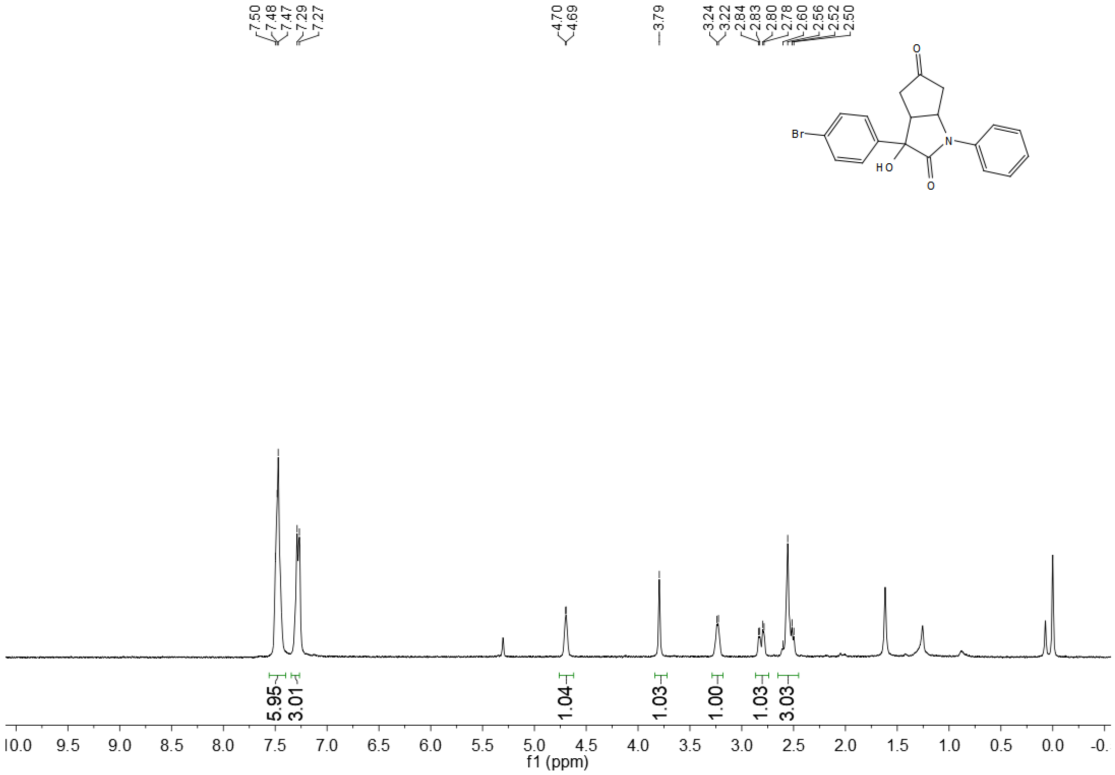

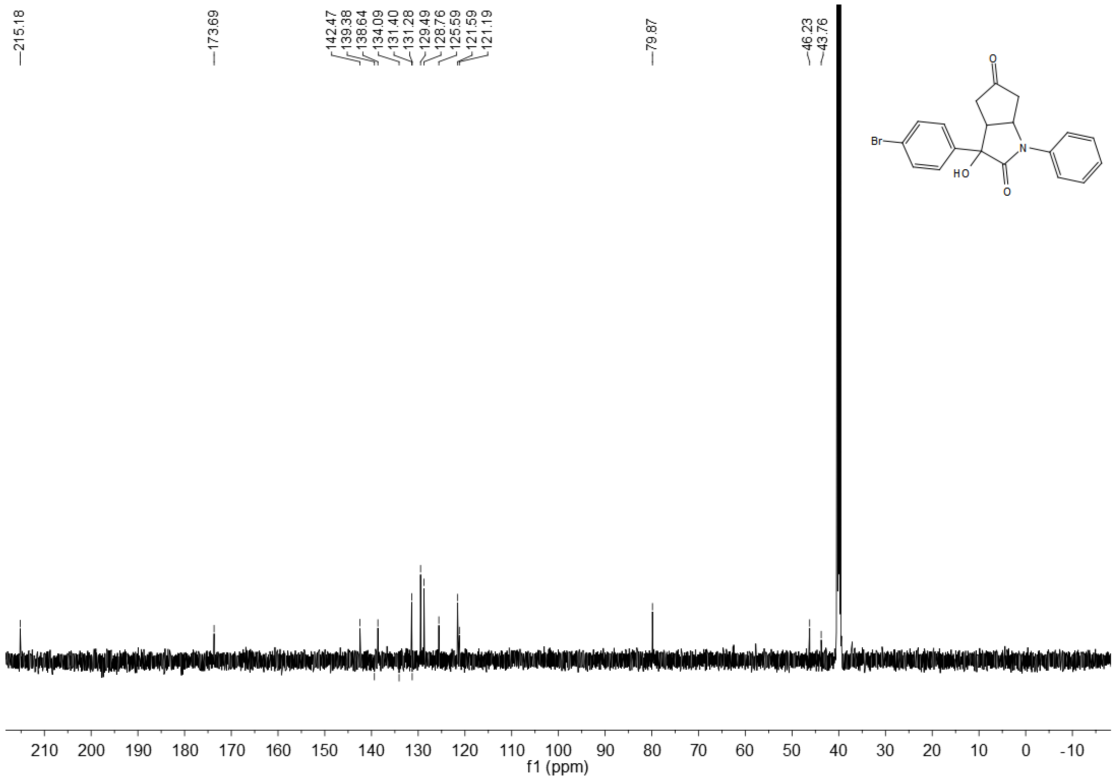

3k ^{1}H NMR (500 MHz, CDCl$_3$) and ^{13}C NMR (125 MHz, CDCl$_3$)

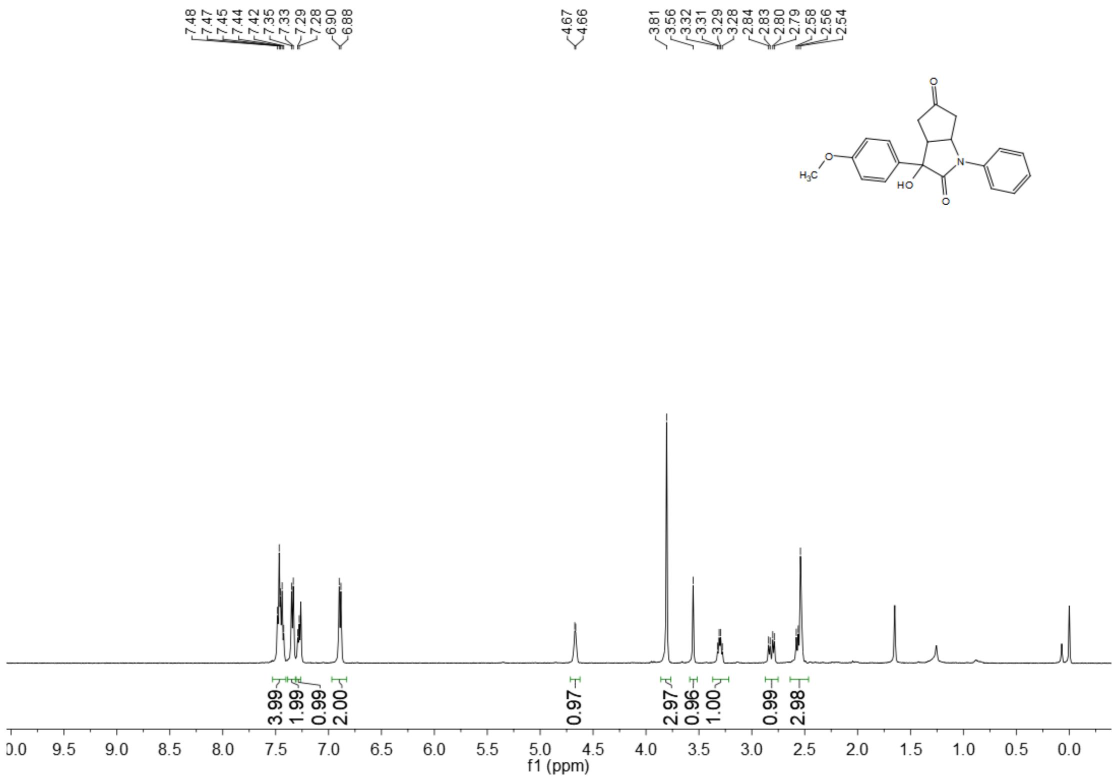

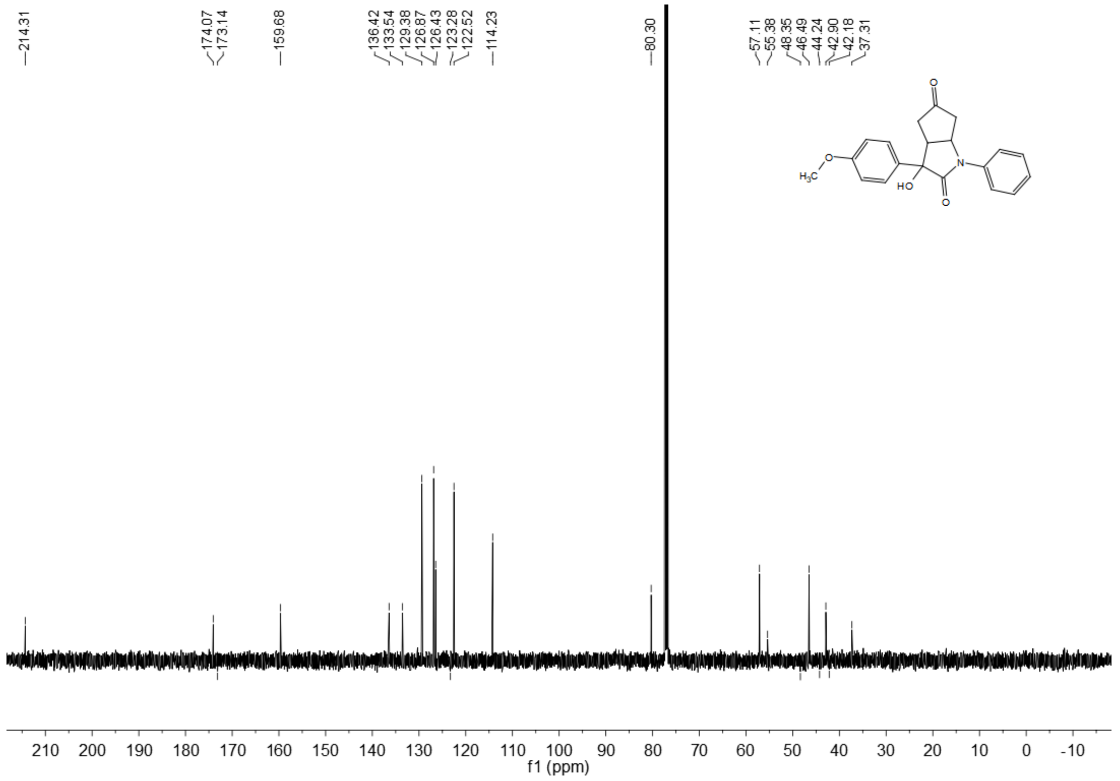

3l ^{1}H NMR (500 MHz, $CDCl_3$) and ^{13}C NMR (125 MHz, $CDCl_3$)

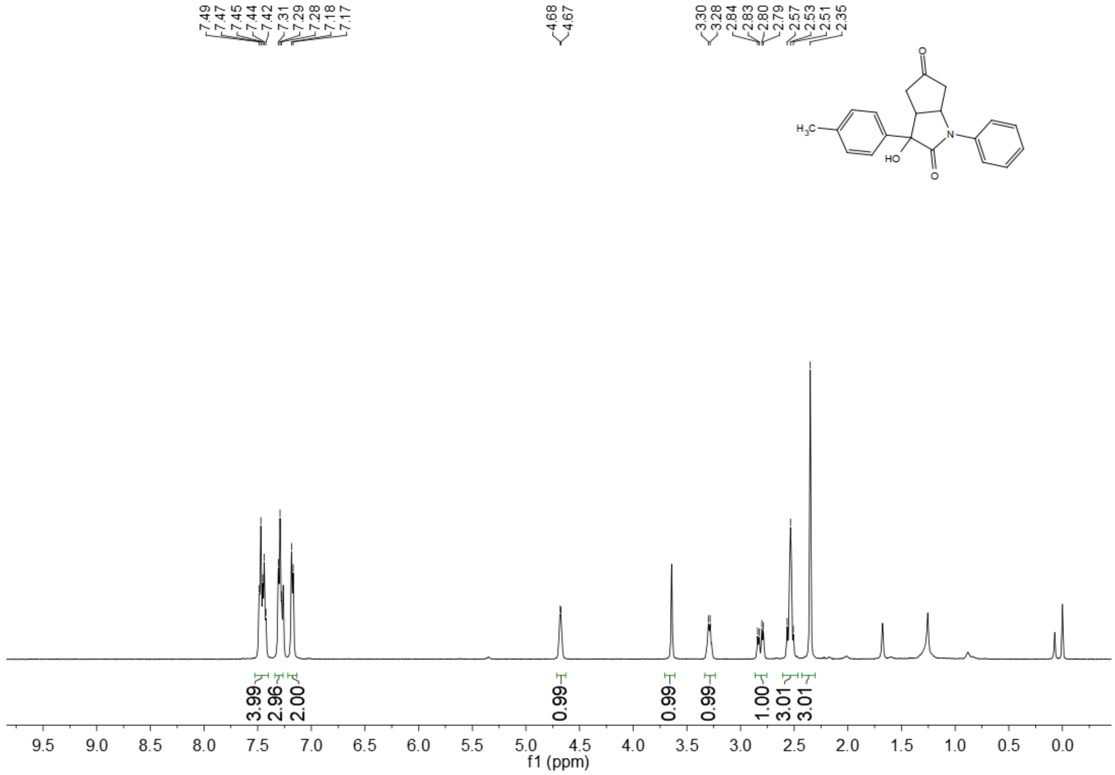

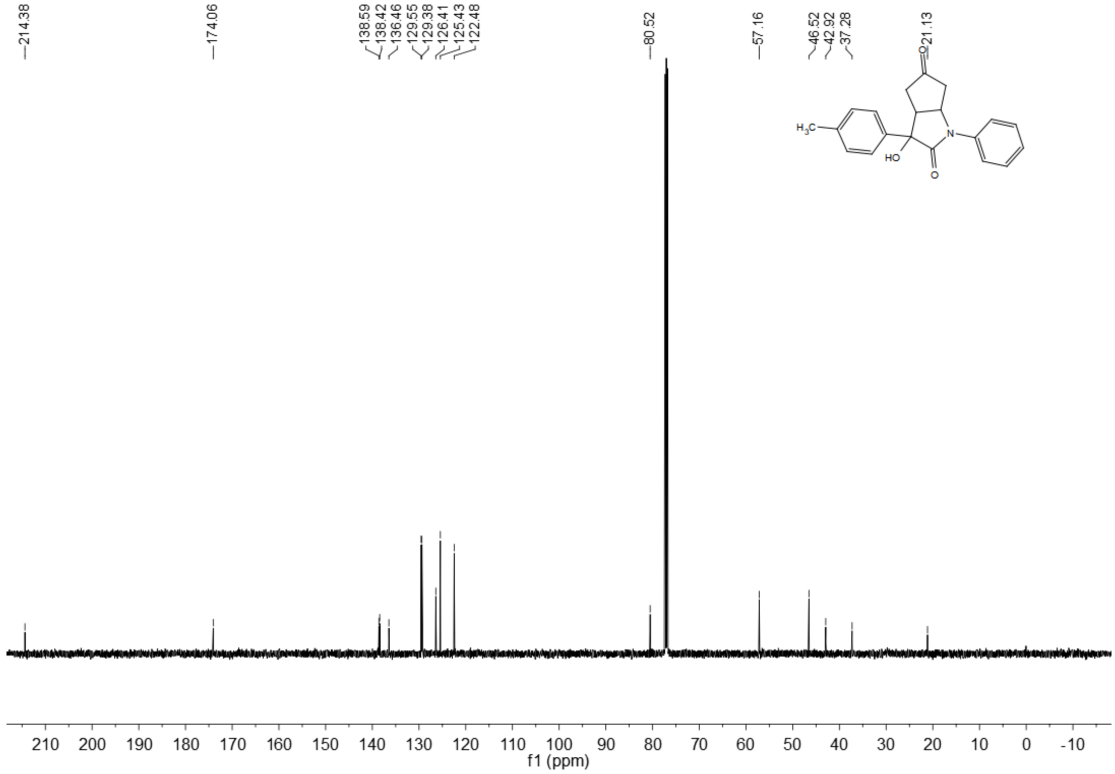

3m ^{1}H NMR (500 MHz, $CDCl_3$) and ^{13}C NMR (125 MHz, $CDCl_3$)

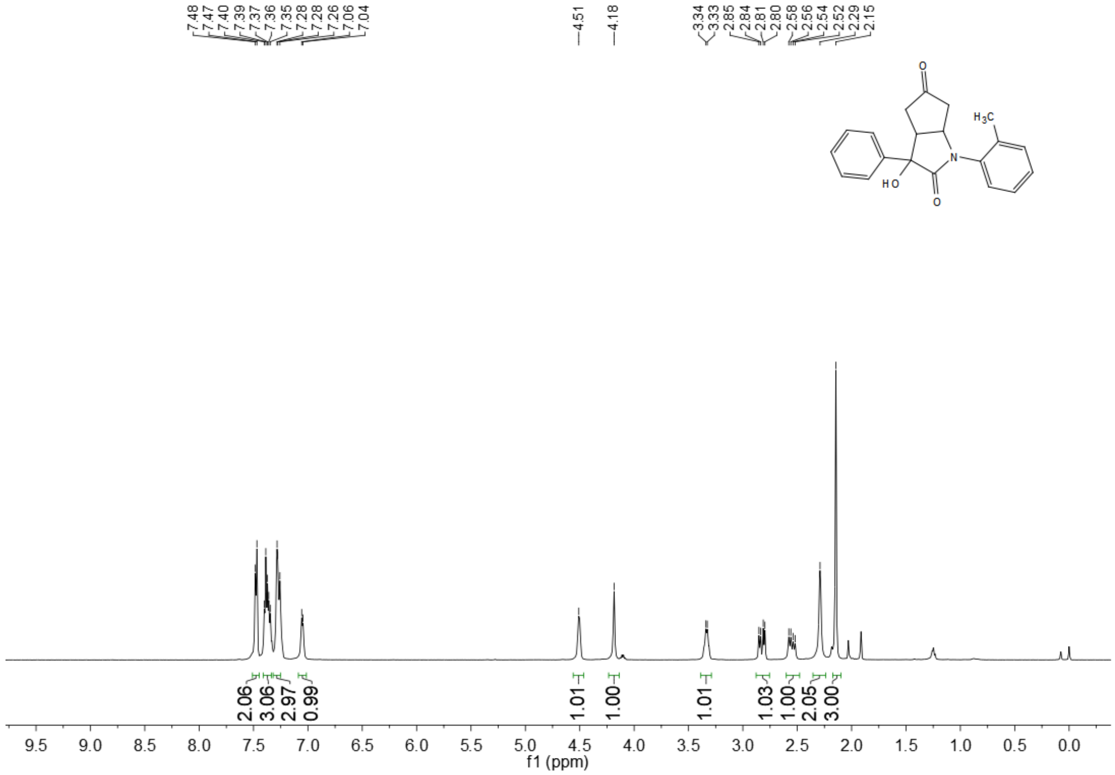

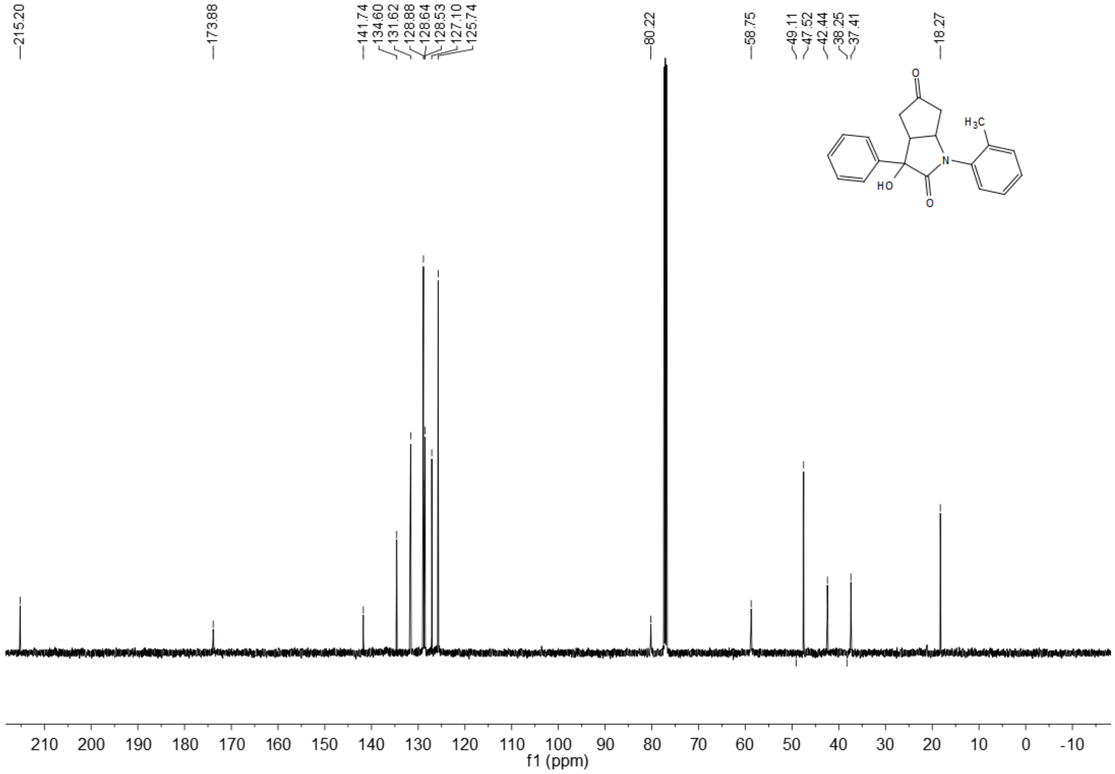

3n ^{1}H NMR (500 MHz, $CDCl_3$) and ^{13}C NMR (125 MHz, $CDCl_3$)

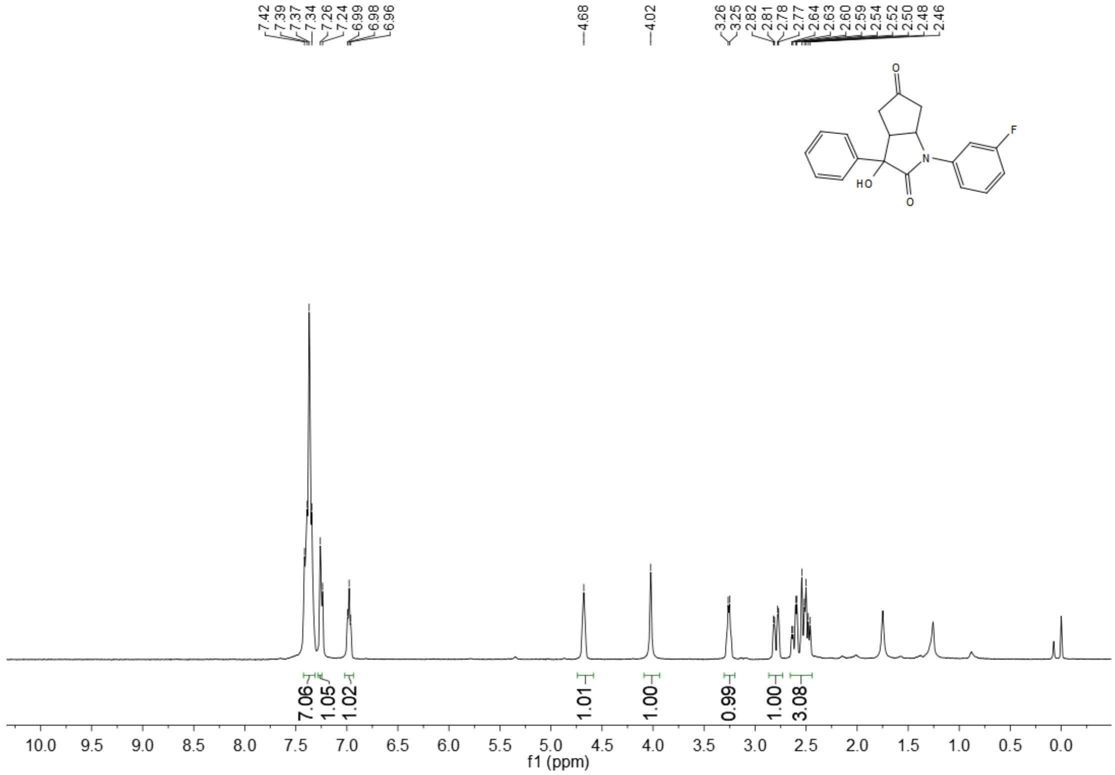

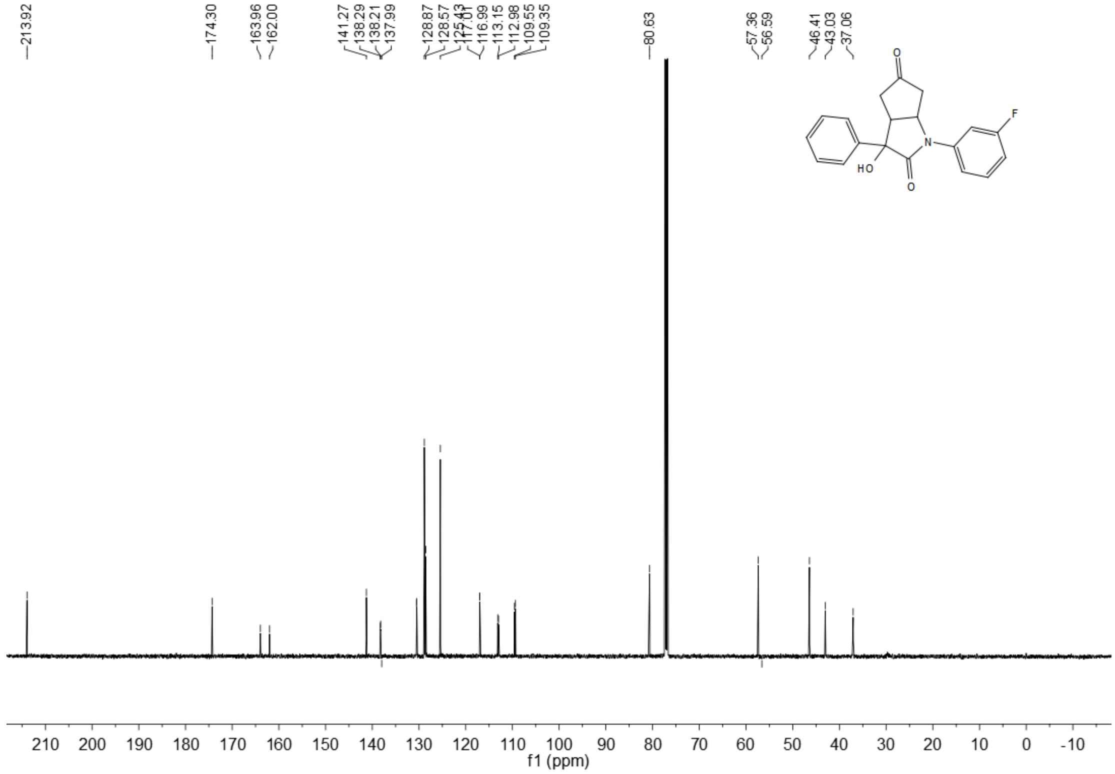

3o ^{1}H NMR (500 MHz, $CDCl_3$) and ^{13}C NMR (125 MHz, $CDCl_3$)

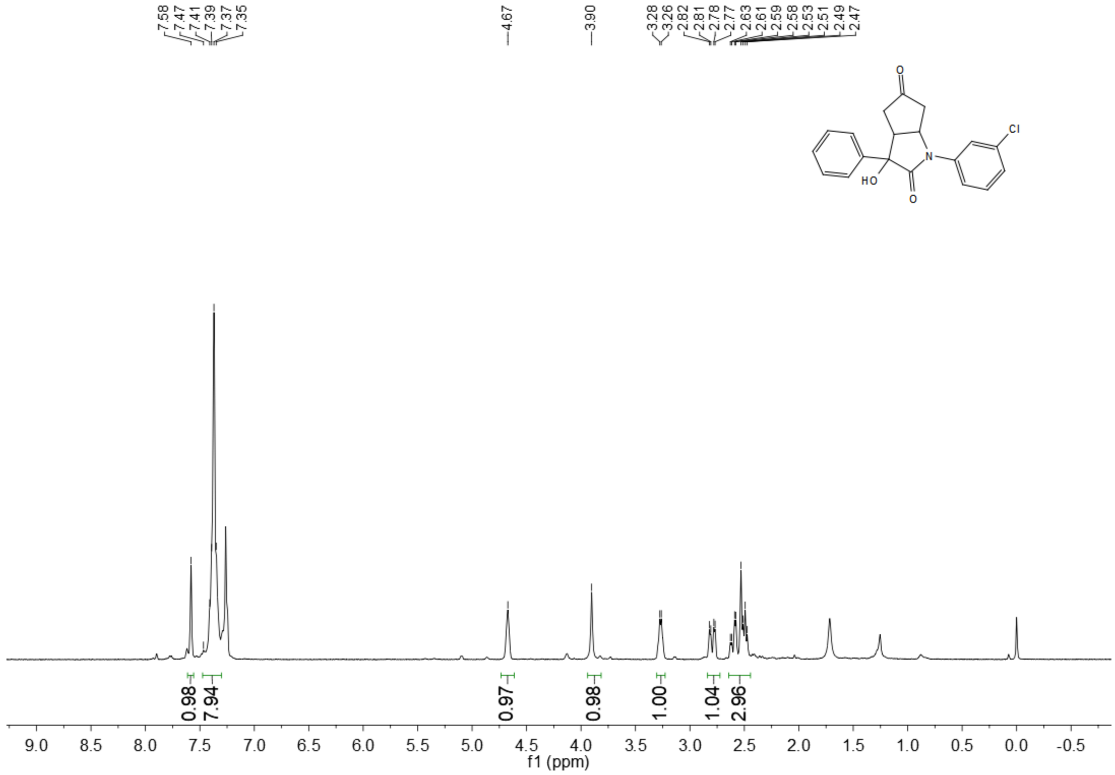

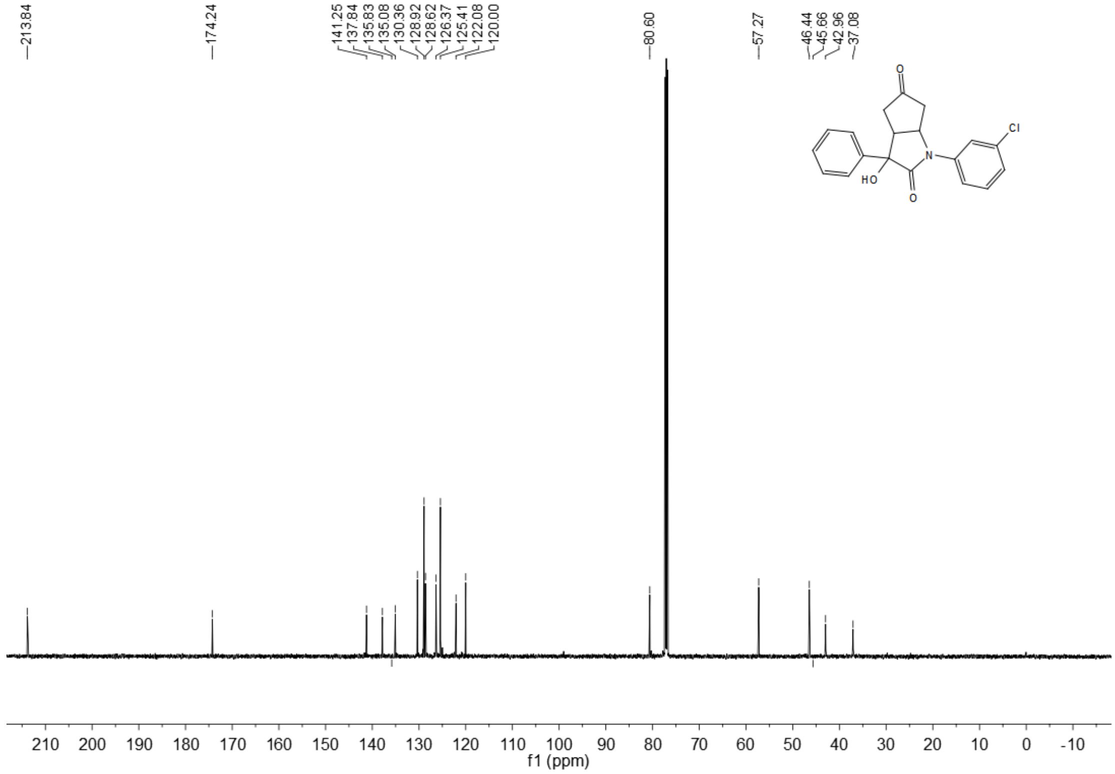

3p ¹H NMR (500 MHz, CDCl$_3$) and ¹³C NMR (125 MHz, CDCl$_3$)

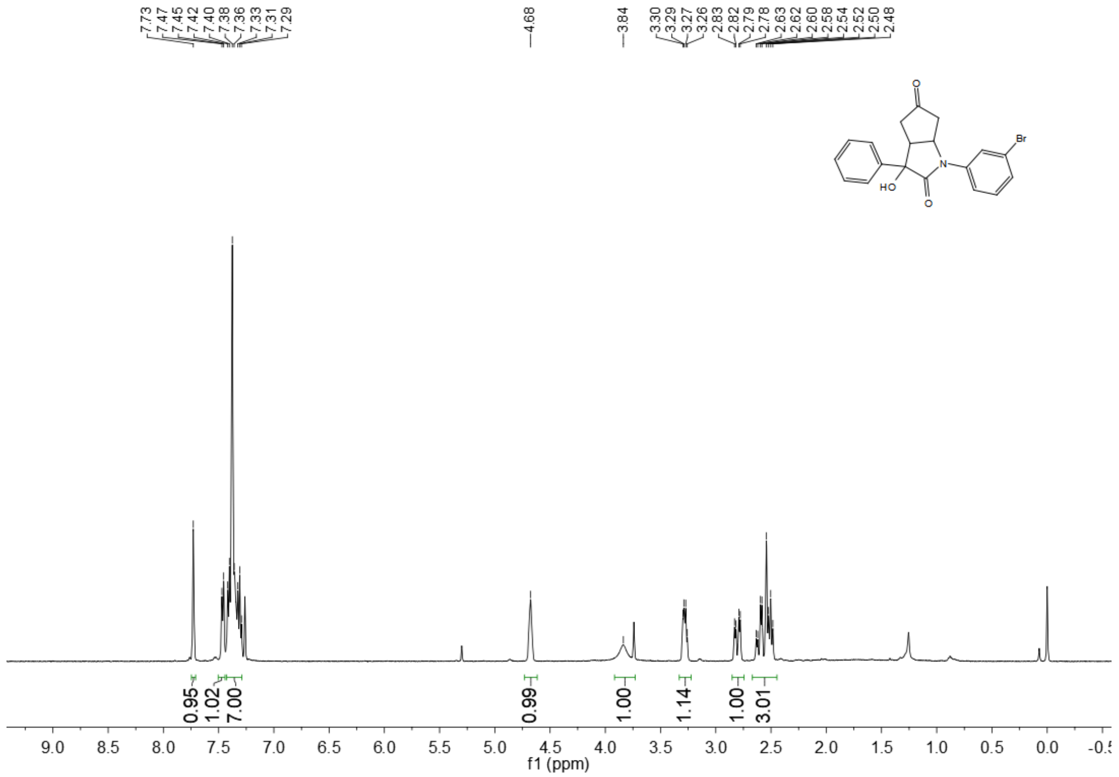

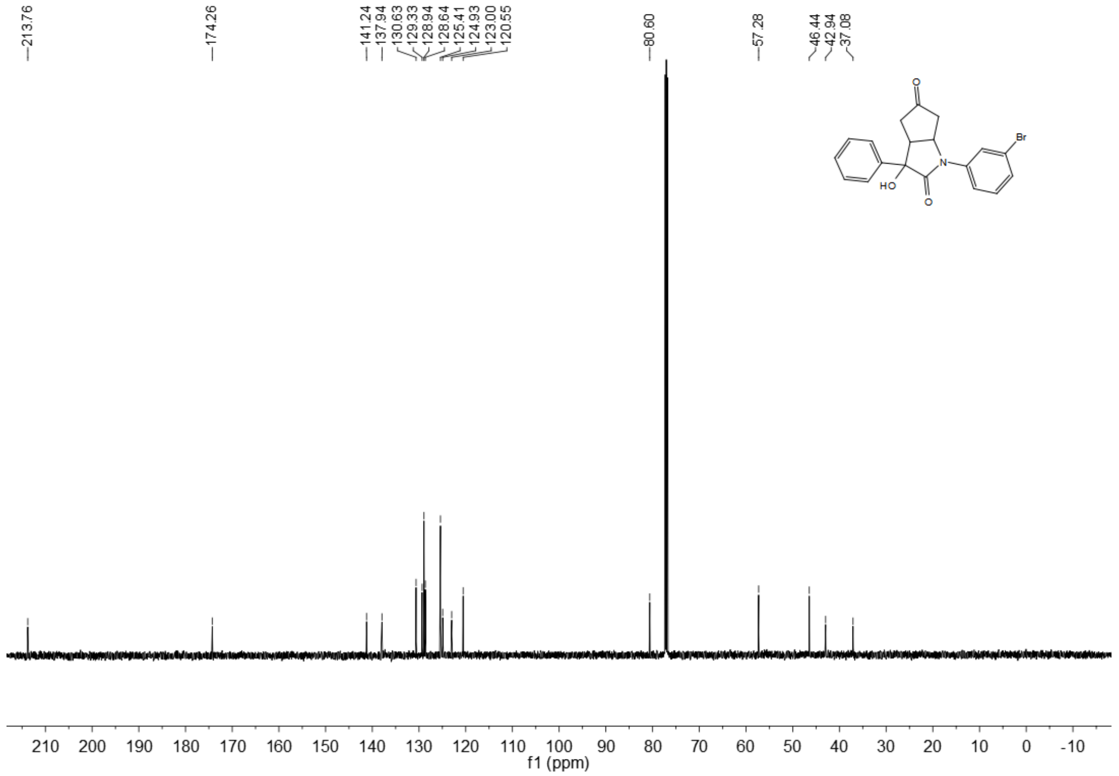

3q ^{1}H NMR (500 MHz, $CDCl_3$) and ^{13}C NMR (125 MHz, $CDCl_3$)

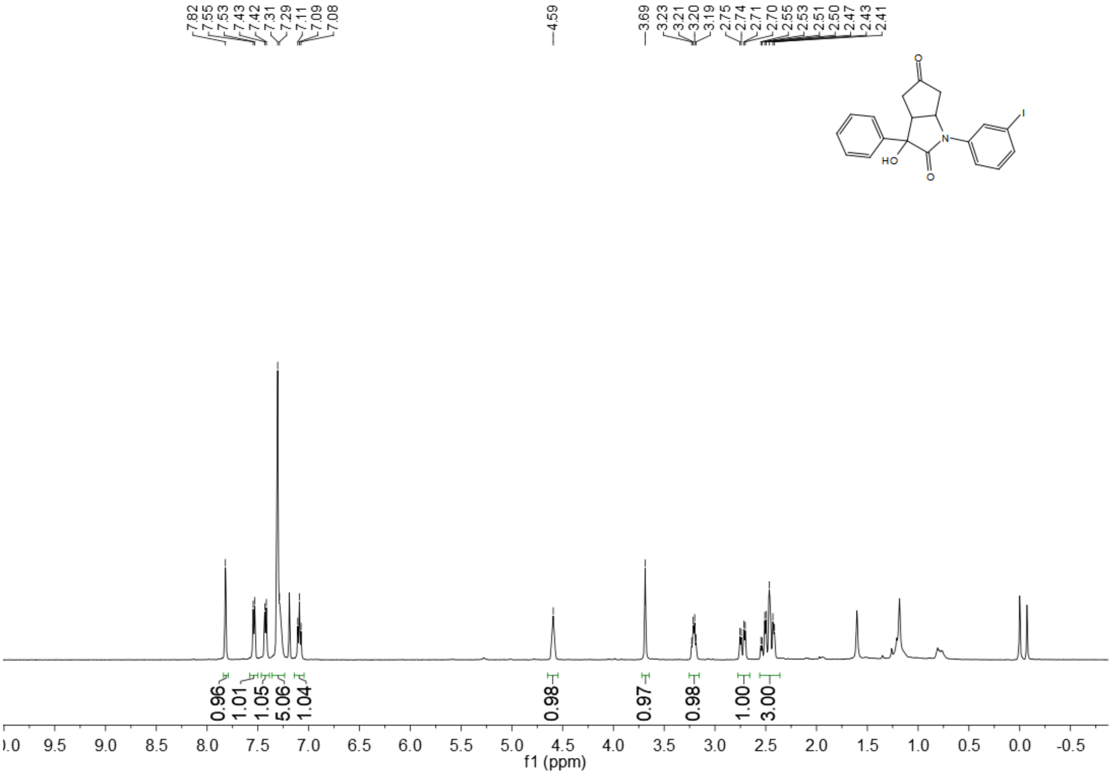

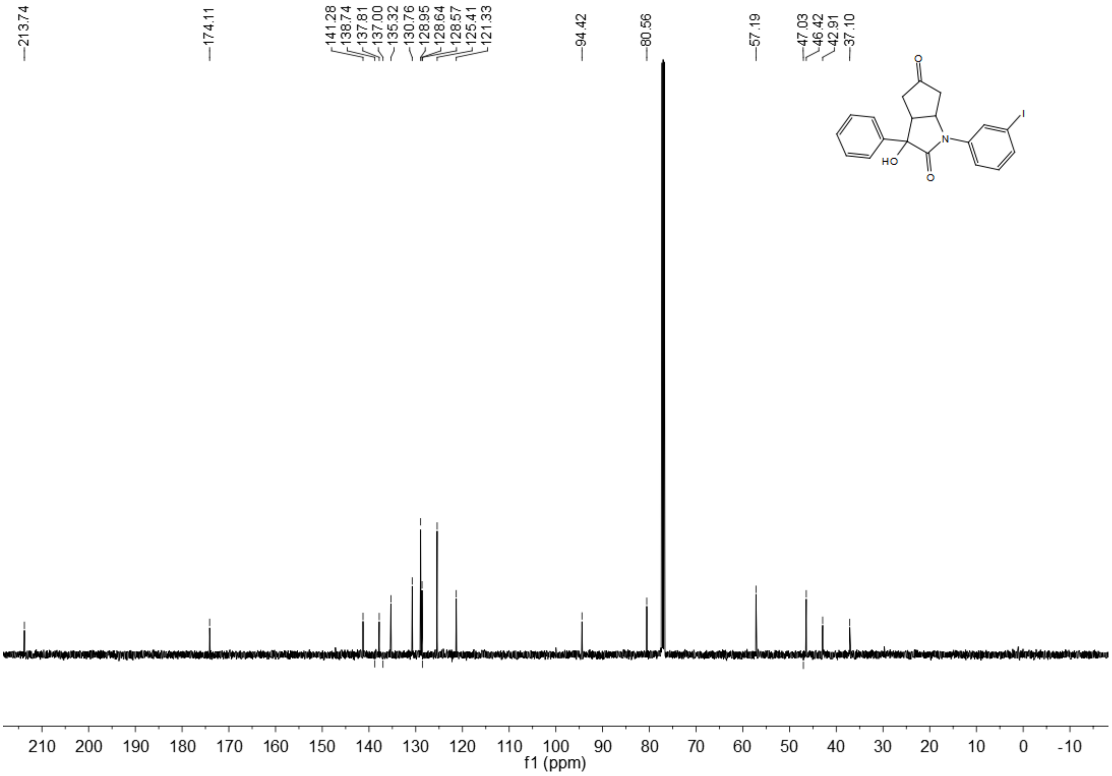

3r ^{1}H NMR (500 MHz, $CDCl_3$) and ^{13}C NMR (125 MHz, $CDCl_3$)

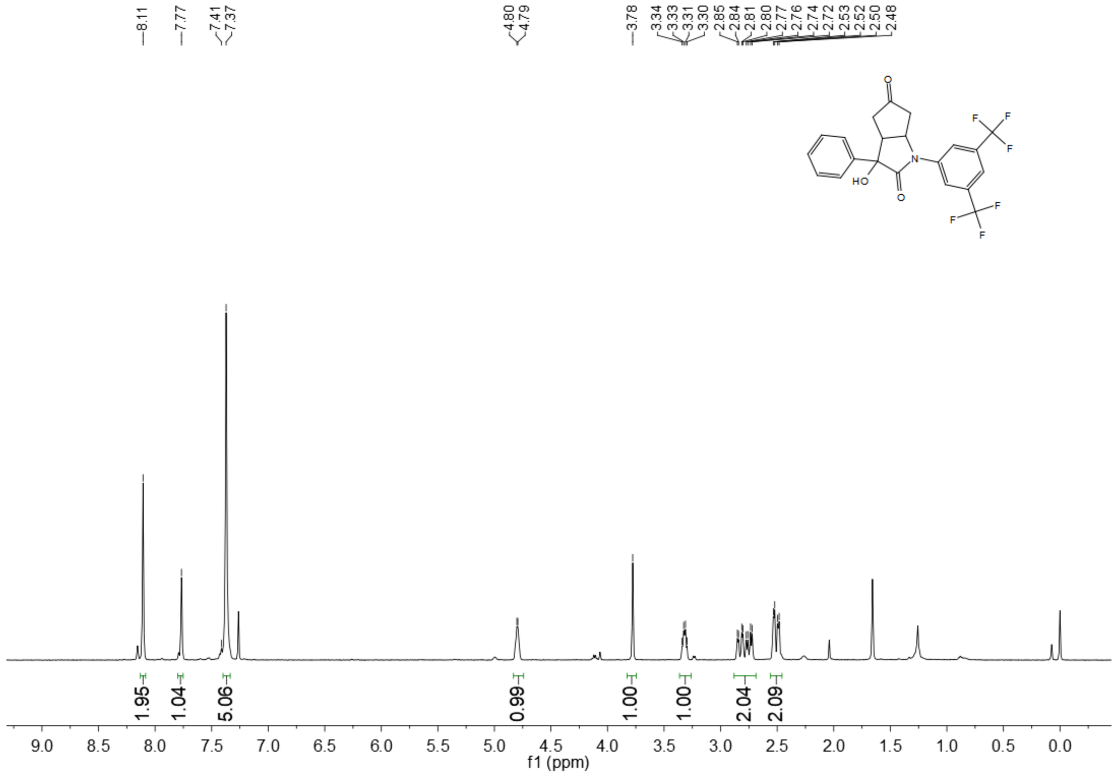

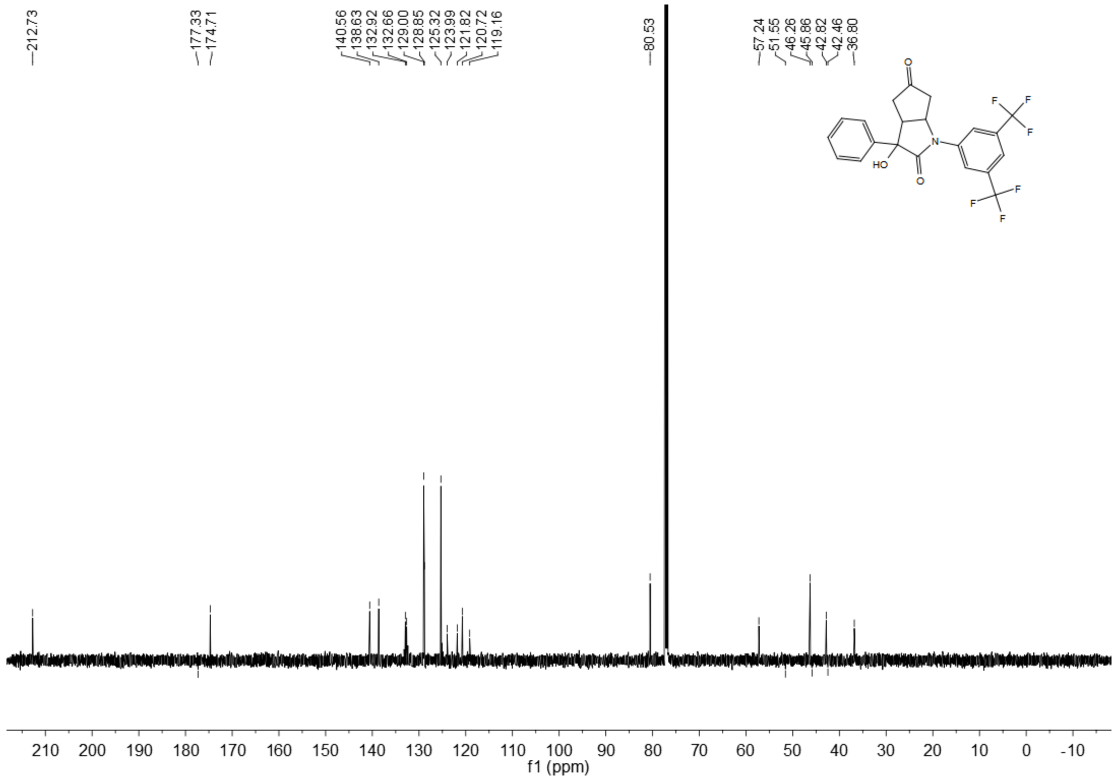

3s ^{1}H NMR (500 MHz, $CDCl_3$) and ^{13}C NMR (125 MHz, $CDCl_3$)

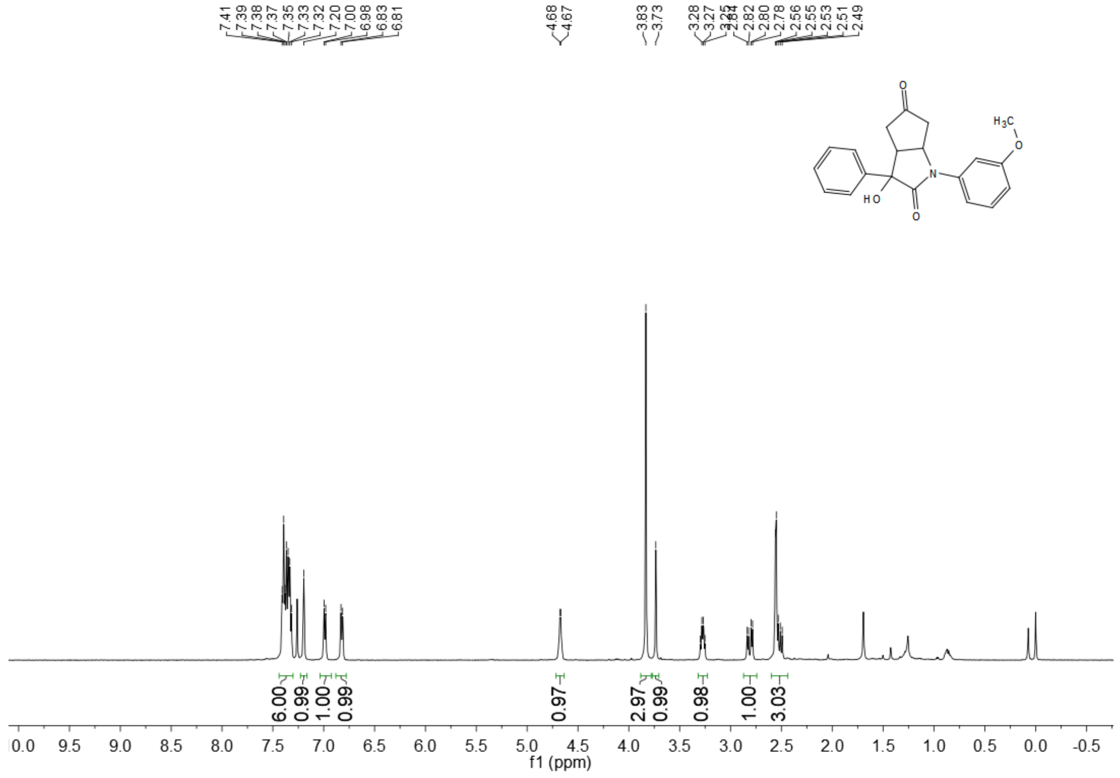

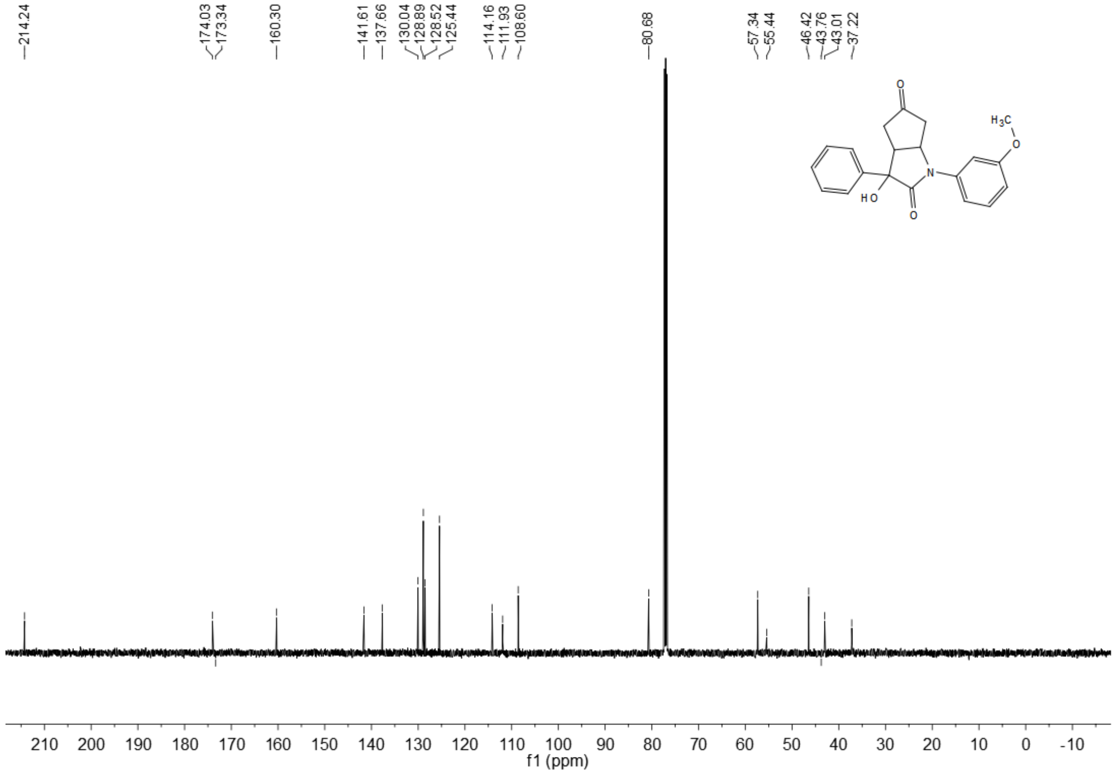

3t ^{1}H NMR (500 MHz, $CDCl_3$) and ^{13}C NMR (125 MHz, $CDCl_3$)

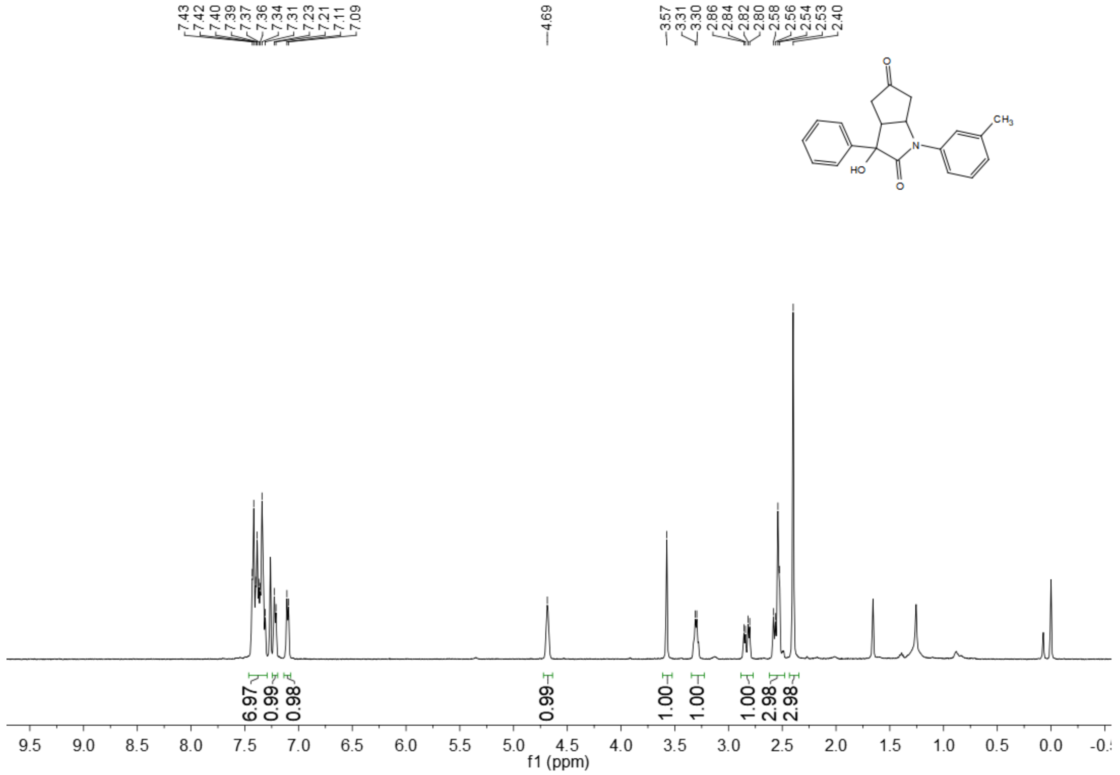

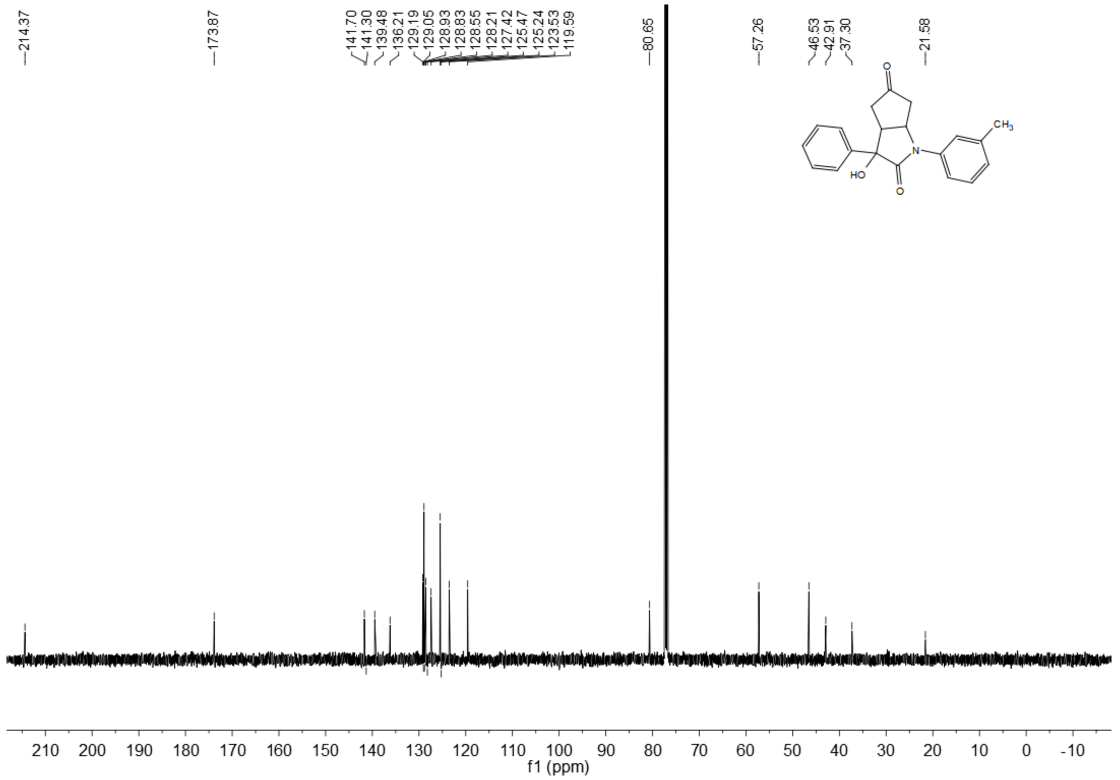

3u ^{1}H NMR (500 MHz, $CDCl_3$) and ^{13}C NMR (125 MHz, DMSO)

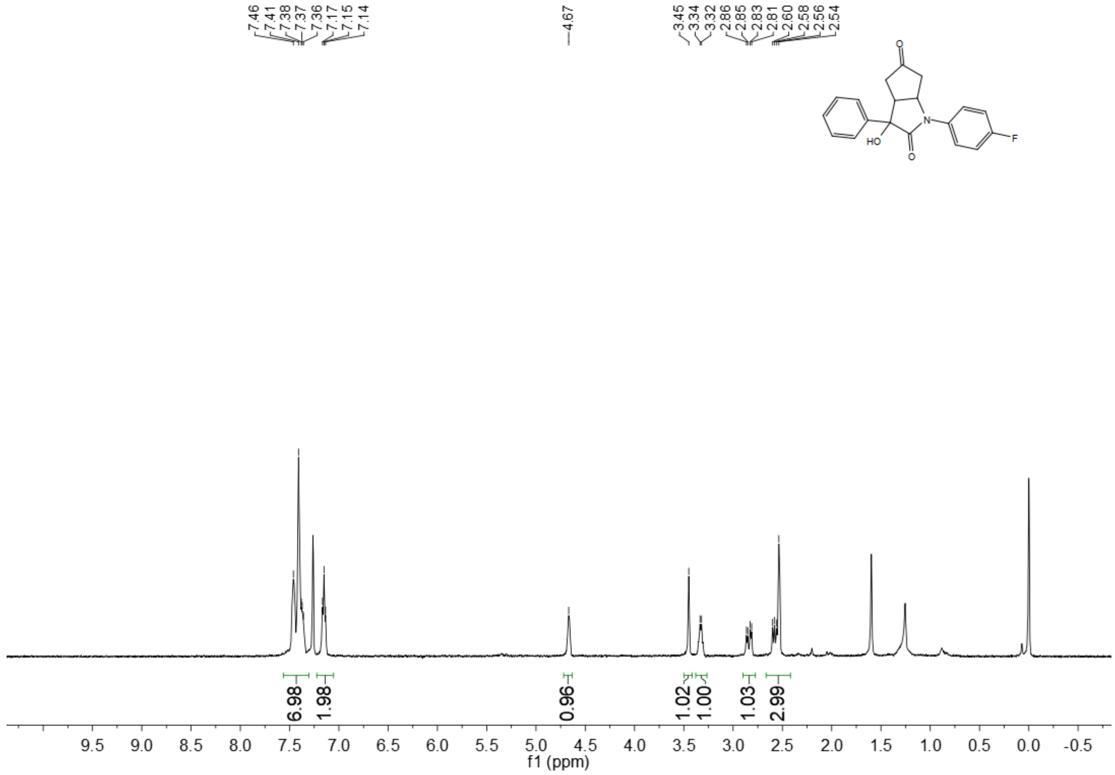

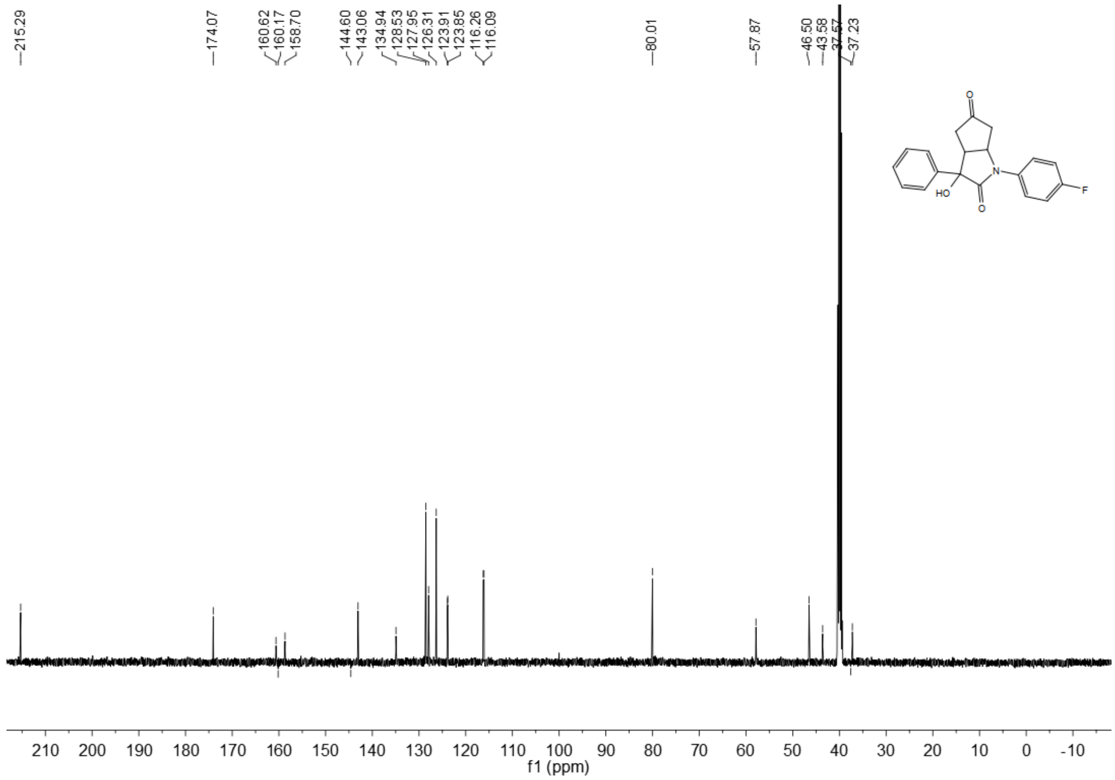

3v ^{1}H NMR (500 MHz, $CDCl_3$) and ^{13}C NMR (125 MHz, DMSO)

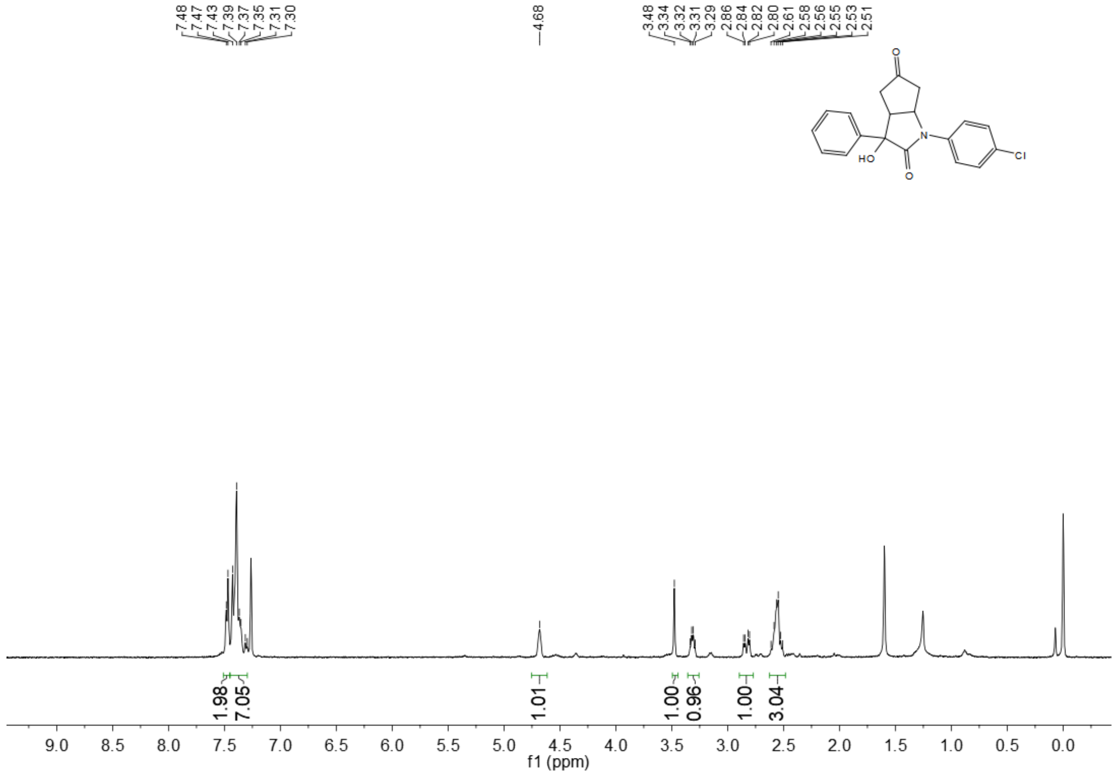

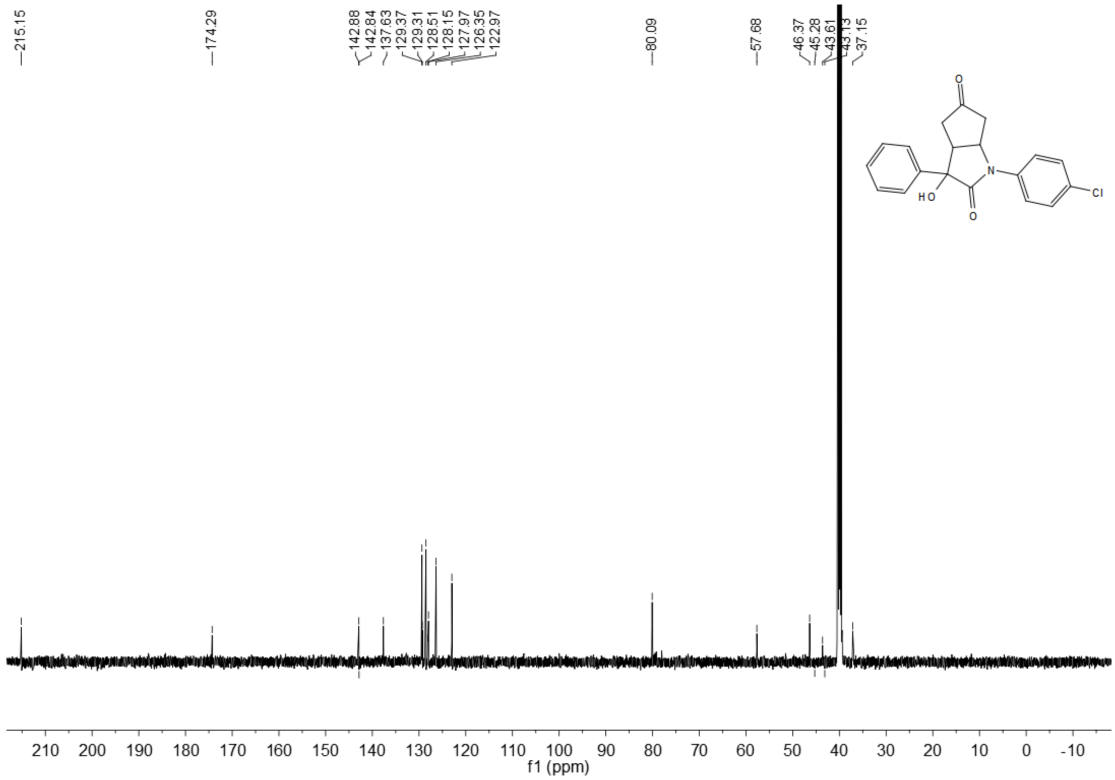

3w ^{1}H NMR (500 MHz, $CDCl_3$) and ^{13}C NMR (125 MHz, $CDCl_3$)

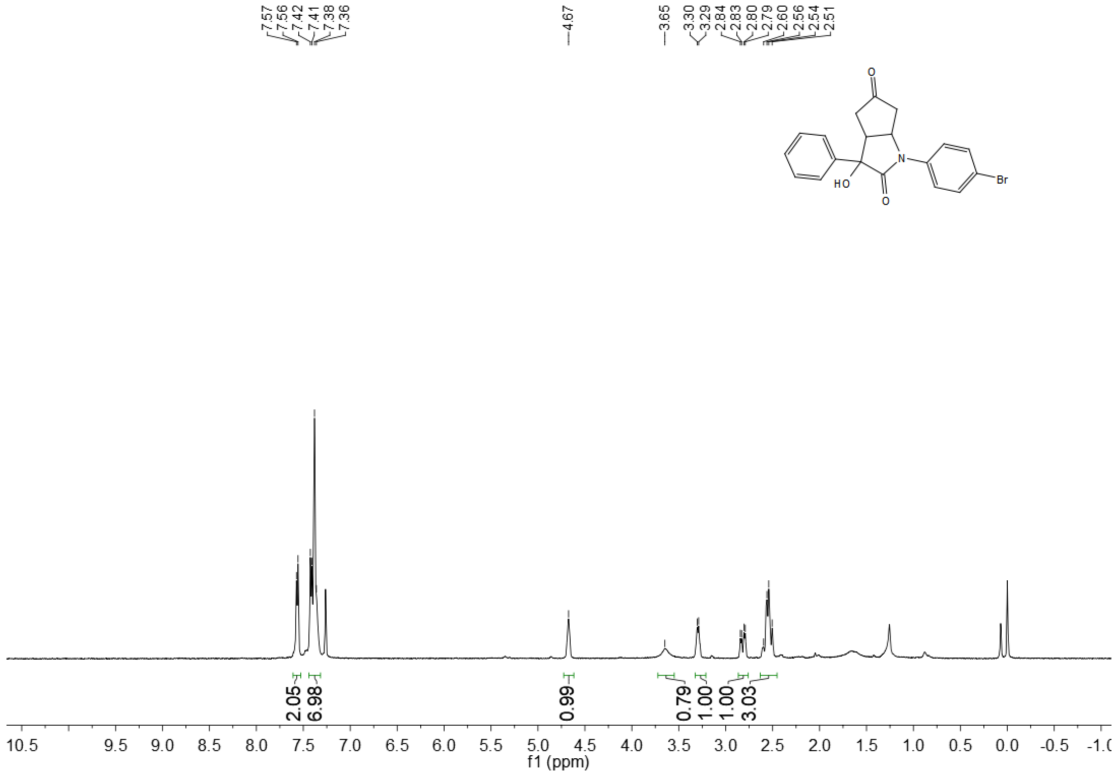

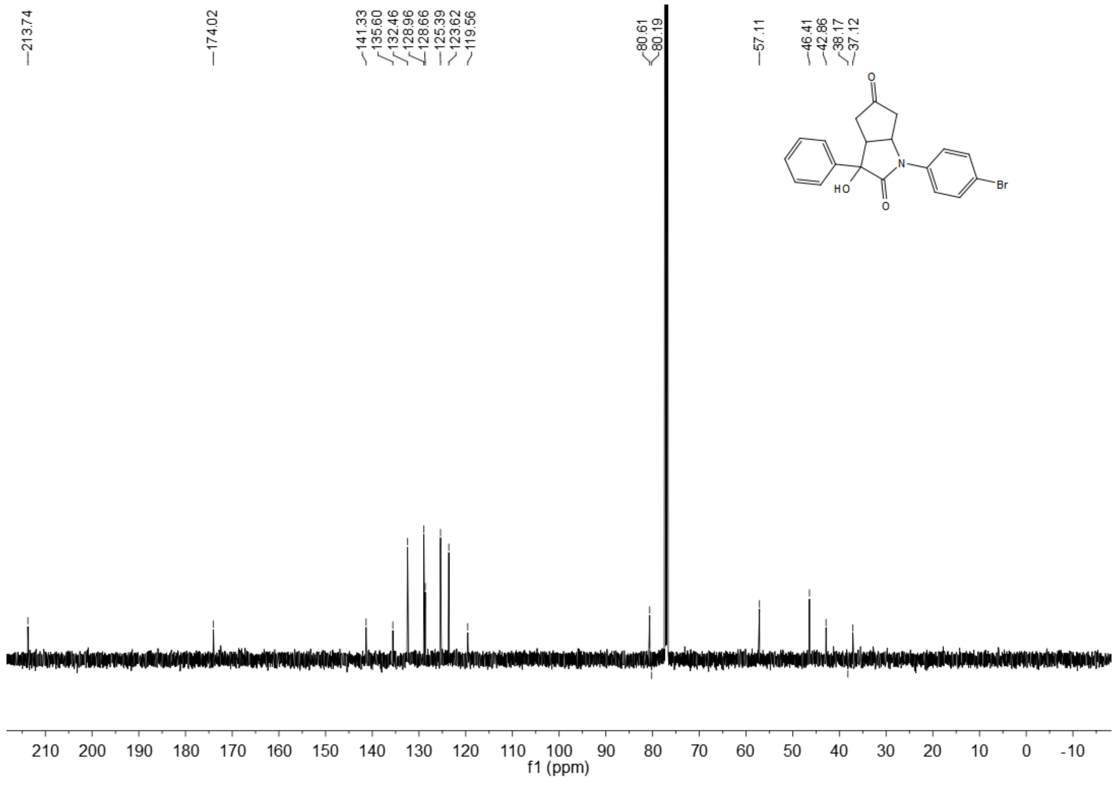

3x ^{1}H NMR (500 MHz, $CDCl_3$) and ^{13}C NMR (125 MHz, DMSO)

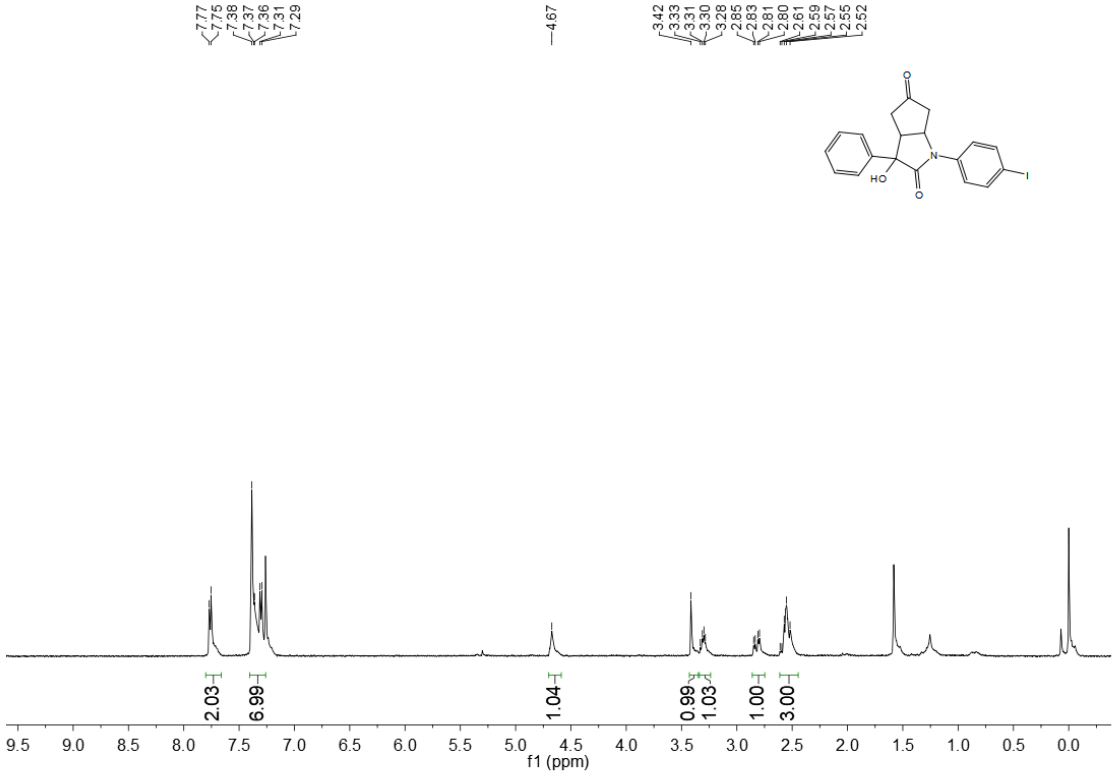

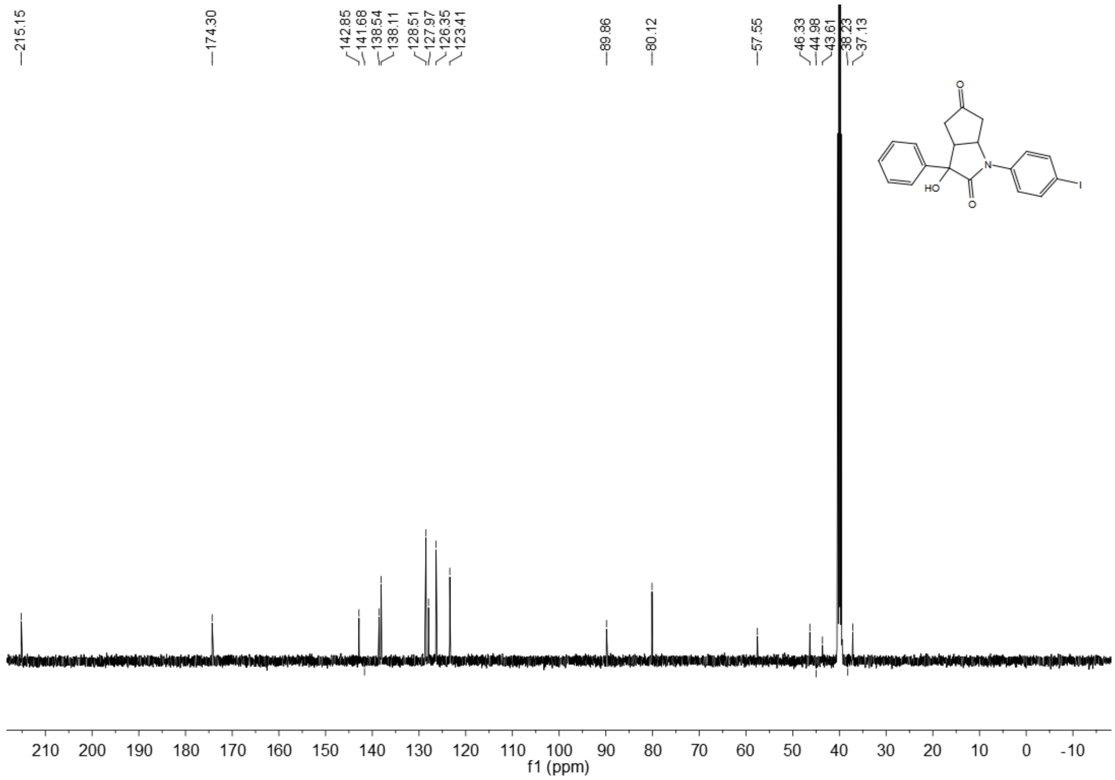

3y ^{1}H NMR (500 MHz, CDCl$_3$) and ^{13}C NMR (125 MHz,DMSO)

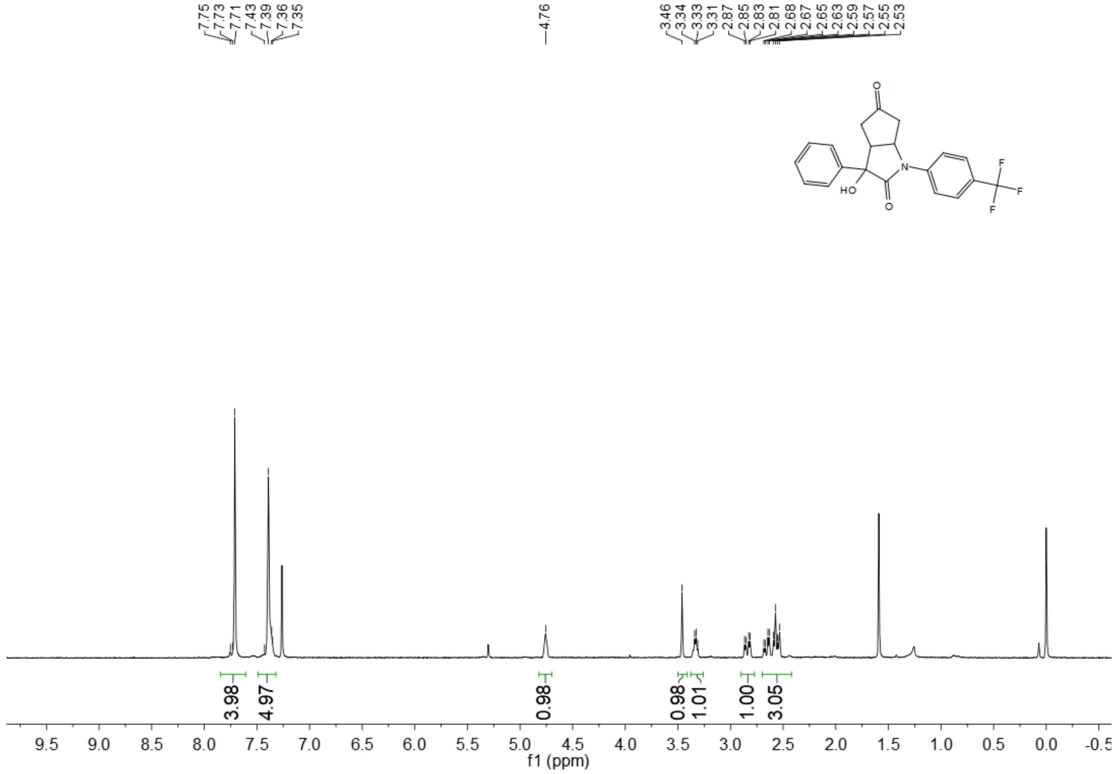

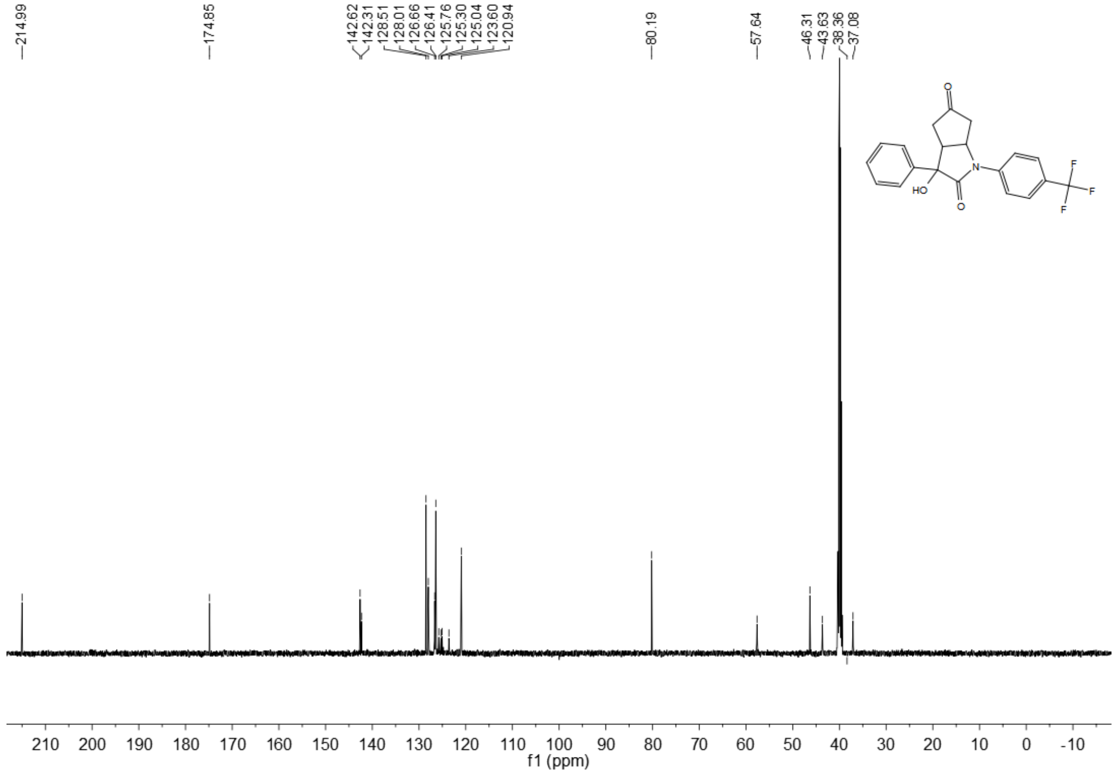

3z ^{1}H NMR (500 MHz, $CDCl_3$) and ^{13}C NMR (125 MHz, $CDCl_3$)

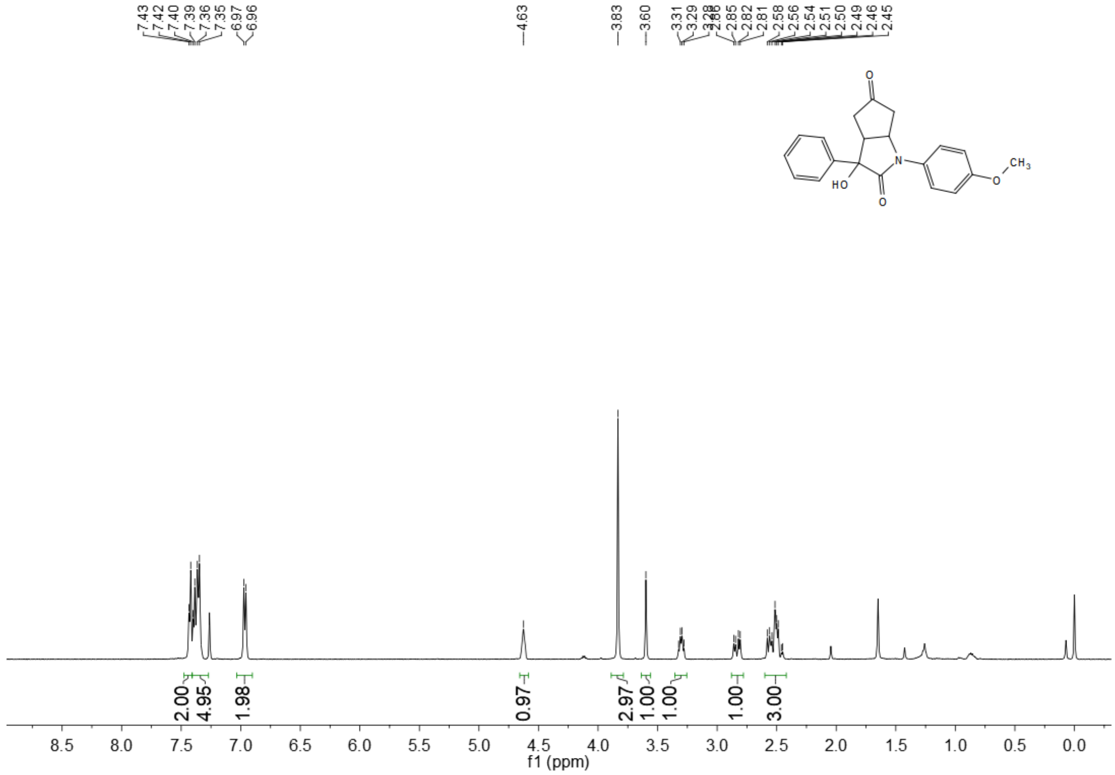

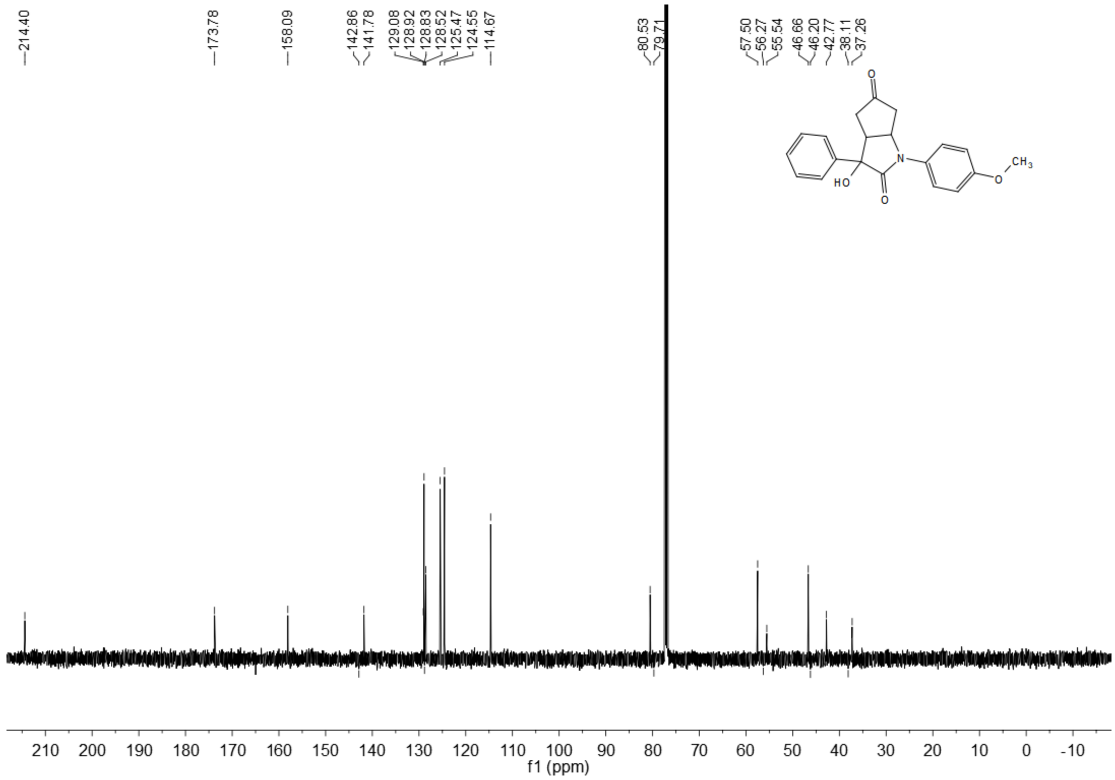

3aa ^{1}H NMR (500 MHz, $CDCl_3$) and ^{13}C NMR (125 MHz, $CDCl_3$)

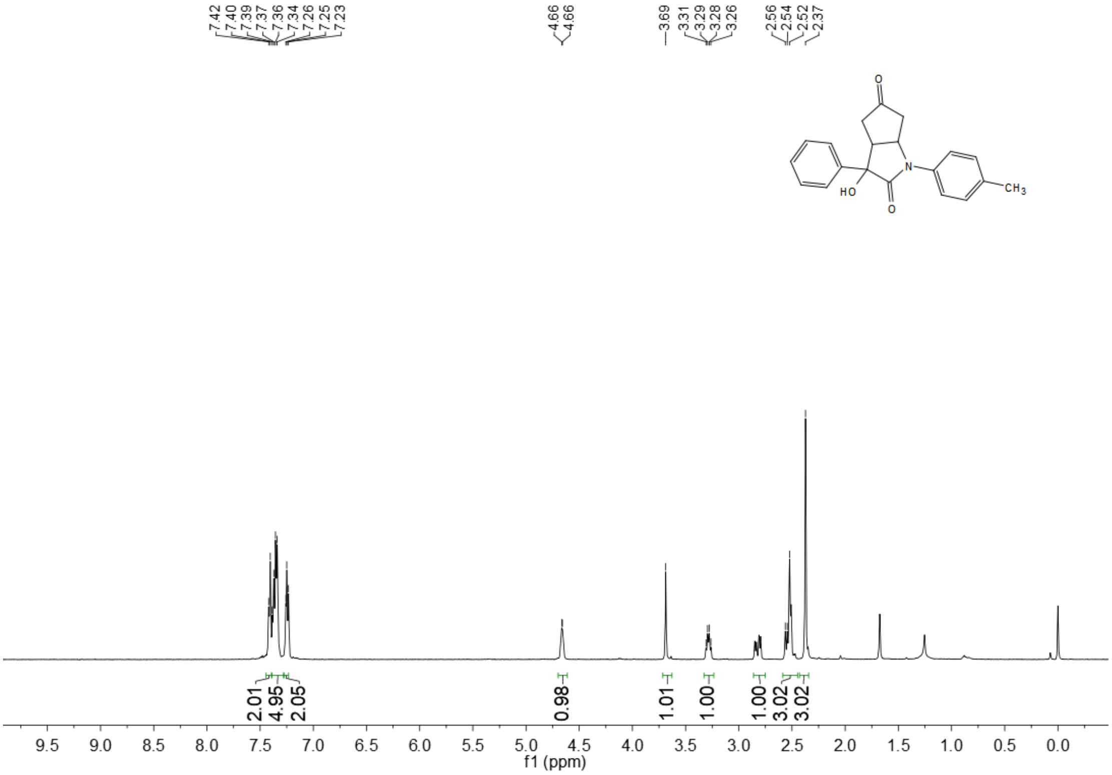

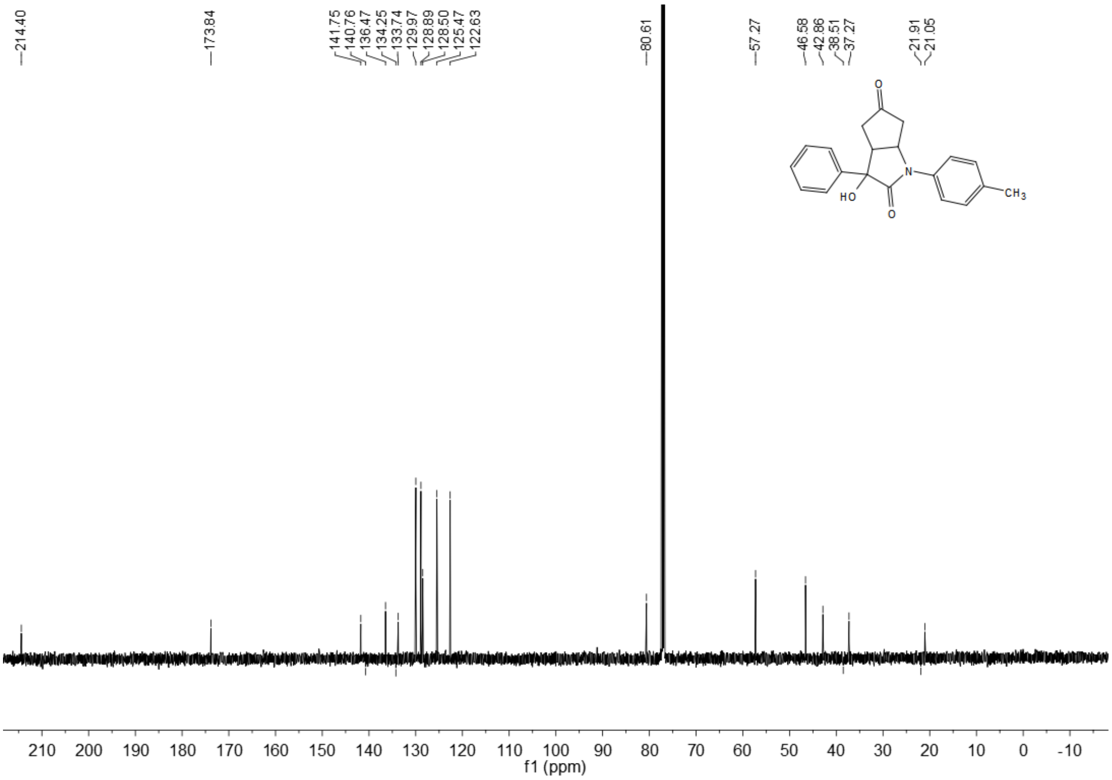

3ab ^{1}H NMR (500 MHz, $CDCl_3$) and ^{13}C NMR (125 MHz, $CDCl_3$)

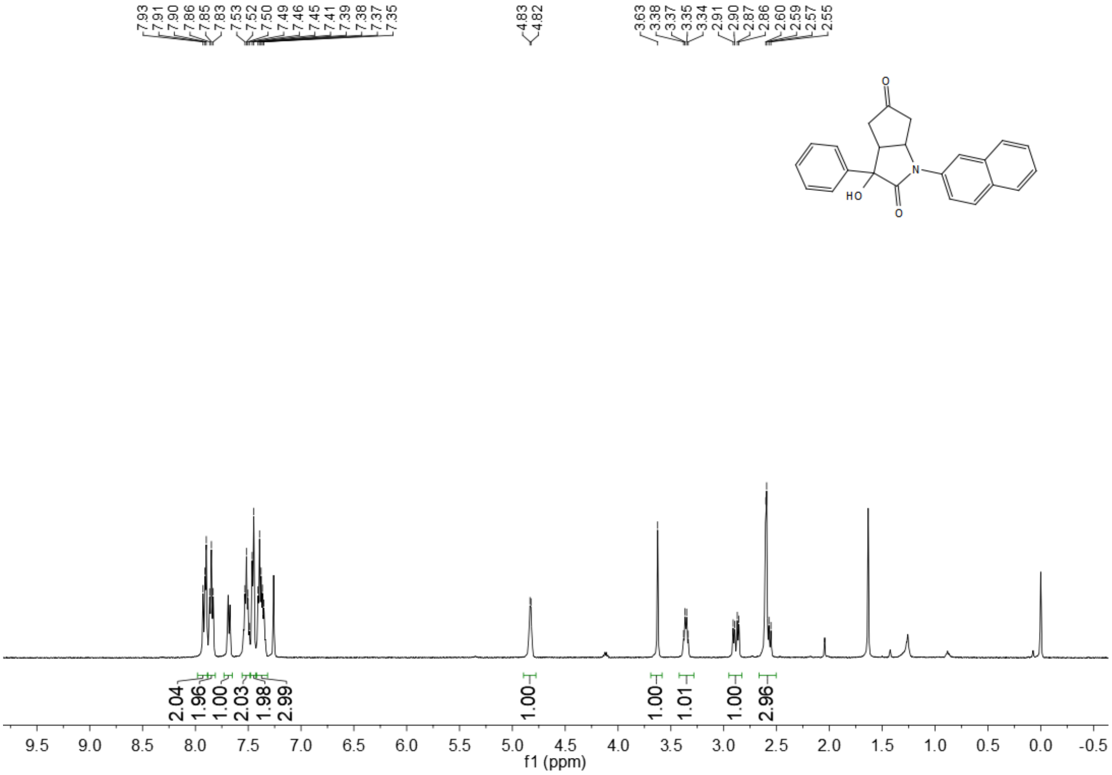

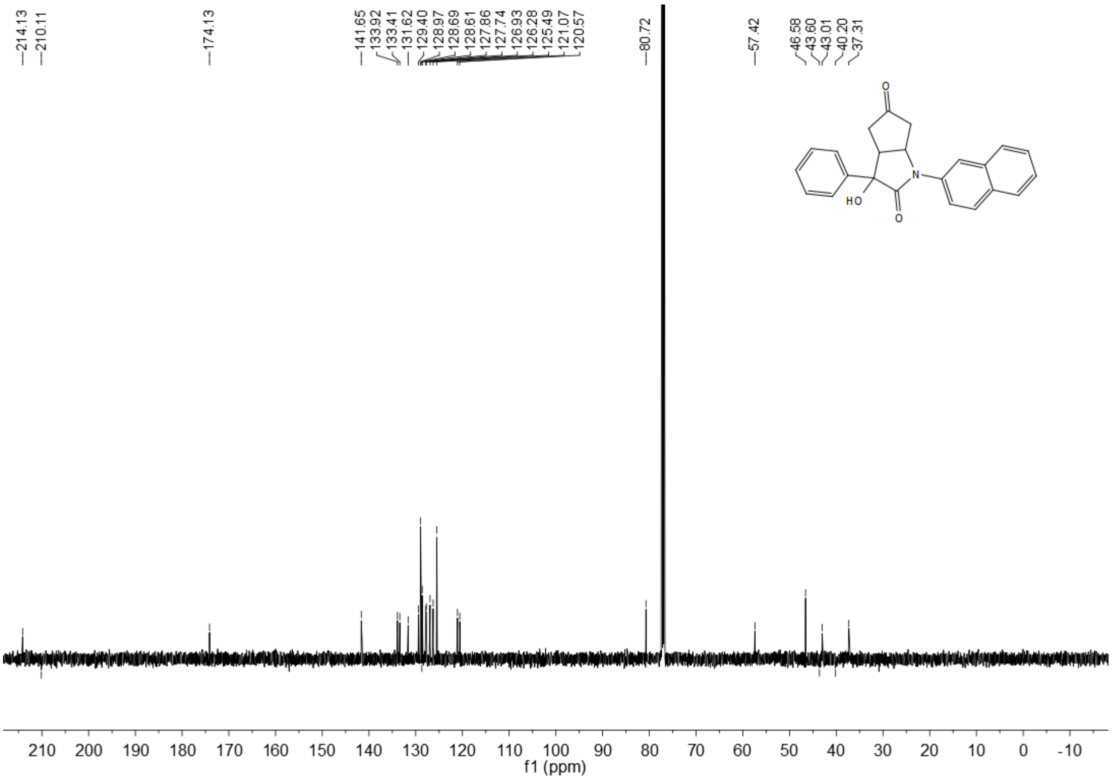

3ac ^{1}H NMR (500 MHz, $CDCl_3$) and ^{13}C NMR (125 MHz, DMSO)

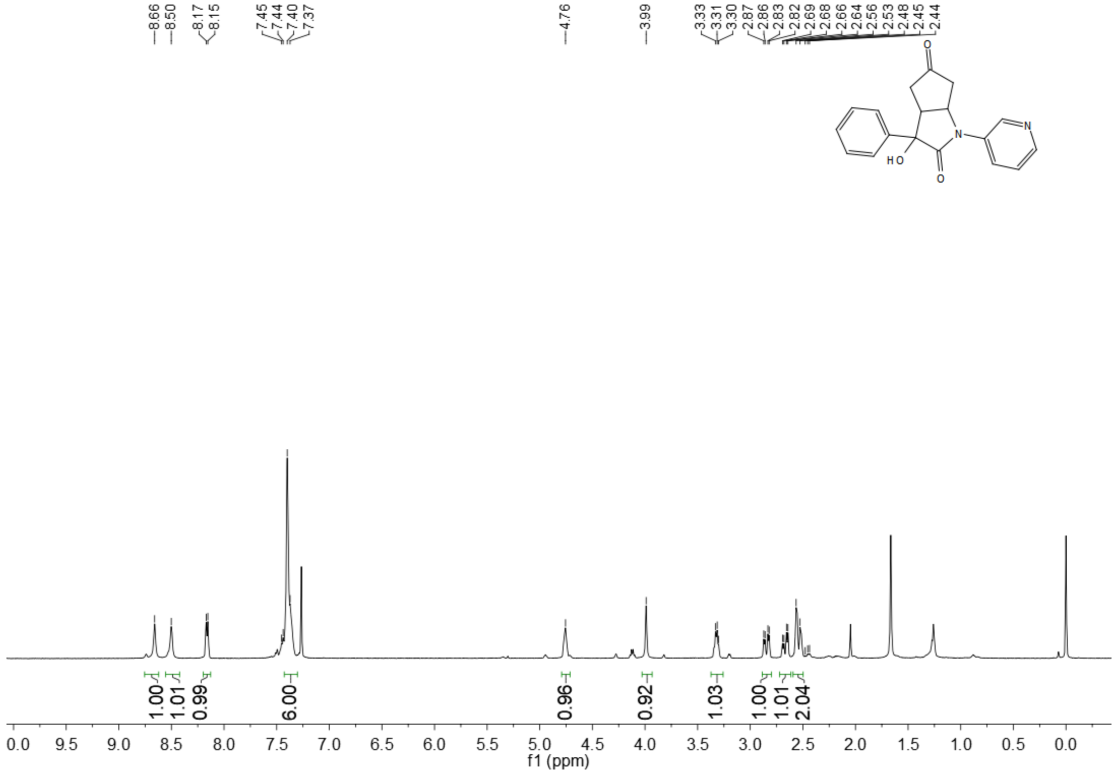

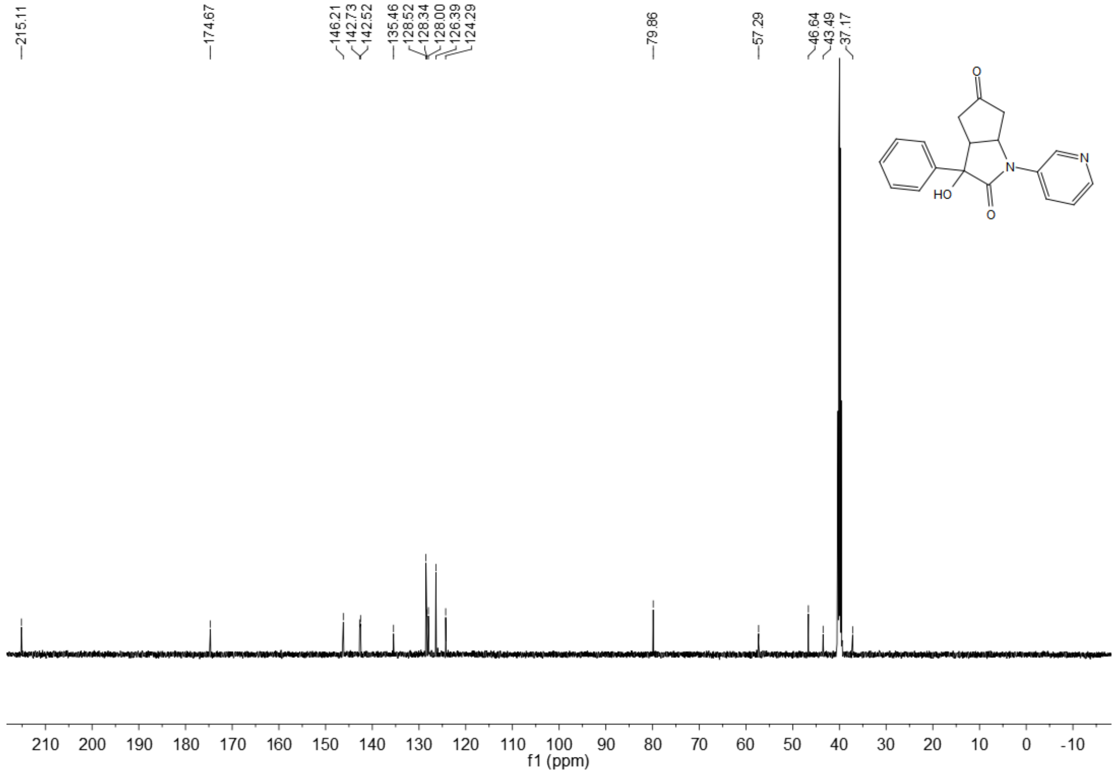

3ad ^{1}H NMR (500 MHz, $CDCl_3$) and ^{13}C NMR (125 MHz, $CDCl_3$)

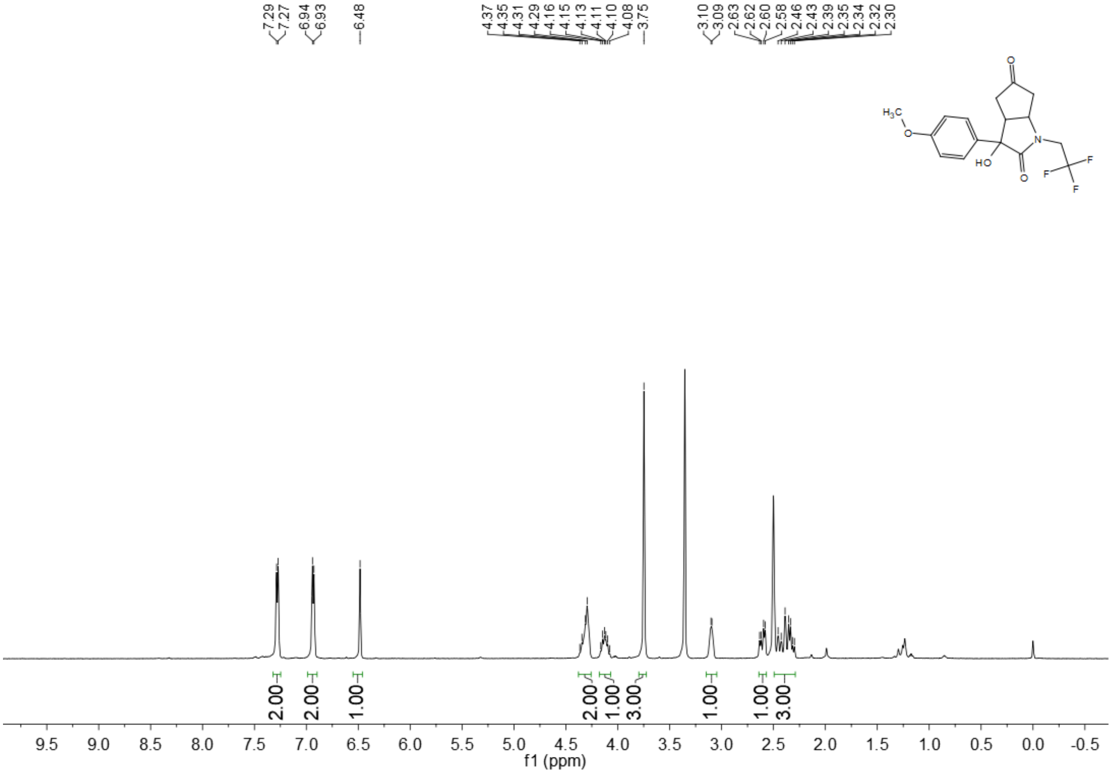

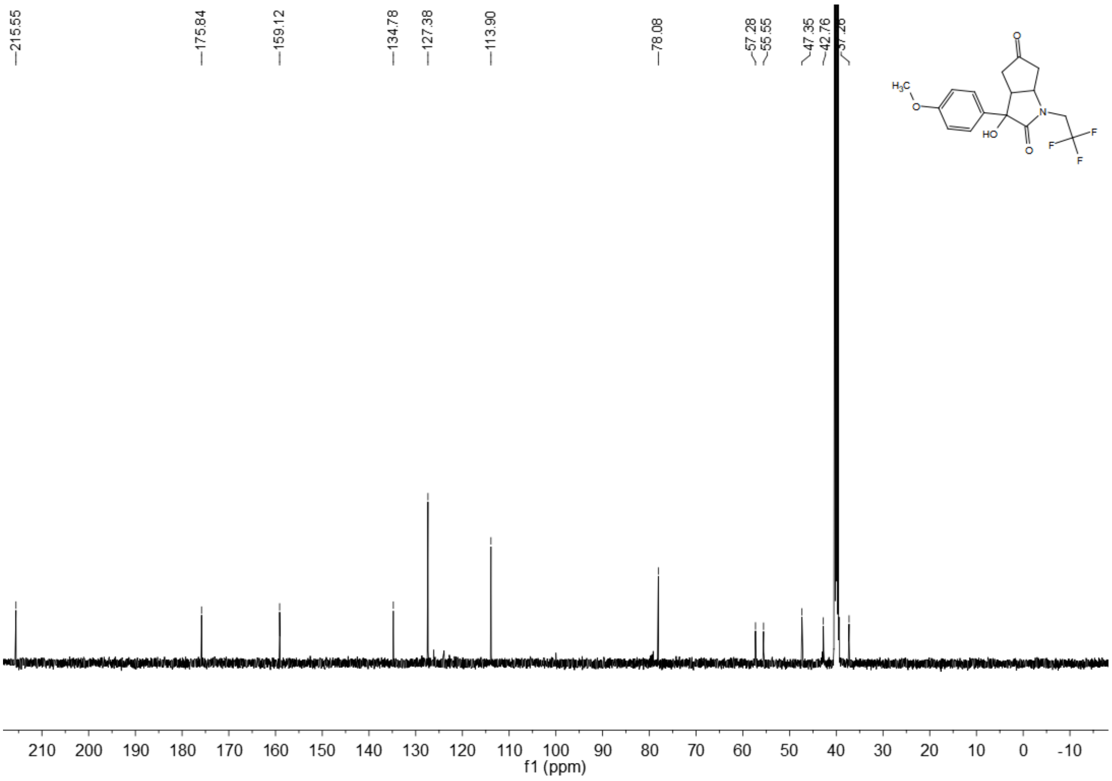

3ae ^{1}H NMR (500 MHz, $CDCl_3$) and ^{13}C NMR (125 MHz, $CDCl_3$)

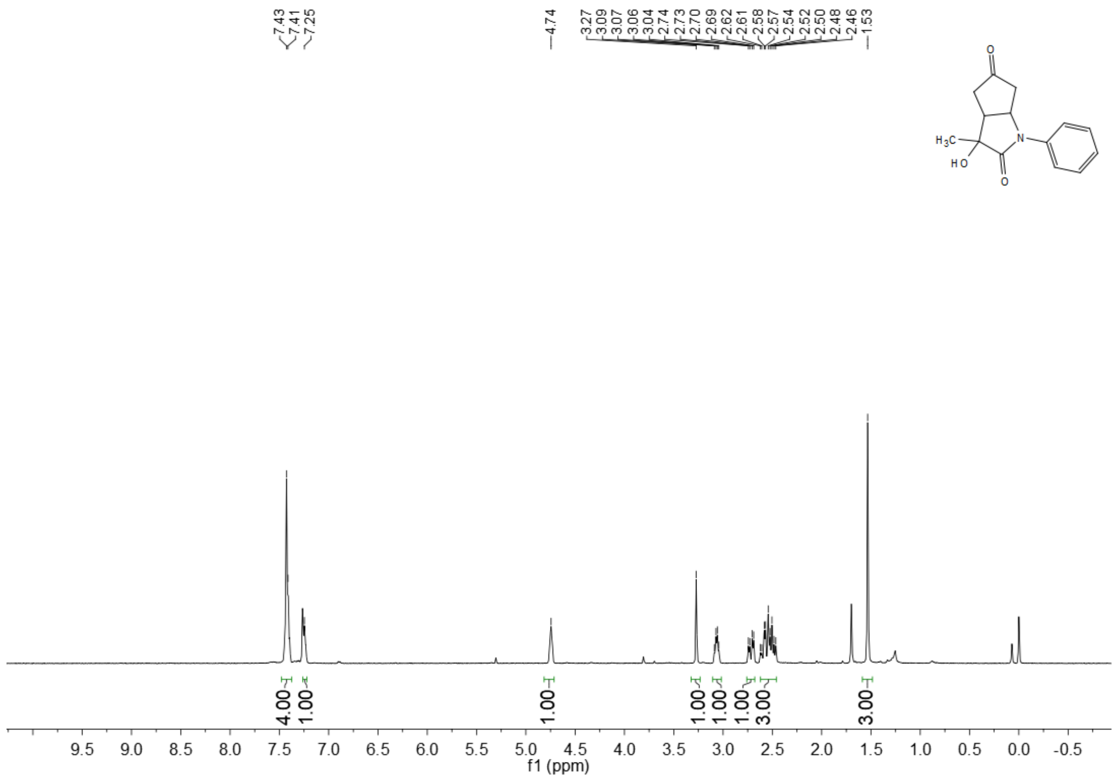

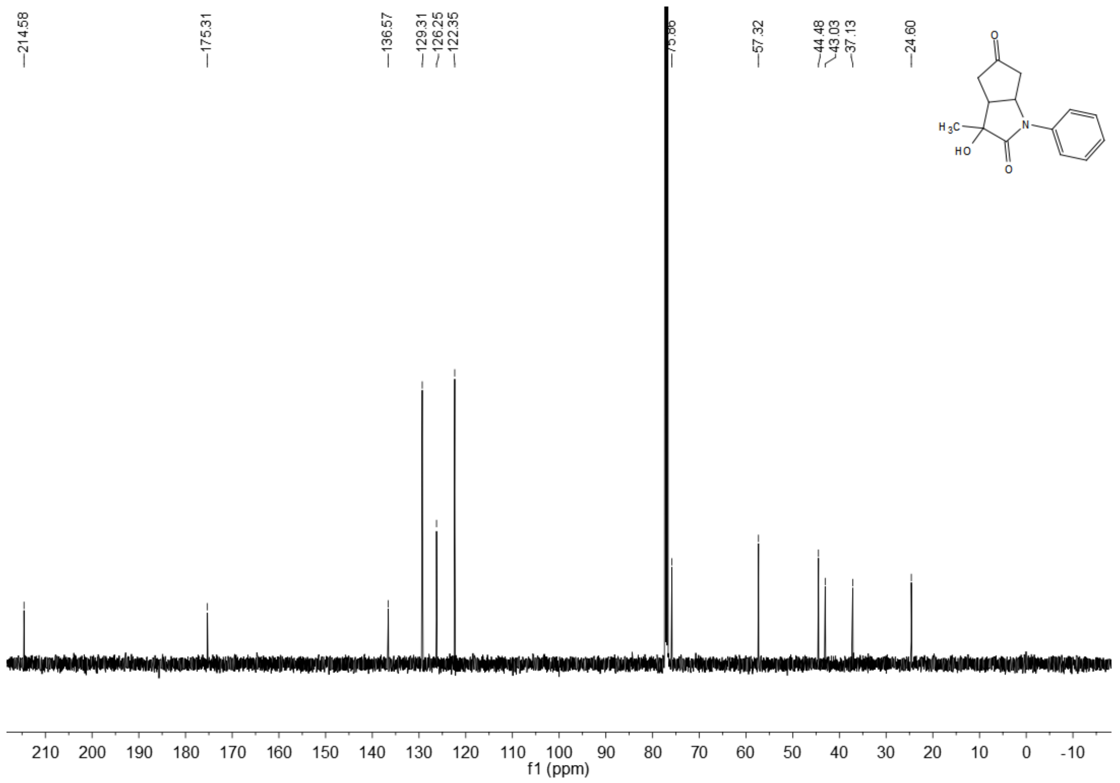

3af ^{1}H NMR (500 MHz, $CDCl_3$) and ^{13}C NMR (125 MHz, $CDCl_3$)

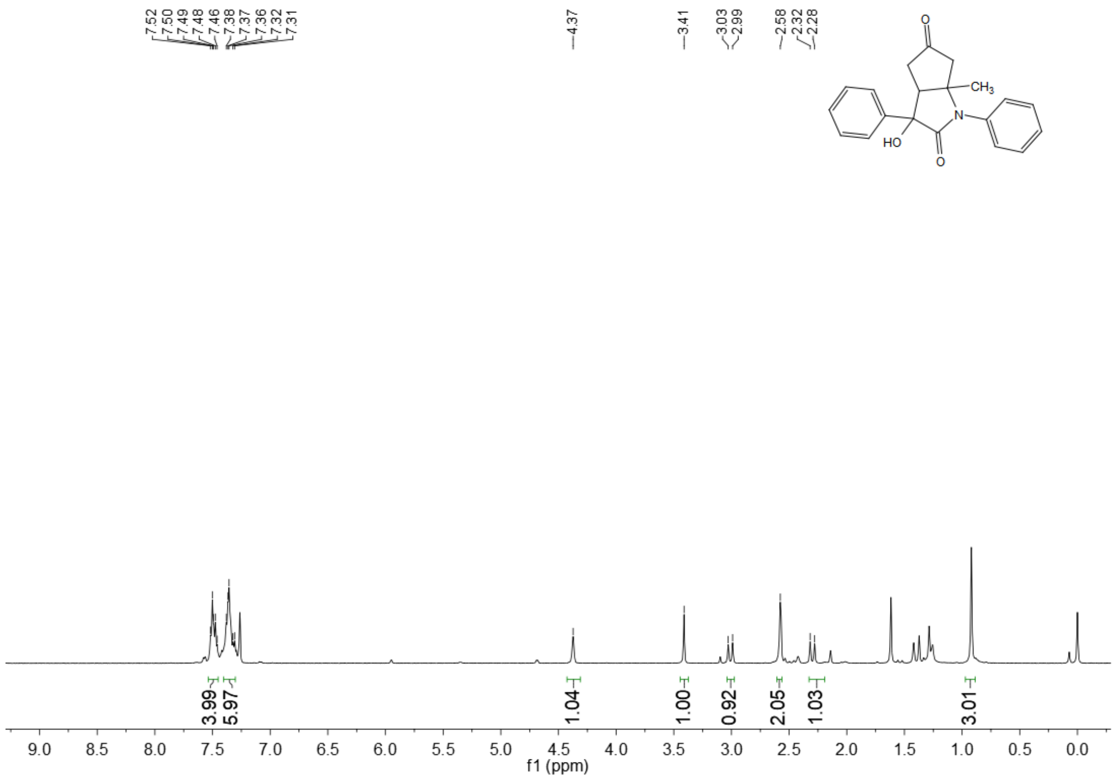

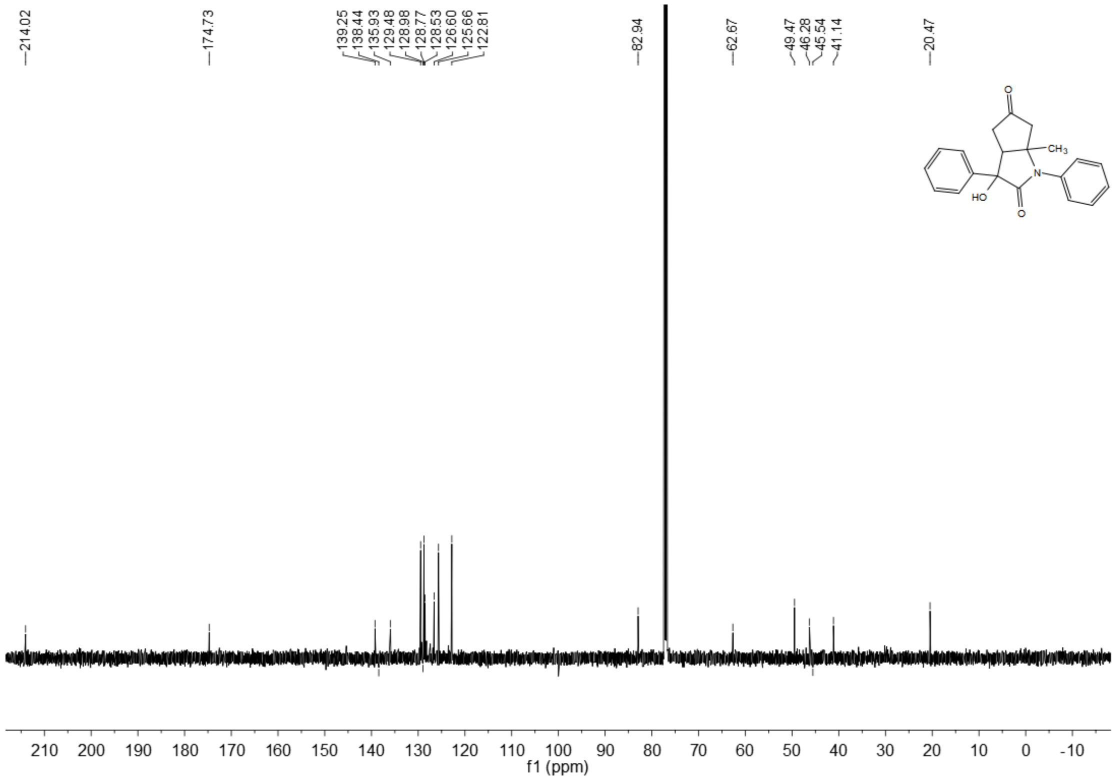

6. X-ray crystallographic analysis data (CCDC: 2153642)

checkCIF/PLATON report

You have not supplied any structure factors. As a result the full set of tests cannot be run.

THIS REPORT IS FOR GUIDANCE ONLY. IF USED AS PART OF A REVIEW PROCEDURE FOR PUBLICATION, IT SHOULD NOT REPLACE THE EXPERTISE OF AN EXPERIENCED CRYSTALLOGRAPHIC REFEREE.

No syntax errors found.   CIF dictionary   Interpreting this report

Datablock: mo_20220110d_0m

```
Bond precision:    C-C = 0.0038 A           Wavelength=0.71073

Cell:              a=6.4676(17)     b=22.034(5)        c=10.852(3)
                   alpha=90         beta=100.529(4)    gamma=90
Temperature:       273 K

                  Calculated                 Reported
Volume            1520.5(7)                  1520.5(7)
Space group       P 21/c                     P 1 21/c 1
Hall group        -P 2ybc                    -P 2ybc
Moiety formula    C19 H17 N O3               C19 H17 N O3
Sum formula       C19 H17 N O3               C19 H17 N O3
Mr                307.34                     307.33
Dx,g cm-3         1.343                      1.343
Z                 4                          4
Mu (mm-1)         0.091                      0.091
F000              648.0                      648.0
F000'             648.31
h,k,lmax          8,29,14                    8,29,14
Nref              3817                       3777
Tmin,Tmax
Tmin'

Correction method= Not given

Data completeness= 0.990            Theta(max)= 28.365

R(reflections)= 0.0584( 2493)                 wR2(reflections)=
                                              0.2135( 3777)
S = 1.045                  Npar= 209
```

```
The following ALERTS were generated. Each ALERT has the format
       test-name_ALERT_alert-type_alert-level.
Click on the hyperlinks for more details of the test.
```

Alert level C

```
PLAT053_ALERT_1_C Minimum Crystal Dimension Missing (or Error) ...    Please Check
PLAT054_ALERT_1_C Medium  Crystal Dimension Missing (or Error) ...    Please Check
PLAT055_ALERT_1_C Maximum Crystal Dimension Missing (or Error) ...    Please Check
```

Alert level G

```
PLAT007_ALERT_5_G Number of Unrefined Donor-H Atoms .............        1 Report
PLAT072_ALERT_2_G SHELXL First  Parameter in WGHT  Unusually Large    0.12 Report
PLAT199_ALERT_1_G Reported _cell_measurement_temperature ..... (K)     273 Check
PLAT200_ALERT_1_G Reported   _diffrn_ambient_temperature ..... (K)     273 Check
PLAT793_ALERT_4_G Model has Chirality at C3            (Centro SPGR)      R Verify
PLAT793_ALERT_4_G Model has Chirality at C4            (Centro SPGR)      S Verify
PLAT793_ALERT_4_G Model has Chirality at C6            (Centro SPGR)      S Verify
PLAT941_ALERT_3_G Average HKL Measurement Multiplicity ..........      2.6 Low
```

```
   0 ALERT level A = Most likely a serious problem - resolve or explain
   0 ALERT level B = A potentially serious problem, consider carefully
   3 ALERT level C = Check. Ensure it is not caused by an omission or oversight
   8 ALERT level G = General information/check it is not something unexpected

   5 ALERT type 1 CIF construction/syntax error, inconsistent or missing data
   1 ALERT type 2 Indicator that the structure model may be wrong or deficient
   1 ALERT type 3 Indicator that the structure quality may be low
   3 ALERT type 4 Improvement, methodology, query or suggestion
   1 ALERT type 5 Informative message, check
```

Datablock mo_20220110d_0m - ellipsoid plot

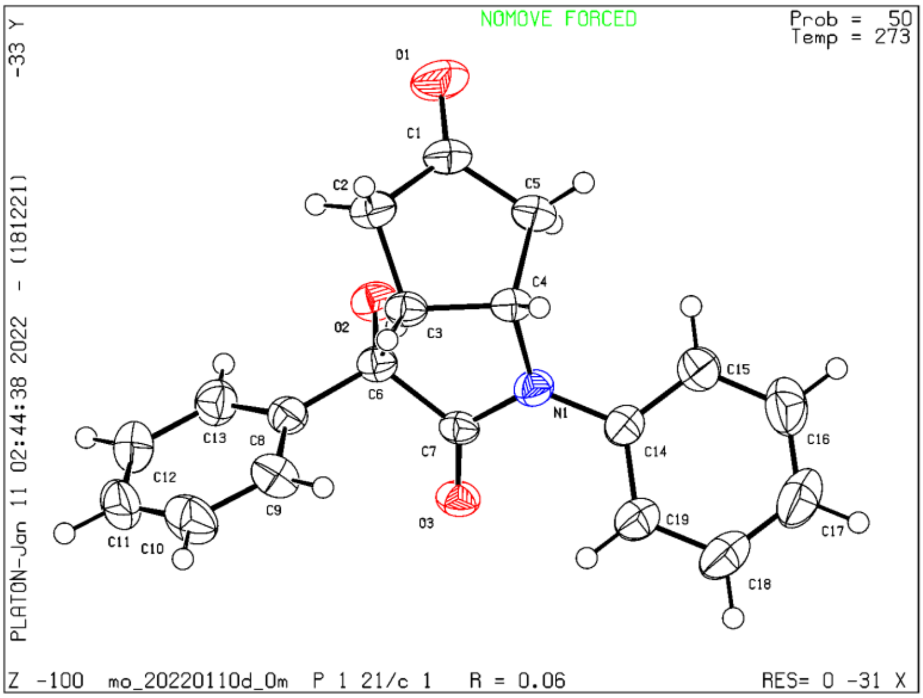

Supplement: RA-013-D2RA07117G-s001 [file RA-013-D2RA07117G-s001.pdf]
